# Modern Quantum Chemistry Methodology for Predicting ^31^P Nuclear Magnetic Resonance Chemical Shifts

**DOI:** 10.3390/ijms27020704

**Published:** 2026-01-09

**Authors:** Irina L. Rusakova, Yuriy Yu. Rusakov

**Affiliations:** A. E. Favorsky Irkutsk Institute of Chemistry, Siberian Branch of the Russian Academy of Sciences, Favorsky St. 1, 664033 Irkutsk, Russia; rusakov82@mail.ru

**Keywords:** ^31^P NMR, phosphorus, quantum chemistry, basis sets, geometry factor effect, solvent effects, vibrational effects, relativistic effects

## Abstract

Phosphorus-31 nuclear magnetic resonance (^31^P NMR) spectroscopy is a powerful analytical physical chemistry experimental technique that is widely used to study the structure and dynamics of phosphorus-containing compounds today. Accurate calculation of ^31^P NMR chemical shifts lies in the basis of the proper assignment of NMR signals, as they can be closely spaced to each other in the NMR spectra of systems that bear nuclei with subtly different electron environments, like complex organophosphorus compounds, nucleic acids, and phosphates, etc. The most advanced quantum chemistry (QC) methods allow us to reach the agreement between theoretical values of ^31^P NMR chemical shifts and experiments within a few ppm, which makes them a useful tool for studying chemical structure, reaction mechanisms, and catalyst design with the aid of the NMR method. This review surveys the application of both density functional and wave function methods of electron structure to the calculation of ^31^P NMR chemical shifts and proposes a thorough discussion of the latest findings related to the factors affecting the final accuracy of the ^31^P NMR chemical shifts prediction, including basis sets, the geometry factor effect, solvent, vibrational, and relativistic corrections.

## 1. Introduction

The synthesis of new phosphorus compounds is inevitably accompanied by physical–chemical methods allowing us to confirm their structure. One of the most powerful physical–chemical methods for studying the structure and dynamics of phosphorus compounds is ^31^P NMR spectroscopy. This has become very popular experimental method as the ^31^P isotope is a highly sensitive nucleus with a spin of 1/2 and natural abundance of 100%, which results in significantly shorter acquisition time and concentrations needed for the NMR experiment. Its NMR chemical shift scale is rather wide, typically covering the range from −350 to +300 ppm relative to 85% aqueous solution of phosphoric acid H_3_PO_4_. At that, ^31^P NMR chemical shifts are extremely sensitive to the electronic structure; therefore, even a small change in the latter significantly affects the ^31^P NMR spectra [1]. Thanks to these highly favorable features of the phosphorus-31 isotope, ^31^P NMR spectroscopy has become an excellent tool for the structural analysis of phosphorus-containing compounds.

Quantum chemical simulation of ^31^P NMR spectra is usually needed when complex phosphorus-containing molecules like nucleic acids or phosphates give an entangled ^31^P NMR spectrum, which makes it difficult to assign NMR signals to specific groups of atoms and confirm the presumed structure, especially when it comes to distinguishing between isomers with subtle electronic structure differences.

The most advanced quantum chemistry (QC) methodologies allow us to reach agreement between the theoretical values of ^31^P NMR chemical shifts and experimental values within a few ppm, thus making it possible to resolve even the most entangled NMR structural problems. However, QC calculations of phosphorus chemical shifts represent a great challenge due to their essential sensitivity to mostly all factors of accuracy, including the level of electron theory, the quality of the basis set used, the dependence on the precision of equilibrium geometry parameters (the geometry factor effect), the level of consideration of solvent, vibrational, and relativistic corrections, and many other issues.

The present review covers selected works up to the end of 2025, which, to the most extent, facilitated the development of a computational methodology for ^31^P NMR shielding constants/chemical shifts. For the sake of integrity, the review starts with a brief theoretical section, which explains the main notions and concepts extensively used in the main body of the manuscript. Then, the discussion of quantum chemical calculations of ^31^P NMR chemical shifts is presented, starting from early single efforts to modern computational works. The other sections of the review are devoted to the main computational factors affecting the accuracy of the ^31^P NMR chemical shift calculations. These include the discussion of specialized basis sets, the geometry factor effect, and solvent, vibrational, and relativistic effects.

## 2. Theoretical Basis for ^31^P NMR Chemical Shift Calculations

### 2.1. Quantum Chemical Representation of NMR Shielding Tensors

The original expression for NMR shielding tensors was derived by Ramsey [2], on the basis of the second-order Rayleigh–Schrodinger perturbation theory, without taking into account the effects of special relativity. It was proposed that, for the measurement of nuclear magnetic moments, a correction must be made for the magnetic field arising from the motions of molecular electrons due to the externally applied magnetic field. Thus, the local magnetic field at the position of the nucleus represents a superposition of the external magnetic field **B** and the magnetic field induced by the electron currents **B**_i**nd**_, which is proportional to the former through the so-called nuclear shielding constant:

(1) $$B_{ind}=-\sigma B$$

For closed-shell systems, **B**_ind_ is strongly anti-parallel to **B** at the position of the nucleus; so, the former weakens the latter.

In order to take this phenomenon into account, Ramsey suggested an extension of the Schrödinger Hamiltonian to include hyperfine interactions between the nuclei and magnetic field. Its structure can be expressed as follows:

(2) $$\hat{H}\left(B,\mu_{N}\right)={\hat{H}}_{0}+{\hat{H}}^{10}B+\frac{1}{2}B^{T}{\hat{H}}^{\left(20\right)}B+\sum_{N}\mu_{N}{\hat{H}}_{N}^{\left(01\right)}+\frac{1}{2}\sum_{N}\mu_{N}^{T}{\hat{H}}_{N}^{\left(11\right)}B\;+\frac{1}{2}\sum_{NM}\mu_{N}^{T}{\hat{H}}_{N}^{\left(02\right)}\mu_{M}+\ldots$$

In Equation (2), ${\hat{H}}_{0}$ represents the Schrödinger Hamiltonian; ${\hat{H}}^{\left(nl\right)}$ represents the interaction operator containing the *n*-th power of **B** and *l*-th power of $\mu_{N}$, i.e., the magnetic moment of nucleus *N*.

The wave function of a system is expanded into a power series of quantities **B** and $\mu_{N}$; thus, the energy of a system, represented as the average of system Hamiltonian (2), can also be expressed in terms of the power series of **B** and $\mu_{N}$. Thus, by definition, the NMR shielding tensor can be expressed through the energy *E*(**B**, $\mu_{N}$) as its second derivative with respect to the Cartesian components of an external magnetic flux density *B*_α_ and that of the nuclear magnetic moment of nucleus under interest $\mu_{N;\beta }$**,** with the magnetic field and all other nuclear magnetic moments being put to zero:

(3) $$\sigma_{N;\alpha \beta }={\left.\frac{\partial^{2}E(B,\mu_{N})}{\partial B_{\alpha }\partial \mu_{N;\beta }}\right|}_{\mu_{N}=0;\;B=0}$$

The tensor, $\sigma_{N;\alpha \beta }$, can be split apart into two components that are different from a physical point of view, namely, the diamagnetic and paramagnetic contributions:

(4) $$\sigma_{N;\alpha \beta }=\sigma_{N;\alpha \beta }^{dia}+\sigma_{N;\alpha \beta }^{para}$$

The diamagnetic contribution, $\sigma_{N;\alpha \beta }^{dia}$, represents a molecular counterpart to Lamb’s formula [3] for the screening of an atom, which is proportional to the electron density around the nucleus. This is expressed as the mean value of the diamagnetic operator over the ground state of the unperturbed system $\left|\left.0\right\rangle \right.$ [4]:

(5) $${\overset{⃡}{\sigma }}_{N}^{dia}=C^{dia}\left\langle 0|\sum_{i}\left(r_{i0}^{T}r_{iN}I_{3}-r_{i0}r_{iN}^{T}\right)r_{iN}^{-3}|0\right\rangle$$

The vectors $r_{i0}$ and $r_{iN}$ in Equation (5) represent the radius-vectors of *i*-th electron relative to the center of the frame of reference and the position of the *N*-th nucleus, respectively.

The paramagnetic contribution, $\sigma_{N;\alpha \beta }^{para}$, is determined through the singlet-excited wave functions, being the linear response function of a paramagnetic nuclear spin–electron orbit interaction (PSO) and orbital Zeeman operator at the static limit. Thus, $\sigma_{N;\alpha \beta }^{para}$ can be represented in the form of the following sum-over-state expression [4]:

(6) $${\overset{⃡}{\sigma }}_{N}^{para}=C^{para}\sum_{n\neq 0}{\left(E_{ns}-E_{0}\right)}^{-1}\left\{\left\langle 0|\sum_{i}{\hat{L}}_{i0}|n_{s}\right\rangle \left\langle n_{s}|\sum_{j}2{\hat{L}}_{jN}^{T}r_{jN}^{-3}|0\right\rangle +h.c.\right\}$$

In Equation (6), the symbols ${\hat{L}}_{iN}$ and ${\hat{L}}_{i0}$ refer to the angular momentum operator relative to the positions of nucleus *N* and the gauge origin, respectively. In all expressions, superscript “T” denotes the vector transposition. The **I**_3_ matrix appearing in Equation (5) is the unit matrix of 3 × 3 that is used to maintain a proper dimension of the ${\overset{⃡}{\sigma }}_{N}^{dia}$ tensor. The summation in Equation (6) runs over index *n*, which indicates the number of excited singlet states |*n*_S_⟩ with the energies *E_ns_*, while indexes *i* and *j* in expressions (5) and (6) run over all electrons of the system. The $E_{0}$ represents the energy of the unperturbed ground state $\left|\left.0\right\rangle \right.$. The $C^{dia}$ and $C^{para}$ in Equations (5) and (6) are the multipliers built of several universal physical constants.

The paramagnetic term ${\overset{⃡}{\sigma }}_{N}^{para}$ reflects the impedance of the electron circulation around the spectator nucleus arising from the presence of the remaining nuclei [4]. It is worth noting that ${\overset{⃡}{\sigma }}_{N}^{para}$ depends on the angular momentum matrix elements, which require the spectator nucleus to have electrons with nonzero orbital angular momentum.

The chemical shielding tensor (4) is commonly referred to as the chemical shift anisotropy (CSA) tensor, which was experimentally established for solids and liquid crystals. The isotropic portion of $\sigma_{N;\alpha \beta }$ represents the NMR shielding constant and makes sense for the liquid- or gas-phase NMR experiment, where the rotation of molecules is so fast that the nuclear shielding tensor, $\sigma_{N;\alpha \beta }$, is averaged so as to give one third of its trace:

(7) $$\sigma_{N;iso}=\frac{1}{3}Tr\left({\overset{⃡}{\sigma }}_{N}\right)$$

Equations (4)–(7) lie in the basis of all quantum chemistry methods developed for NMR shielding constant calculations. Throughout the text, we will imply isotropic shielding constants and chemical shifts, if not stated differently.

In the ^31^P NMR experiment, the so-called chemical shift or *δ*-scale is used to obtain data that are independent of the experimental conditions. The values of *δ*-scale represent the ratio of chemical shifts measured in Hz and the operating frequency of the NMR spectrometer. The dimensionless experimentally measured quantity, called as the NMR chemical shift *δ*, is expressed in points per millionths (ppm) and can be calculated through the shielding tensors of the standard and those of the sample compound. In particular, as in accordance with the IUPAC expression, isotropic chemical shifts can be calculated as follows [5,6]:

(8) $$\delta =\frac{\sigma_{ref}-\sigma_{sample}}{1-\sigma_{ref}\cdot 10^{-6}}\approx \sigma_{ref}-\sigma_{sample}$$

where $\sigma_{ref}$ and $\sigma_{sample}$ represent the nuclear shielding constant of the reference compound or standard and that of a sample compound, respectively; both are measured in ppm.

In the case of the ^31^P chemical shift scale, *σ*(^31^P) is usually converted to *δ*(^31^P) using the 85% aqueous solution of phosphoric acid (H_3_PO_4_) as standard:

(9) $$\delta \approx \sigma_{H_{3}{PO}_{4}}-\sigma_{sample}$$

However, phosphoric acid represents a challenging compound from a computational point view; therefore, it is frequently the case that the secondary standard (ss) is used. In this case, δ is evaluated in accordance with the following equation:

(10) $$\delta \approx \sigma_{SS}-\sigma_{sample}+\delta_{ss/H_{3}{PO}_{4}}$$

In Equation (10), $\sigma_{SS}$ is the ^31^P shielding constant of the chosen secondary standard and $\delta_{ss/H_{3}{PO}_{4}}$ is its experimental chemical shift relative to H_3_PO_4_. The most widely used secondary standard is simple phosphine (PH_3_). Among the first to propose PH_3_ as a secondary standard was van Wüllen [7], who reported the *δ*(^31^P) of PH_3_ relative to H_3_PO_4_ ($\delta_{ss/H_{3}{PO}_{4}}$) to be −266.1 ppm.

Using the linear regression method [8] has already become a well-proven way to convert *σ*(^31^P) to *δ*(^31^P) values, if the corresponding experimental data are known. This method assumes the mapping of the calculated shielding constants, *σ_i_*(^31^P), onto the experimental chemical shifts, *δ_i_*(^31^P), to obtain the linear approximation trend, characterized by two linear parameters α and β, which represent the slope and intercept of the two data sets, respectively. Thus, the scaled chemical shifts, $\delta_{i}^{scaled}$, can be estimated based on the linear regression model as follows:

(11) $$\delta_{i}^{scaled}=\alpha \sigma_{i}+\beta$$

If parameter *α* in Equation (11) equals −1, then parameter β can be thought of as the approximated shielding constant of a standard and, in this case, Equation (11) becomes closely related to the well-known simplified IUPAC expression (8). The linear regression method is usually applied when it is necessary to eliminate systematical errors connected with the improper computational treatment of standard which, in particular, can considerably affect the conclusions about the performance of the different computational methodologies under comparison.

Relativistic representation of NMR shielding constants is not discussed in this section, as this topic goes beyond the boundaries of the present review, though, proper references to the relativistic NMR theory and relativistic quantum chemistry methodology will be provided in Section 7, which is devoted to the relativistic effects on ^31^P NMR shielding constants/chemical shifts.

### 2.2. Gauge Origin Problem in Calculations of NMR Chemical Shifts

One of the problems persisting in the shielding constant calculations is the so-called gauge origin problem, which stems from the dependence of the shielding values on the radius-vector of the center of the coordinate system, as shown in Equations (5) and (6). This makes the final shielding value dependent on the gauge origin. The problem can be solved either by using very large and flexible basis sets [9,10] or by exploiting sophisticated basis sets that introduce individual gauge origins for various local parts of the wave function. The latter constitutes the idea of London’s so-called gauge-invariant atomic orbitals (GIAOs) [11]. Within the GIAO approximation, each atomic orbital centered on nucleus *N* is replaced with the London orbital bearing an explicit dependence on the vector potential at this nucleus. The main purpose of London’s method is to avoid using a single coordinate center, which, in principle, does not provide an entire solution to the calibration problem in the calculations of molecular properties. In other words, notwithstanding the fact that the GIAO approach alleviates the gauge origin problem, it does not provide a localized description of magnetic properties in the full sense. Even more so, for exactly solved quantum mechanical equations, it can be proved that the GIAO is equivalent to a common gauge origin (CGO) formulation of the nuclear magnetic resonance shielding constant, where the gauge origin can be placed anywhere. For example, both approaches were proven to become equivalent for the exact Hartree–Fock (HF) solution in the limit of a complete basis set [12,13], while Schreckenbach [14] showed this equivalence within the framework of the uncoupled density functional theory (uc-DFT) [15,16,17]. Today, the GIAO concept has become a mature theory, which is widely used in the calculations of NMR chemical shifts [13,18,19,20,21].

Kutzelnigg and colleagues also introduced an alternative approach to resolve the gauge origin problem, which is conceptually very close to GIAOs and operates with localized quantities too. The proposed method was called the individual gauge for localized orbitals (IGLOs) [22]. The main difference consists in the fact that, in the IGLO approach, all atomic orbitals (AOs) constituting one molecular orbital (MO) have the same gauge factor, while within the GIAO approach, the gauge factor depends on the AO, so that different AOs within one MO have different phases.

Another approach that effectively alleviates the gauge origin problem in the NMR shielding constant calculations has been presented by Keith and Bader within the framework of a continuous set of gauge transformations (CSGT) [23,24]. In this approach, the current density induced by an external magnetic field is calculated at each point of space, provided that the origin of the coordinate system is placed at the point under consideration. Then, the magnetic properties are calculated using the well-known relations of classical electrodynamics in the form of three-dimensional integrals involving the current density. The continuous transformation of the origin of the current density (CTOCD) [25] method proposes a refinement of the CSGT method’s formalism, which provides analytical expressions to calculate the magnetic properties. The IGLO and CSGT methods have been used in earlier works reporting on ^31^P NMR shielding constant calculations, now we can witness that the IGLO and CSGT methods have faded into the backstage, giving way to the GIAO method.

### 2.3. Brief Overview of the Main Quantum Chemistry Mehods Used in the Calculations of NMR Chemical Shifts

Investigations of the performance of various quantum chemistry methods, as applied to the calculation of ^31^P NMR shielding constants and chemical shifts, have been reported in large number of papers, including detailed comparisons between the Hartree–Fock (HF), density functional theory (DFT), Møller–Plesset (MP), and coupled cluster (CC) theories. Therefore, it is pertinent to briefly mention the basic principles of these theories and their approximations before diving further into the discussion of their application to the calculation of phosphorus NMR chemical shifts.

#### 2.3.1. The Hartree–Fock Approximation

The Hartree–Fock (HF) method [26] represents the simplest approximation that treats a many-electron system as a set of non-interacting “pseudo” one-particle systems. This is achieved by replacing the exact pairwise electron–electron repulsion with an average interaction created by all other electrons. This simplification allows for the many-body problem to be treated as a set of one-electron problems, making the system mathematically separable and easy to solve with the iterative method, namely, the self-consistent field (SCF) technique. The Hartree–Fock model accounts for approximately 99% of a system’s total energy by approximating electron interactions as an average repulsion, but it neglects the electron correlation, which is the instantaneous dynamic interaction between electrons. At the same time, the missing 1% part of the electron energy (correlation energy) is of crucial importance for accurate prediction of NMR parameters; thus, the HF method produces the single-determinant wave function that serves only as the fundamental starting point for almost all advanced electron correlation methods. Given that the HF method per se is for the ground-state wave function only, the calculation of the paramagnetic contributions to NMR shielding tensors (Equation (6)) is carried out within the so-called coupled perturbed Hartree–Fock (CPHF) method. It treats the external magnetic field as a perturbation and solves coupled equations to find the wave function’s response (first derivatives) to that field. The paramagnetic term is derived from the perturbed wave functions. To ensure the gauge origin independence, the CPHF equations were combined with some of the approaches alleviating the gauge origin problem, most importantly, with the GIAO formalism. The implementation of the GIAO approach within the CPHF method for the chemical shift calculations was first carried out by Ditchfield [27], followed by others. Overall, in the earliest stages of the quantum chemistry calculations of *σ*(^31^P)/*δ*(^31^P), the GIAO-CPHF method was the most proliferated approach.

#### 2.3.2. The Density Functional Theory

Density functional theory (DFT) [28,29] represents an essential improvement over the Hartree–Fock theory, as it introduces a fictitious, non-interacting system that has the same ground-state electron density as the real interacting system. The DFT formalism is based on two Hohenberg–Kohn theorems [30,31]. According to the first one, a ground state of the system of interacting particles is a unique functional of the electron density *ρ*(**r**). The consequence of the first theorem is that, for any many-particle system in the ground state, the external potential, *V*_ext_(**r**), determines all the ground-state properties of the system. The second Hohenberg–Kohn theorem establishes a variational principle of quantum mechanics, which states that the electron density that minimizes the energy of the overall energy functional, *E*(*ρ*(**r**)), is the true electron density. *E*(*ρ*(**r**)) includes non-interacting Kohn–Sham kinetic energy, the interaction energy with the external field, the Hartree energy, and the exchange–correlation (XC) energy. The latter can be represented as the sum of errors originating from describing the Hartree and kinetic energy in terms of an idealized, non-interacting system instead of a real interacting system.

If the variational principle is applied to the energy functional (*E*(*ρ*(**r**))), it leads to a system of nonlinear Kohn–Sham (KS) equations, which describe the behavior of the simulated system of non-interacting electrons in some effective local potential. The Kohn–Sham equations have the same structure as the Hartree–Fock equations, with the exchange potential replaced with the local exchange–correlation potential. As a result, the solution of the KS system is akin to the self-consistency procedure in the HF method.

Theoretically, the DFT procedure converges to the exact solution (the exact energy and electron density function of the ground state) and thus could be assigned to ab initio models, if not the exchange–correlation functional, which is unknown. There are many approximations for the exchange–correlation functional, but, generally, they can be divided in four main groups: the local density approximation (LDA) [32,33,34,35], the generalized gradient approximations (GGAs) [36,37,38,39], the hybrid functionals [40,41], and the meta-GGA functionals [42].

The simplest approximation, the LDA, assumes that the energy density is a function of the density only. The LDA approximates the inhomogeneous electron distribution with the homogeneous density distribution, adopting the idea that the electron density changes slowly in space. The GGA functionals depend not only on density, but also on its gradient. In contrast to the LDA, such functionals provide a higher-order approximation. The hybrid functionals incorporate a part of the exact HF exchange. The meta-GGAs depend not only on the electron density and its gradient, but also on its Laplacian.

It is also worth noting that, currently, a substantial increase in accuracy has been achieved due to the continuous efforts to improve the description of the attractive long-range van der Waals interactions, resulting in a number of new DFT functionals (with dispersion correction) addressing the issue [43,44,45,46]. This topic is far beyond the scope of this review. Useful information on modern DFT functionals can be found in recent reviews and feature papers [47,48,49,50,51,52,53,54].

The implementation of the Kohn–Sham theory for the linear response properties such as NMR shielding constants is performed by two conventional approaches, allowing us to calculate the second-order response properties within DFT formalism, namely the finite perturbation theory (FPT) [55] and the response theory (RT). For the NMR calculations, both approaches require an energy expression for a system in the presence of the infinitesimal perturbing magnetic field [56,57]. To provide an optimum way of using these methods in the calculations of NMR chemical shifts, these were extended to include the GIAO or the other gauge origin dependence-eliminating formalisms. In particular, modern applications of the DFT to the calculation of NMR chemical shifts have mostly been developed by Malkin et al. [58,59] in combination with the IGLO [60] formalism, while the GIAO extension to the DFT method was proposed by the group of Schreckenbach and Ziegler [15,16,61,62,63].

Currently, for all gauge-dependent magnetic properties, including NMR shielding tensors, the main problem of DFT is the independence of XC functionals of the paramagnetic current density. The most recent studies by Reimann et al. [64] revealed that the negligence of the current-density contributions can reach substantial values. Based on this idea, Schattenberg and Kaupp, together with their colleagues, have recently published a series of papers presenting the development of local hybrid DFT functionals (LHs) with non-local exchange, which are capable of alleviating the errors coming from the omitted current-density terms. In the first work, they proposed coupled-perturbed (CPDFT) implementation with LHs and GIAO formalism for the NMR chemical shifts [65], while in their subsequent publications, they systematically improved their theory [66,67,68].

It should be noted that NMR chemical shifts in transition-metal complexes, including those with phosphorus-containing ligands, are calculated more accurately with either moderate exact-exchange admixtures or current-density DFT functionals at the meta-GGA level [69]. Moreover, for the transition-metal complexes, it is important to take into account the dispersion corrections [43,70,71], because standard density functionals neglect long-range dispersion interactions and introduce the delocalization error.

As a matter of fact, the DFT method performs considerably better than the HF method and has a comparable formal computational scaling factor with the HF method, ranging from O(*N*^2^) to O(*N*^4^), where *N* is the number of basis set functions. This can be thought of as a great advantage. However, there is also a disadvantage of the DFT method, which consists of the fact that there is no systematic approach to improve the results obtained within this method towards the exact solution. In other words, the quality of the results obtained with the DFT method is quite sensitive to the exchange–correlation functional used. Thus, it is with great caution that one should choose the XC functional for a given problem, because there are already hundreds of them.

#### 2.3.3. The Second-Order Møller–Plesset Approximation

In contrast to the DFT method, the hierarchy of approximations of the many-body Møller–Plesset theory (MBPT) [72] allows us to approach the exact result by systematic improvement of the description of the electron correlation effects within the correlation potential described systematically to a particular order at each hierarchical level of the theory.

For the calculation of NMR parameters, the simplest approximation of the MBPT is usually used, which is called as the second-order Møller–Plesset (MP2) method [73,74]. In earlier stages of NMR chemical shift calculations, the MP2 method was regarded as one of the most attractive methods, since it used to offer a good compromise between the accuracy, computational costs, and systematicity in the electron correlation treatment. Indeed, the MP2 approximation covers the electron correlation energy by ca. 94% and its formal scaling factor is only *N*^5^.

The MP2 expressions for the shielding tensor are derived from differentiating the energy calculated within the MP2 approximation with respect to the nuclear magnetic moment, and then with respect to the magnetic field, which results in the so-called asymmetric second-derivative expression [75]. The resulting expression includes the density matrix in the atomic orbital (AO) basis, its derivatives with respect to the Cartesian components of external magnetic field, and partial first and second derivatives of the elements of the one-electron Hamiltonian in the AO basis with respect to Cartesian components of the nucleus magnetic moment and magnetic field. The main time-consuming operation in the MP2 method is the evaluation of the derivatives of the perturbed density matrix. Actually, this operation stipulates the scaling factor of the method as *N*^5^.

The GIAO formalism has been successfully integrated into the MP2 method [13,76] to be used in NMR chemical shift calculations. Initially, the problem consisted in the calculation of the perturbed two-electron integrals in the GIAO formalism; however, Pulay et al. [76] have shown that such integrals can be calculated in a fairly simple way using the similarity of the perturbed GIAO integrals with the derivatives of ordinary two-electron integrals.

As time wore on, the standards of accuracy for chemical shifts slowly toughened up, thus, making the excellence of the original MP2 method fade away. Yet, this method is still in use in the calculations of ^31^P shielding constants today, though, a great many modifications have been introduced.

In particular, the GIAO-MP2 level has been subsequently extended to the third and fourth orders of perturbation theory [77], based on the MP3 [78] and MP4 [79] theories. All these developments were made using analytic second-derivative techniques. The GIAO-MP3 and GIAO-MP4 methods are substantially more accurate and, of course, are more costly than the GIAO-MP2 approach (*N*^6^ and *N*^7^, respectively, against *N*^5^), but if applied with the approximation of the resolution-of-identity (RI) [80,81], the calculations of ^31^P shielding constants with these models become more feasible.

Another methodological finding that has proven itself useful in the calculations of NMR chemical shifts in many nuclei, including phosphorus, is the infinite-order Möller–Plesset (EMPI) approach [82]. This approach represents a computational method in quantum chemistry that involves a hybrid mixture of HF and MP2 results, tending to balance out these two methods to yield results which are somewhat better than those obtained with the original MP2 method. The EMPI method generally shows superior results for phosphorus chemical shifts over the original MP2 method [83], and this is, in particular, due to the successful treatment of the PN molecule.

#### 2.3.4. The Coupled Cluster Formalism

The methods of the coupled-cluster (CC) formalism [84,85,86,87,88,89,90,91,92,93,94] are size-consistent and have reasonable computational costs. Nowadays, the CC method represents one of the most accurate and reliable correlated ab initio approaches for the calculation of NMR chemical shifts. The CC wave function is obtained from the HF ground-state wave function by applying the exponential excitation operator to it; thus, the CC wave function can also be expressed as an infinite series of the excited determinants [95,96].

The exponential excitation operator consists of the sum of the operators of different excitation classes, including single, double, and triple, etc., entering the overall excitation operator through the coupled cluster amplitudes. The cluster amplitudes are found from the system of equations, which is constructed by multiplying the Schrödinger equation from the left by the excited configurations of different classes. These equations are solved iteratively until the desired accuracy is reached. Once the cluster amplitudes are determined, one obtains the ground-state wave function and can calculate the ground-state energy accordingly.

To calculate the second-order magnetic molecular properties, such as the paramagnetic part of the NMR shielding constants, the summation over the excited states within the framework of a linear response function is required. In this respect, either the coupled-cluster linear response (CCLR) [84,85,86,87] or equation-of-motion coupled clusters (EOM-CC) [97,98] are employed.

Reducing excitation operators down to a single excitation class provides the simplest approximation of the CC theory, the so-called coupled-cluster singles (CCS) scheme. This scheme is not equivalent to the HF method; it is slightly superior to it. Within the CCS approximation, the excitation energies are calculated correctly up to the first order in the electron correlation interaction. This approximation is rarely used in the calculations of NMR chemical shifts. The next approximation is the sequential expansion of the excitation operator up to the class of double excitations. This makes the so-called coupled-cluster singles and doubles (CCSD) approximation [99]. This is the most widely used approximation among all CC schemes in the calculations of NMR chemical shifts. This is a very accurate scheme that takes into account ca. 98.3% of electron correlation. For most cases it appears to be enough, though, there are some molecules (mostly containing triple bonds) manifesting such strong electron correlation effects that even this level is not sufficient; therefore, the inclusion of higher excitation classes is needed. Thus, the next levels are the coupled-cluster singles, doubles, and triples (CCSDT) [100,101] and coupled-cluster singles, doubles, triples, and quadruples (CCSDTQ) [102,103,104]. The CCSDT and CCSDTQ cover the electron correlation energy by ca. 99.7 and 99.9%, respectively. The higher hierarchy CC schemes rapidly become prohibitive, even for the small molecules, since the formal computational scaling factor for the whole series of schemes, CCS, CCSD, CCSDT, and CCSDTQ, increases as follows: *N*^4^, *N*^6^, *N*^8^, and *N*^9^, where *N* is the number of basis set functions [90]. In this respect, there are a number of approximations to pure CC schemes that are widely used in the NMR calculations due to their lowered computational requirements relative to pure CC schemes.

One of the most popular approximate schemes is the CC2 [105]. Within the CC2 model, the equations for the amplitudes for single excitations are the same as in the CCSD method, but the equations for the amplitudes for double excitations are approximated so that they are accurate only up to the first order, according to the perturbation theory in the fluctuation potential. Thus, the CC2 model is intermediate between the CCS and CCSD models. The computational cost of the CC2 model can be expressed as *N*^5^. Another approximate model, which was built based on a similar concept, is the CC3 model [106]. The CC3 model represents an intermediate model between the CCSD and CCSDT schemes, so that the computational scalability of CC3 is *N*^7^.

Another approximation of CC theory that captures the effects of triple excitations at the lowest possible computational cost is the coupled-cluster singles and doubles with non-iterative perturbative triples (CCSD(T)) [107]. In this scheme, the triples amplitudes are estimated from the triple excitation equations, as they occur in the lowest non-vanishing order in the Möller–Plesset perturbational theory. The formal scaling factor of the CCSD(T) scheme is the same as that of the CC3 model, namely, *N*^7^. Overall, the CCSD(T) model has proven successful in the calculations of NMR chemical shifts and has received great popularity in the calculations of *σ*(^31^P)/*δ*(^31^P) in particular.

The introduction of the GIAO formalism into the coupled-cluster theory was originally presented by Gauss et al. [108,109] at the CCSD level. The implantation of the GIAO formalism to the CCSD(T) and CCSDT models was proposed by Gauss and Stanton with colleagues [91,110,111].

## 3. Quantum Chemical Calculations of ^31^P NMR Chemical Shifts Using Conventional Approaches and Nonspecialized Tools

Some thirty years ago, our knowledge about the efficiency and accuracy of different quantum chemistry methods and basis sets for ^31^P NMR chemical shift calculations was scarce and scattered. In this respect, the authors of pioneering works clearly realized the importance of the proper choice of methods and basis sets, because it is these aspects that would build the foundation for further development of the computational methodology for the ^31^P NMR spectra simulation.

Pioneering works on ^31^P NMR chemical shift calculations appeared in the literature as soon as various methods of solving the gauge origin problem were implemented into widely available quantum chemistry codes. Early calculations of ^31^P NMR chemical shifts were mainly carried out within the HF, DFT, and MP2 methods, applied in combination with the GIAO, IGLO, and CSGT formalisms. In the majority of cases, nonspecialized energy-optimized basis sets of various types, namely Pople’s *K-LM* [112,113,114], *K-LMN* [115], Kutzelnigg’s IGLO-*n* (*n* = II, III) [22], and Dunning’s cc-pVXZ (X = D, T, Q, 5) [116,117], with or without the augmentation with diffuse or polarization functions, were used.

In particular, Gauss [118], Rezaei-Sameti et al. [119], and Tafazzoli et al. [120] investigated the performance of the HF, DFT, and MP2 methods for *δ*(^31^P) on the example of compounds of the general structure PXYZ, with X, Y, and Z being hydrogen or simple alkyl substituents. Solvent and vibrational effects were not taken into account in these works. In general, it was found that the HF and MP2 methods considerably underestimate and slightly overestimate, respectively, the result relative to experiment, while the DFT method, if applied with certain DFT functionals, provides much better accuracy than the HF method and even is capable of surpassing the MP2 method, regardless of the approach used for eliminating the gauge origin problem.

In the mentioned works, Pople’s double- and triple-zeta quality basis sets, the 6-31G and 6-311G, with or without additional sets of diffuse and polarization functions, were used. It was found that an acceptable level of accuracy for all three methods can be reached only with the triple-zeta quality basis sets augmented with additional polarization functions, the 6-311G(d,p) or 6-311G(2d,2p). Lowering the cardinal number of the basis set used (i.e., going to the double-zeta level) or the exclusion of additional polarization functions immediately results in increasing the mean absolute deviation of the theoretical values from experiment up to two times. The inclusion of the additional diffuse functions turned out to have a negligible effect on the accuracy of the calculated phosphorus chemical shifts.

In all three works [118,119,120], the HF, DFT, and MP2 methods were established to provide the mean absolute error (MAE) for *δ*(^31^P) against the experiment of about 10–15 ppm, regardless of the peculiarities of the calculation. In this respect, it is worth mentioning that the best theoretical results achieved by Tafazzoli et al. [120] within the HF, DFT(B3LYP) [38], DFT(PBE) [121,122], and MP2 methods, applied in combination with the GIAO formalism and the 6-311G(d,p) basis set, can be characterized by the MAEs of 14, 12, 10, and 13 ppm, respectively. Therefore, Tafazzoli et al. recommended the DFT(PBE)/6-311G(d,p) computational scheme to be the best suitable for the calculation of *δ*(^31^P).

van Wüllen [7] and Maryasin et al. [123] have also considered the performance of the DFT and MP2 approaches used in the combination with either the GIAO or IGLO approaches and the 6-311++G(2d,2p) and IGLO-II (or IGLO-III for special cases) basis sets for the calculations of *δ*(^31^P), though, on a wider series of phosphorus compounds than that which was mentioned above. This included PX_3_ (X = H, F, Cl, CH_3_, ^i^C_3_H_7_, OCH_3_, Ph_3_), OPX_3_ (X = CH_3_, OCH_3_, Ph_3_), Si(PH_2_)_4_, Cr(CO_5_)(PH_3_), PX_4_^+^ (X = H, CH_3_), PF_6_^−^, P_4_, PN, and some other complex systems like tetrahydro-[1,3,2]oxazaphospholo [2,3-b][1,3,2]oxazaphosphole and N-(2,4-dimethylpentan-3-yl)-N’,N’-diisopropyl-1-vinylphosphanediamine. These set of molecules has provided a great challenge for all computational techniques under study, as it includes molecules with extremely complicated electronic structures, such as PN and Cr(CO_5_)(PH_3_), or charged molecules like PH_4_^+^ and PF_6_^−^. In general, the GIAO-MP2 method in combination with the 6-311++G(2d,2p) or IGLO-II basis sets provided the best accuracy with an MAE against the experiment of approx. 16–17 ppm. However, this MAE was calculated without taking into account the *δ*(^31^P) of Cr(CO_5_)(PH_3_) and PN molecules, as the GIAO-MP2 results for these were not good, because the MP2 method is known for its poor performance for systems bearing a substantial amount of electron correlation [124].

Maryasin et al. [123] suggested that although the MP2 method appears to be more reliable than the DFT method, the predictions at the MP2 level are significantly more expensive and a DFT-based protocol of comparable accuracy would be desirable. The best DFT-based computational schemes proposed by Maryasin et al. and van Wüllen are the GIAO-DFT(MPW1K [125])/6-311++G(2d,2p) and GIAO-DFT(B3LYP)/IGLO-II, respectively. They have provided the MAEs of 17.2 and 16.2 ppm, accordingly. 

The idea of Maryasin et al. about finding the most accurate combination of the XC functional and basis set for *δ*(^31^P) has received great popularity; many researchers have invested considerable effort in this issue.

Thus, a comprehensive study of the performance of the HF and various DFT models used in combination with different basis sets has been performed by Chernyshev and Krivdin [126], using the example of the simplest phosphines, phosphine oxides, and phosphine sulfides with methyl, ethyl, and methoxy groups as substituents. The calculations were performed within the GAIO-HF and GIAO-DFT methods, with the latter being applied in combination with the B3LYP, KT2 [127,128], KT3 [129], and PBE0 [122,130,131] functionals. In order to estimate the basis set effect on the accuracy of the calculated *δ*(^31^P), the authors exploited the cc-pVDZ, 6-311G(d,p), cc-pVTZ, IGLO-II, and IGLO-III basis sets, with the quality of the basis sets increasing from left to right.

The GIAO-HF approximation gave significant deviations of the calculated *δ*(^31^P) from those found experimentally, with MAEs from 40 to 80 ppm, depending on the basis set used. The DFT method with the KT3 functional provided an improvement over the GIAO-HF results, lowering the MAE by 15–20 ppm for all basis sets. The KT2 gave further improvement, reducing the MAE by another 10–15 ppm as compared to the KT3 results. The best results were obtained with the B3LYP and PBE0 functionals, for which the MAE was found to vary in the range from ca. 48–50 ppm for the cc-pVDZ basis set to ca. 20–25 ppm for the 6-311G(d,p) basis set to ca. 9–12 ppm for the IGLO-III basis set. The authors recommended the GIAO-DFT(B3LYP)/IGLO-III computational scheme as the best procedure for the calculation of *δ*(^31^P) in the series of phosphines, phosphine oxides, and phosphine sulfides, as it provided the MAE of only 9 ppm and quadratic Pearson coefficient of ca. 0.9599 against the experiment.

A few years later, Fedorov et al. [132] also presented a study of various DFT protocols for phosphorus chemical shift calculations. The authors used only one molecule for their testing, namely, phosphine, PH_3_. For that moment, the best achieved value of *σ*(^31^P) of PH_3_ was 606.11 ppm, which had been calculated by Lantto et al. [133] at the GIAO-CCSD(T)/cc-pwCV5Z level of theory; thus, Fedorov et al. used this value as the reference. They chose essentially the same DFT functionals and basis sets as were considered in the work of Chernyshev and Krivdin [126], including the B3LYP, B3PW91 [36,134], LDA, PBE0, KT2, and KT3 functionals and various series of basis sets, namely, that of Pople (from 6-31+G(d) to 6-311++G(3d,2p)), Jensen ((aug-)pcS-*n*, *n* = 1–4) [135], and Dunning ((aug-)cc-pVXZ, X = T, Q, 5, 6).

It is embarrassing to note that the conclusions reached by Fedorov et al. for PH_3_ are in controversy with those deduced by Chernyshev and Krivdin for a series of phosphines, phosphine oxides, and phosphine sulfides. According to Fedorov et al., the best functionals for the calculations of *σ*(^31^P) turned out to be KT2 and KT3, while these were anticipated to be the worst ones by Chernyshev and Krivdin. At the same time, the B3LYP functional showed the worst and the best performance simultaneously in all calculations conducted by Fedorov et al. and Chernyshev and Krivdin, respectively.

Moreover, the use of the KT2 functional, chosen as the best-suited one by Fedorov et al., resulted in an inconsistency in the results obtained within each considered hierarchy of basis sets, i.e., the average absolute errors increased twice or even thrice in going from 6-31G(d) to 6-311+G(d), from cc-pVTZ to cc-pVQZ, and from pcS-1 to pcS-2. This is not the usual behavior upon improving the basis set quality. Perhaps, making conclusions about the performance of the DFT models based on the results for one particular molecule (PH_3_) might have been a mistake.

**Figure 1:**
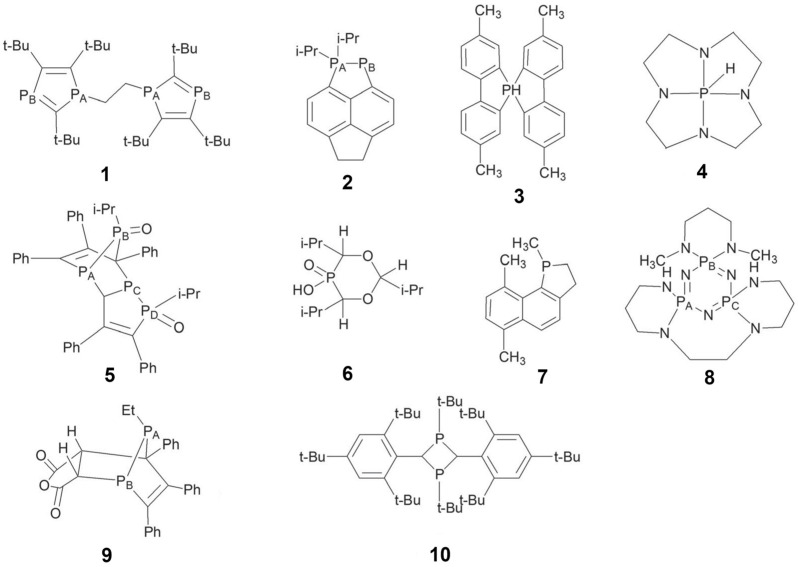
Structures of real-life compounds **1**–**10** used in the testing of PBE0/6-311G(2d,2p)//PBE0/6-31+G(d) computational scheme against experiment. Reproduced from Latypov et al. [136] with the permission of the Royal Society of Chemistry.

Latypov et al. [136] tested a variety of methods, including the HF, DFT(B3LYP), DFT(PBE0), and MP2, in combination with Pople’s basis sets of different quality, namely, the 6-31G(d), 6-31+G(d), 6-31G(2d), 6-31G(d,p), 6-31+G(d,p), 6-311G(d), 6-311G(2d,2p), 6-311++G(d,p), 6-311++G(2d,2p), and 6-311++G(3df,3pd). Thirty-four small phosphorus compounds, with phosphorus being in a diverse bonding situation, including phosphines, phosphine oxides and phosphine sulfides with alkyl substituents, as well as some very complicated (from the electronic structure point of view) molecules, such as PCl_6_, PF_6_, P_4_, PN, P_2_H_2_, and PCF, were considered. Latypov et al. chose the B3LYP and PBE0 functionals for testing, which agrees with Chernyshev and Krivdin’s results [126], indicating the popularity of these two functionals in ^31^P chemical shift calculations. The authors carried out a two-stage test, with the first stage resolving the issue of the geometry factor effect (the influence of the method/basis set used in the geometry optimization) and the second stage intended to search for the best suitable method/basis set combination for ^31^P chemical shift calculations. It was found that the level of theory and the basis set used in the geometry optimization stage are not as important as those used in the NMR shielding calculation stage. As a result, the PBE0/6-31G(d)//PBE0/6-31G(d) and PBE0/6-31G(2d)//PBE0/6-31G(d) combinations were recommended for express and routine calculations of *δ*(^31^P). At the same time, the PBE0/6-311G(2d,2p)//PBE0/6-31+G(d) level has been proposed to obtain a better result at a reasonable computational cost. The latter scheme has been approbated on the example of the sufficiently large real-life compounds shown in Figure 1.

The phosphorus shielding constants of compounds **1**–**10** were calculated with the PBE0/6-311G(2d,2p)//PBE0/6-31+G(d) computational scheme, taking into account the solvent effects within the polarizable continuum model (PCM) [137]. NMR shielding constants were transformed into chemical shifts in accordance with the simplified IUPAC expression for NMR chemical shifts, using PH_3_ as the secondary standard (see Equation (10)). For the *δ*(^31^P) of molecules **1**–**10**, the MAE provided by the PBE0/6-311G(2d,2p)//PBE0/6-31+G(d) scheme against experiment can be evaluated to be approx. 12.4 ppm.

**Figure 2:**
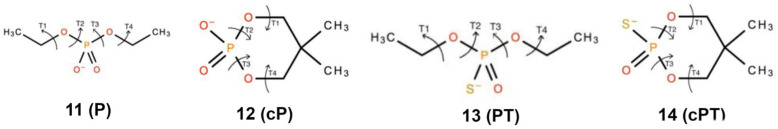
Structures of real-life compounds **11**–**14** considered by Fukal et al. [138,139]. Reproduced from Fukal et al. [138,139] with permission from the Royal Society of Chemistry.

Fukal et al. [138,139] has presented an interesting experimental and theoretical ^31^P NMR study of structurally constrained and flexible phosphates, namely, diethylphosphate (**11**, P), 5,5-dimethyl-2-hydroxy-1,3,2-dioxaphosphinane 2-oxide (**12**, cP), O,O-Diethyl thiophosphate (**13**, PT), and 5,5-dimethyl-2-mercapto-1,3,2-dioxaphosphorinane 2-oxide (**14**, cPT). These are schematically depicted in Figure 2.

**Figure 3:**
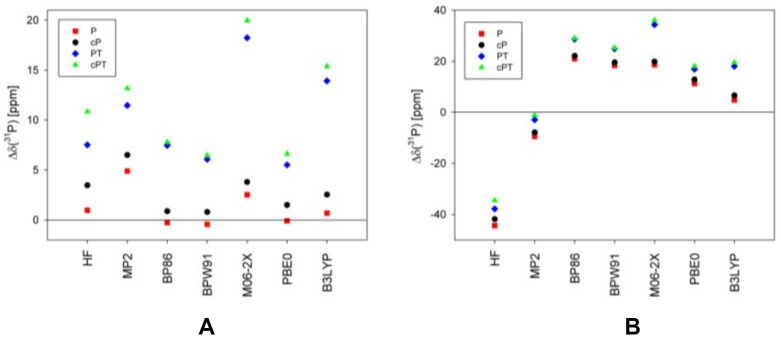
The differences in calculated ^31^P NMR chemical shifts in compounds **11** (P), **12** (cP), **13** (PT), and **14** (cPT) with the experiment: (**A**) *δ*(^31^P) referenced to H_3_PO_4_; (**B**) *δ*(^31^P) referenced to PH_3_ used as the secondary standard. Reproduced from Fukal et al. [139] with the permission of the Royal Society of Chemistry.

Phosphates and phosphate diesters **11**–**14** are important biologically active compounds that are involved in formation of nucleic acid chains, including DNA and RNA. At the same time, thiophosphate diesters are synthetic analogs of the natural phosphodiester backbone in RNA and DNA, frequently used when studying the mechanisms of various biochemical processes. By replacing a non-bridging oxygen with sulfur, they alter the chemical and physical properties of the nucleic acid, allowing researchers to investigate enzyme function, splicing mechanisms, and nucleic acid–protein interactions. Moreover, the ^31^P NMR shift is known to strongly depend on the geometry of the phosphodiester group [140]. Using the example of compounds **11**–**14**, Fukal et al. investigated the performance of various computational methods against experiments for phosphorus NMR calculations, including the HF, MP2, and several DFT methods, without taking into account explicit solvent and relativistic effects. The performance of different methods is demonstrated for compounds **11** (P), **12** (cP), **13** (PT), and **14** (cPT) in Figure 3.

As can be seen from Figure 3, the ^31^P NMR chemical shifts in thiophosphates deviate from the experiment to a greater extent compared to phosphates, except for the *δ*(^31^P) relative to PH_3_ calculated within the HF and MP2 methods, indicating a systematical unaccounted computational error of *δ*(^31^P) in thiophosphates relative to that of phosphates. According to these results, the most accurate results were obtained within the GIAO-DFT(B3LYP) computational protocol. 

At the same time, the study of the performance of various one-electron basis sets has been conducted for *σ*(^31^P) on the example of molecules **11** (P) and **12** (cP). The authors considered the IGLO-*n* (*n* = II, III), cc-pVXZ (X = D, T, Q, 5), and pcS-*n* (*n* = 0, 1, 2, 3, 4) basis sets. For that purpose, they have chosen the GIAO-DFT(B3LYP) method in combination with the PCM water-parametrized solvent model. The pcS-4 represented the reference basis set in this work.

**Figure 4:**
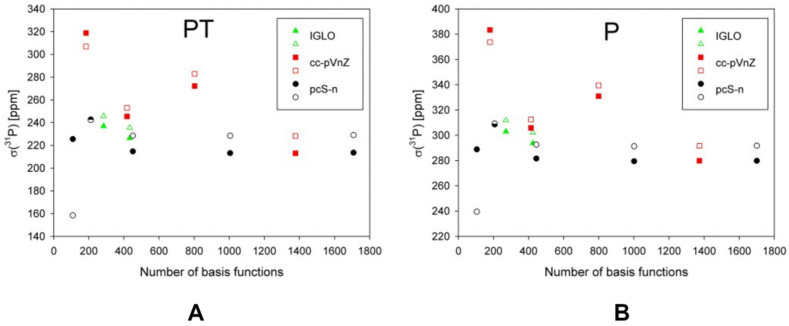
The dependences of *σ*(^31^P) NMR shielding constants of compounds PT (plot (**A**)) and P (plot (**B**)) calculated within the GIAO-DFT(B3LYP) method (with PCM water-parametrized solvent model) on the number of basis set functions contained in the used atomic basis sets, namely, the IGLO-*n* (*n* = II, III), cc-pVXZ (X = D, T, Q and 5), and pcS-*n* (*n* = 0, 1, 2, 3, 4). Reproduced from Fukal et al. [139] with the permission of the Royal Society of Chemistry.

As can be seen from Figure 4, the usual nonspecialized energy-optimized series of Dunning’s basis sets, the cc-pVXZ, provided smooth but very slow convergence, while the other two basis set series performed considerably better.

The most recent studies on the DFT-based computational protocols for *δ*(^31^P) have been presented by Hersh et al. [141] and Gao et al. [142]. The main purpose of that work consisted in developing a high-accuracy method that would both allow the efficient identification of unusual phosphorus compounds and be accessible in standard available software. The authors of papers [141,142] applied the GIAO-DFT method with different XC functionals to the calculation of *σ*(^31^P) in tri- and tetracoordinate phosphorus compounds, partially coinciding with the training test sets used by Latypov et al. [136]. In both works, the linear regression technique (see Equation (11)) was applied to evaluate scaling factors for the calculation of *δ*(^31^P). As a result, in both works, the best DFT scheme provided the root-mean-square deviation (RMSD) of approx. 5.5–5.7 ppm. In particular, Hersh et al. proposed the M06-2X/6-311+G(2d,p)//M06-2X/6-31+G(d,p) as the best scheme. In general, the DFT protocols combined with the linear regression techniques for chemical shift evaluations, which are capable of providing an RMSD of the theoretical values vs. experiment of approx. 5–6 ppm, can be thought of as sufficiently accurate; however, it should be noted that the typical accuracy of the scaled theoretical chemical shifts compared to that calculated in accordance with the classical IUPAC (Formula (9) or (10)) is usually better due to the alleviation of the systematical errors. Evidently, in the actual predictions of *δ*(^31^P) in phosphorus compounds with diverse electronic structures, there is no guarantee that the devised DFT protocols with determined scaling parameters for the linear regression model will result in theoretical values of *δ*(^31^P) with the same RMSD of 5–6 ppm against the experiment.

Recent progress in computer technology made it possible to conduct highly accurate ab initio calculations of ^31^P NMR chemical shifts, producing results that allow for unambiguous comparison with experiments. Actually, in the majority of cases, such calculations imply resorting to high-hierarchy models of ab initio approaches that treat the electron correlation effects in a systematical way, such as within the methods of coupled-cluster (CC) theory. In this respect, CCSD or CCSD(T) represent highly accurate schemes, with the latter even regarded as the “gold standard”, which serves as a reference for many lower-level computational schemes. Although the computational cost of these methods is relatively high and their application was originally feasible only for small molecules, nowadays they are routinely used for middle-sized systems due to the development of specific algorithms allowing effective computations on parallel architectures [143,144,145,146,147]. Saliant paragons of works that present high-quality CC calculations of *σ*(^31^P)/*δ*(^31^P) are not many in number, though, their worth can hardly be overestimated.

Ab initio calculations of *σ*(^31^P) of three diatomic molecules, PN, P_2_, and AsP, were performed by Antušek et al. [148]. These molecules have strong covalent triple bonds; therefore, they represent a challenging and austere test for any computational methodology, because the quality of molecular properties computed for these molecules strongly depends on the treatment of electron correlation effects. The authors conducted the calculations of *σ*(^31^P) at the CCSD and CCSD(T) levels in combination with the cc-pVXZ, cc-pCVXZ, and cc-pVXZ(s,p-unc) (X = T, Q, 5) basis sets, although they did not compare theoretical results with experiments.

The CCSD and CCSD(T) calculations for molecules PN, P_2_, and AsP showed that accounting for the electron correlation effects is extraordinarily important for these molecules, as they provide a change in *σ*(^31^P) of more than 100 ppm when going from uncorrelated HF to correlated CC schemes. At the same time, considering the non-iterative triple excitations within the CCSD(T) model provides an additional contribution with respect to the CCSD model of about 20–30 ppm for PN and P_2_ and of about 50 ppm for AsP. The best CCSD(T)/cc-pV5Z(s,p-unc) values for *σ*(^31^P) of molecules PN, P_2_, and AsP calculated by Antušek et al. are 64.87, −191.77, and 193.99 ppm, respectively.

The series of considered basis sets have their advantages and drawbacks. In particular, the cc-pVXZ basis sets are not optimal for the calculations of NMR properties. Among their drawbacks is the slow and often non-monotonous convergence of computed molecular properties with cardinal number X. Apparently, the authors considered them only for comparative reasons, showing the least suitable alternative. The cc-pVXZ(s,p-unc) basis sets contain the *s*- and *p*-functions in an un-contracted form and represent the more interesting choice, because increasing the quality of the description of the *p*-shell by various methods, e.g., by means of lessening the contraction scheme or by expanding the *p*-shell with additional functions, usually improves the basis set convergence for the nuclear magnetic shielding constants toward complete basis set (CBS) limit [135]. The cc-pCVXZ basis set contains additional core–valence correlation functions, which provide rapid exponential convergence of core, core–valence, and valence correlation energies calculated within ab initio correlated approaches towards the CBS limit [149]. These can possibly improve the quality of the NMR chemical shift calculations due to more qualitative treatment of the electron correlation effects as a whole.

The basis set study carried out by Antušek et al. for molecules PN, P_2_, and AsP revealed that within all considered levels of electron theory, the standard cc-pVXZ basis sets provide non-monotonous behavior of *σ*(^31^P), with an evident outlier for the cc-pVQZ case, which indicates that the basis sets of this type, if applied in contracted form, are not sufficiently flexible to compute accurate shielding values. At the same time, the convergence of the cc-pCVXZ series was much smoother compared to that of the cc-pVXZ series, resulting in essentially the same basis set limit at the pentuple-zeta level as that provided by the cc-pVXZ series. Meanwhile, the cc-pVXZ(s,p-unc) gave similar convergence patterns to that obtained with the cc-pCVXZ basis set, providing practically the same quality of calculated *σ*(^31^P) with that achieved when using the cc-pCVXZ basis set with equal cardinal number X. However, it should be noted that, for each cardinal number X, the cc-pVXZ(s,p-unc) basis set contains much smaller number of basis set functions than the cc-pCVXZ basis set does.

Prochnow and Auer [150] have reported on high-quality ab initio benchmark calculations of *σ*(^31^P) for a representative set of phosphorus molecules, including PN, PH_3_, PF_3_, P_4_, and P(CH_3_)_3_. For the calculation of *σ*(^31^P), the authors used the HF, CCSD, and CCSD(T) methods, and, in addition, the MP2 and the DFT method with the BP86 [36,37] and B3LYP functionals. They monitored the convergence of *σ*(^31^P) over the used basis sets by performing the CCSD(T) calculations. The *s*- and *p*-functions of the used basis sets were taken from the TURBOMOLE program package [151] and the polarization functions, added in various numbers, were taken from the paper of Woon and Dunning [116], so that the considered basis sets were labeled as the tz2d, qz2d, qz3d1f, and uncontracted (15s12p4d3f2g), assuming the triple- and quadruple-zeta quality for the former and the rest of basis sets, respectively. A gradual improvement of the basis set quality applied in the CCSD(T) calculations, namely, in going from tz2d to (15s12p4d3f2g), resulted in a reduction of the values of *σ*(^31^P) by 30% for PN and by 1–4% for the rest of the molecules. It is interesting to note that the most essential drop was observed in going from qz2d to qz3d1f for all molecules (e.g., it was about 15 ppm for PN), which definitely indicates an exceptional sensitivity of the calculated *σ*(^31^P) to the quality of the description of the first and even the second polarization shells when highly correlated ab initio methods such as the CCSD(T) method are in use.

In addition, based on the results of Prochnow and Auer, one can conclude that *σ*(^31^P) can be strongly dependent on the quality of the description of electron correlation effects in some cases and be practically irreverent to it in other ones. For example, the difference between the CCSD(T) and HF values of *σ*(^31^P) in the PN and P(CH_3_)_3_ molecules is about 170 and 1 ppm, respectively. In this respect, Antušek et al. [148] and Teale et al. [152] also carried out the HF, CCSD, and CCSD(T) calculations of *σ*(^31^P) in PN molecule using the quadruple-zeta level basis sets. Their results indicated that Prochnow and Auer [150] were correct about huge correlation effect on the *σ*(^31^P) of the PN molecule. The differences between the HF and CCSD(T) values were estimated as ca. 140 by Antušek et al. [148] and as ca. 165 ppm by Teale et al. [152]. Even the role of triple excitations in the PN molecule occurred to be extremely significant, because the difference between the CCSD(T) and CCSD values is about 20–25 ppm [148,150,152], while the total theoretical CCSD(T) value of the phosphorus shielding constant of PN is 50–60 ppm [148,150,152,153], depending on the basis set used.

In general, for PN molecule and the other phosphorus-containing molecules exhibiting strong electron correlation effects, the GIAO-CCSD(T) level applied with the basis sets of quintuple- or sextuple-zeta levels, taking into account the rovibrational and relativistic corrections (if needed), is capable of providing theoretical values of *δ*(^31^P) deviating from the gas-phase NMR experiment by no more than 1–2 ppm [154].

**Figure 5:**
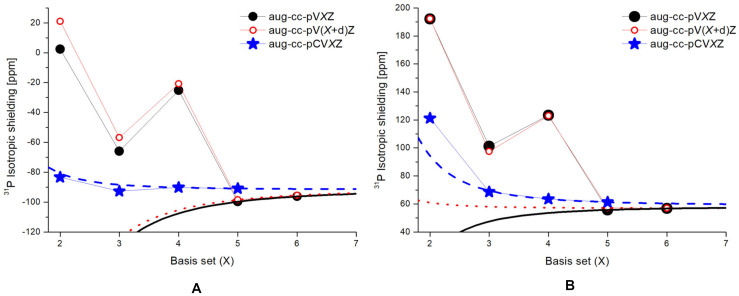
Convergence of ^31^P isotropic shielding of PN with increasing the quality of Dunning’s basis sets used in the HF-SCF (**A**) and the CCSD(T) (**B**) calculations. Reproduced from Kupka et al. [155] with the permission of John Wiley & Sons, Inc. (Hoboken, NJ, USA).

Another comprehensive high-quality study of the *σ*(^31^P) of PN molecules and the other small highly symmetric molecules was presented recently by prof. Kupka and his colleagues [155,156], who are deeply involved in the theory and computations of NMR parameters [157]. The work [155] solely considered phosphorus mononitride molecule. The authors performed a thorough benchmark study of structural, electronic, and spectral IR and NMR properties of PN molecule at the HF, DFT, CCSD, and CCSD(T) levels of theory and observed gigantic oscillations for the calculated *σ*(^31^P) within the series of correlation-consistent basis sets aug-cc-pVXZ and aug-cc-pV(X+d)Z, with X varying from D to six. These oscillations are shown in Figure 5, with the left and right graphs corresponding to the HF and CCSD(T) levels of theory.

Apparently, the significant outlier at the quadruple-zeta level of the aug-cc-pVXZ series, observed by Kupka et al. for the PN molecule (as shown in Figure 5), represents the same computational artifact that was found earlier by Antušek [148] for PN, P_2_, and AsP molecules when performing the calculations based on the cc-pVXZ basis set. An extension of the phosphorus basis set with one tight *d*-function (the aug-cc-pV(X+d)Z series) improved the convergence neither at the HF-SCF nor at the CCSD(T) levels of theory. Therefore, Kupka et al. suggested that the oscillations of *σ*(^31^P) cannot be explained solely by the correlation effects that should be described better within the CCSD(T) method when the expansion of the *d*-shell of the basis sets is performed. They suggested that the cc-pVXZ, aug-cc-pVXZ, and aug-cc-pV(X+d)Z series of basis sets are just not suitable for accurate calculations of phosphorus shielding constants. At the same time, Kupka et al. has found that using the core–valence basis set (aug-cc-pCVXZ) changed the convergence pattern of the *σ*(^31^P) of PN towards a smooth trend, providing the converged value already at the triple- and quadruple-zeta levels of the aug-cc-pCVXZ series within the HF and CCSD(T) methods, respectively. The same change was observed by Antušek et al. [148] when switching from the cc-pVXZ to the cc-pCVXZ series.

In the next paper, Kupka et al. [156] reported on the importance of proper basis set selection to obtain accurate and reliable NMR shielding parameters for nuclei of the third period, including phosphorus. The *σ*(^31^P) of PH_3_, H_3_PO, and PN was calculated using the SCF-HF, DFT-B3LYP, and CCSD(T) methods, combined with the Dunning’s aug-cc-pVXZ, core–valence aug-cc-pCVXZ (X = D, T, Q, 5, and 6), Jensen’s polarized aug-pcSseg-*n* (*n* = 0–4) [158], and Karlsruhe x2c-XPall-s (X = SV, TZVP, QZVP) basis set families [159].

**Figure 6:**
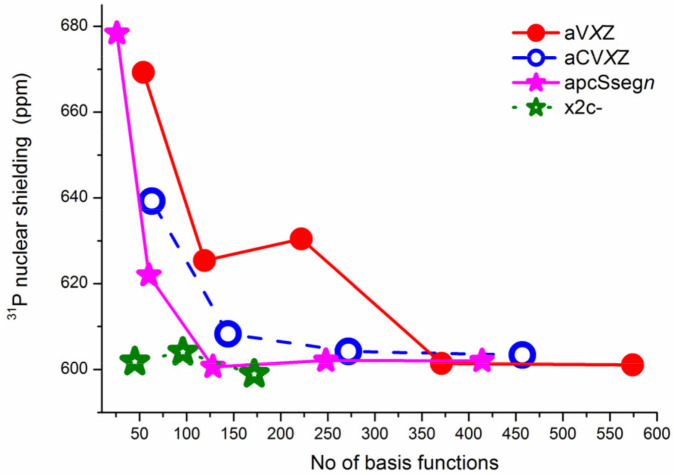
Convergence of ^31^P isotropic shielding constants for PH_3_ vs. the number of basis set functions, calculated with the CCSD(T) method combined with the aug-cc-pVXZ, aug-cc-pCVXZ, aug-pcSseg-*n*, and x2c-XZVPall-s basis set families. Reproduced from Kupka et al. [156] with the permission of MDPI (Basel, Switzerland).

All series of the considered basis sets, except for aug-cc-pVXZ, yielded a regular and smoothly convergent trend towards the CBS limit. For illustration, the convergence trends for the *σ*(^31^P) of PH_3_ calculated at the CCSD(T) level of theory with different types of basis sets are shown in Figure 6. The trends calculated within the HF and DFT approaches proved to be essentially the same as those obtained within the CCSD(T) method, differing only by the range in which the values varied.

As can be seen from Figure 6, the trends for PH_3_ molecule calculated for the aug-cc-pVXZ and aug-cc-pCVXZ basis sets seem to be closely reminiscent of the trends obtained by Kupka et al. [155] for the case of PN molecule, see Figure 5. The same can be found for the H_3_PO molecule. Thus, the usual (aug-)cc-pVXZ basis set series generally results in the poor, fluctuating convergence of *σ*(^31^P) with significant outliers at the quadruple-zeta level, no matter which molecule is under consideration. At that, including the functions that improve the description of the core–valence correlation effects, like in the aug-cc-pCVXZ basis set, results in the alleviation of the problem and proves to be important for the accurate calculation of *σ*(^31^P). The other two series of basis sets demonstrated very fast convergence, and this is not surprising, because both of them represent advanced basis sets, developed specifically for the NMR shielding constant calculations. They will be discussed later in this review.

As compared to the gas-phase experiment, the errors of the theoretical CBS limits for the values of *σ*(^31^P) calculated by Kupka et al. within the CCSD(T) method, taking into account vibrational corrections, is approx. 12–17 ppm, depending on the molecule.

Resuming this section, it is worth reminding that the computational works surveyed in this section reported on the conventional application of the well-known quantum chemistry methods that were used mainly in combination with standard energy-optimized nonspecialized basis sets. These works greatly contributed to our knowledge about efficiency of various conceptually different quantum chemistry methods in the calculations of *σ*(^31^P)/*δ*(^31^P) and helped to establish the levels of accuracy typical for each method. Therefore, these works served as the ground for further development of the computational methodology, aimed at reducing computational costs and improving the accuracy of the results. The main findings of these works are briefly mentioned below.

The HF method provides very low quality of the results which does not comply with modern standards of accuracy. The DFT protocol provides a significant improvement over the HF method due to the inclusion of electron correlation treatment through the XC functional at the same or lower computational cost as that of the HF method. On average, an absolute error of the calculated *δ*(^31^P) against the experiment that can be expected from the DFT method varies in the range of 10–20 ppm, depending on the XC functional and basis set used. At the same time, modern scaling techniques are capable of providing mean errors of 5–7 ppm, however, they are applicable only if the experimental values are known a priori and can be strictly assigned to corresponding theoretical data.

The ab initio methods with systematic accounting for the electron correlation effects, such as MP2, CCSD, and CCSD(T), propose more reliability and accuracy of the results than the DFT method. Thus, the original MP2 method is more reliable than the DFT method, though, in general, it provides accuracy comparable to that of the best DFT protocols. The CCSD and CCSD(T) methods allow us to calculate the *δ*(^31^P) with errors of only 1–5 ppm against experiments, but, unfortunately, their application is restricted by the compounds of very moderate sizes for now. However, we believe that with the progress of both computer technology and parallel coding, the main limitations of coupled-cluster (CC) schemes of higher hierarchy will be effectively alleviated in the near future, and the CC method will become preferable over the DFT method. Indeed, the CC method provides universal systematical convergence to the exact result by increasing the rank of the CC scheme, so that the scopes and limitations of each CC scheme are clearly defined, including the accuracy of the results that one can expect from them. On the other side, the DFT method is a low-cost method and this may seem potentially more demanded in the NMR chemical shift calculations in the future, yet, in our opinion, due to its proclivity to provide the results that are more or less dependent on the XC functional used, the DFT method will, subsequently, be inferior to the CC method and will only be used for some preliminary rapid estimations of the result.

Nowadays, there is an urgent request for advanced computational techniques, specialized tools, and efficient methodologies to account for different factors of accuracy such as solvent, relativistic and vibrational effects, which can be applied in the calculations of *σ*(^31^P)/*δ*(^31^P). The rest of the review will be devoted to these issues.

## 4. Specialized Basic Sets for Calculating ^31^P NMR Chemical Shifts

The choice of the quantum chemical method is believed to be if not the main, then one of the most important factors of accuracy, determining up to 50% of the result [153]. At the same time, the second factor of accuracy, which is of no less importance, is the basis set being used for the representation of the atomic orbitals of atoms in the quantum chemical calculation of NMR shielding constants. Naturally, different basis sets have different performances for different methods [160]; however, no matter what quantum chemistry method is employed, using basis sets with little flexibility in the important regions for the NMR calculation provides poor results. Of course, a large basis set, which is close to being complete, will realize the full potential of the chosen method, but will also require a steep computational cost. A small basis set, on the other hand, is computationally efficient but will introduce considerable errors if it fails to properly describe certain orbitals in specific spatial regions (in terms of the distance to the nuclei) actively participating in the NMR shielding calculation [135].

In particular, the paramagnetic contribution to shielding constant involves the paramagnetic spin–orbit (PSO) operator. The MO matrix elements of this operator were found to be strongly dependent on the number of tight *p*-functions present in the basis sets. This was shown on the calculations of the PSO term of nuclear spin–spin coupling constants [161]. Therefore, regardless of the quantum chemistry method used, the basis set convergence of NMR shielding may considerably be improved by adding more of this kind of functions [135].

Helgaker et al. [162] presented a systematical study on the convergence of nuclear shielding constants in nine simple molecules, including PH_3_, calculated at the GIAO-CPHF level with five different families of energy-optimized basis sets, against the GIAO-CPHF CBS limit. The authors came to an important conclusion, which is as follows: for an accurate calculation of nuclear shielding constants, a basis set of at least valence triple-zeta quality and with at least one set of polarization functions is needed.

**Figure 7:**
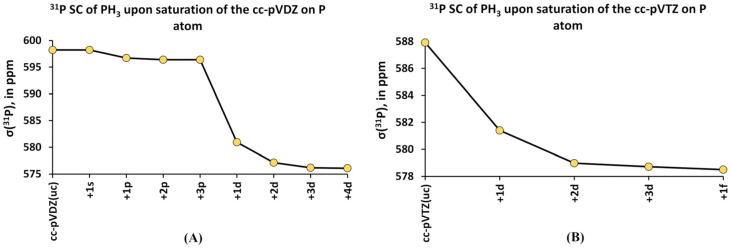
Changes in *σ*(^31^P) of PH_3_ (in ppm) during the expansion of the cc-pVDZ(uc) (**A**) and cc-pVTZ(uc) (**B**) basis sets used on phosphorus atom. Reproduced from Rusakov et al. [163] with permission from the Royal Society of Chemistry.

Rusakov et al. also investigated the behavior of the *σ*(^31^P) calculated within the GIAO-DFT(PBE0) method upon the expansion of various functional spaces of the uncontracted cc-pVDZ(uc) and cc-pVTZ(uc) basis sets used on phosphorus atoms, using the example of the PH_3_ molecule [163]. The original compositions for the cc-pVDZ(uc) and cc-pVTZ(uc) basis sets for phosphorus atom were as follows: (12s8p1d) and (15s9p2d1f), respectively. Hydrogen atoms were also presented with the cc-pVDZ(uc) and cc-pVTZ(uc) basis sets and were kept fixed during the saturation of the basis sets used on phosphorus. The saturation of Dunning’s basis sets on the phosphorus atom was carried out in the tight region of each angular space by means of applying the geometrical progression or even-tempered recurrent ratio [164]. The results of saturation are presented in Figure 7, from which one can see that the addition of one *p*-function to the cc-pVDZ(uc) basis set caused only a small change within a couple of ppm.

This observation correlates with the finding of Antušek et al. [148], who also noticed that further expansion of the cc-pVXZ(s,p-unc) basis set in the *s*- and *p*-shells results in no changes. At the same time, the saturation of the *d*-shell in both cases caused a dramatic change in *σ*(^31^P). Accordingly, adding two *d*-functions to both the cc-pVDZ(uc) and cc-pVTZ(uc) basis sets led to a decrease in the σ(^31^P) of the PH_3_ molecule by about 19 and 9 ppm in total. This means that having at least three (one original plus two additional) and four (two original plus two additional) *d*-functions at the double- and triple-zeta Dunning’s basis sets, respectively, is mandatory to obtain a converged result. This result is coherent with the observation of Helgaker et al. [162] about the necessity of using at least one set of polarization functions and that of Chesnut and Foley [165], who also have come to the conclusion that the addition of the first and second set of *d*-functions has very large effect on *σ*(^31^P).

A beneficial effect, coming from uncontracting the *p*-functional space and the expansion of the first polarization shell with one or even two sets of tight *d*-functions, speaks in favor of potentiality for devising specific basis sets optimized for the effective calculations of *σ*(^31^P). Indeed, standard energy-optimized Gaussian basis sets that have been de facto used in the majority of NMR shielding constant calculations for the last several decades do not represent an effective approach. Such basis sets have been designed by minimizing the basis set parameters (exponents and contraction coefficients) with respect to atomic and/or molecular energies. This makes them a viable and extremely effective tool for the approximation of molecular orbitals in energetically important regions, which, without a doubt, provides the most effective approach to all calculations directly connected with energy, including, for example, the dissociation, relative, binding, and reaction energies, ionization potentials, and electron affinities, but for the shielding constant calculations these basis sets are not effective. As was mentioned above, the calculation of shielding constants poses its own specific requirements on the basis set; thus, either very large standard basis sets are to be employed to fully cover all the incompleteness in the needed regions, or one has to apply artificial manipulations with selected basis set functional subspaces. In both cases, this cannot be thought of as a rational approach, especially today, when large-scale computations of important biologically active compounds represent the highest interest.

Jensen was the first who commenced systematical development of the specialized basis sets for NMR shielding constant calculations. He presented his now famous shielding constant-specialized (*σ*-oriented) basis sets (aug)-pcS-*n* (*n* = 0–4) for elements of 1–3 periods (H-Ar) in 2008 [135]. The (aug)-pcS-*n* basis sets were created on the ground of his previously obtained energy optimized (aug)-pc-*n* basis sets [166,167,168]. The number *n* in the notation of his basis sets, (aug)-pc(S)-*n*, indicates the highest level of polarization functions included in the basis set beyond the atomic system.

Jensen started the design of *σ*-oriented basis sets by performing a systematic analysis of the convergence of the nuclear shielding constants calculated with uncontracted pc-*n* (*n* = 0–4) basis sets at the GIAO-DFT(B3LYP) level, with gradual expansion of the basis sets with additional diffuse and tight functions. He showed that additional tight *p*-functions are the only ones to have a significant effect on the values of paramagnetic contributions to the nuclear magnetic shielding constants. Diffuse functions also provided a significant effect in some cases, which was attributed to possible requirements of the Zeeman orbital operator or simply to the fact that polar systems with lone electron pairs might require diffuse functions for a correct description. The diamagnetic contributions to the phosphorus shielding constant did not reveal any additional requirements.

Jensen has found that one additional tight *p*-function to the (aug)-pc-*n* basis sets should be enough to reach an acceptable level of completeness of the *p*-shell. In this way, in order to create the (aug-)pcS-*n* basis sets, he added one tight *p*-type function to the uncontracted (aug-)pc-*n* basis sets for all considered elements, including phosphorus. The optimum exponent of the added tight *p*-function for the (aug-)pcS-*n* basis sets was determined based on the DFT calculations of *σ* of a given nucleus in a selection of molecules, employing the optimization procedure that was aimed at maximizing the change in the nuclear shielding constant relative to the corresponding value calculated with the regular (aug-)pc-*n* basis set. In order to obtain the (aug-)pcS-*n* basis sets in contracted form, Jensen employed the general contraction scheme [169], which implies that all primitive functions are allowed to contribute to all contracted functions. Seven years later, Jensen presented the segmented contracted (when each primitive function is allowed to contribute to only one contracted function [170,171]) basis sets optimized for nuclear magnetic shielding constant calculations, the pcSseg-*n* (*n* = 0–4), for the atoms of 1–4 periods [158]. Both series, the (aug-)pcS-*n* and (aug-)pcSseg-*n*, are efficient in the calculations of *σ*(^31^P)/*δ*(^31^P) within the DFT method. It is also worth noting that the (aug-)pcSseg-*n* series is now accepted to be more advanced in the sense of efficiency than the (aug-)pcS-*n* series.

**Figure 8:**
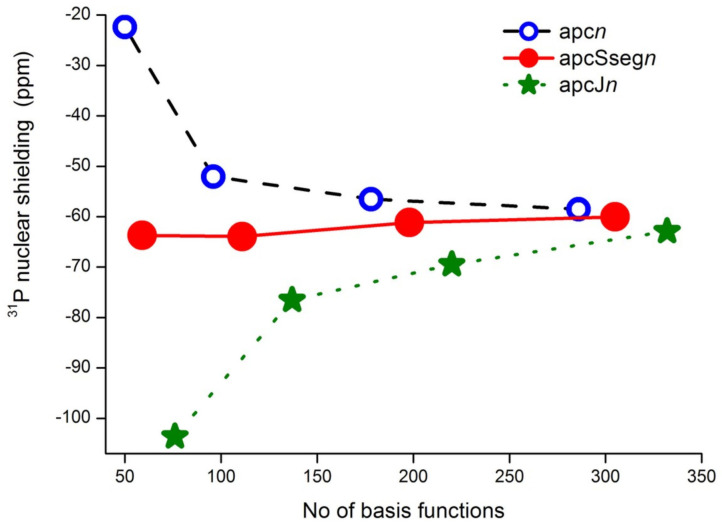
The performance of Jensen’s aug-pc-*n* (apc*n*), aug-pcSseg-*n* (apcSseg*n*), and aug-pcJ-*n* (apcJ*n*) basis sets, with *n* = 0–4, applied to the calculation of *σ*(^31^P) in PN molecule within the GIAO-DFT(B3LYP) method. Reproduced from Kupka et al. [156] with the permission of MDPI.

The application of Jesen’s *σ*-oriented basis sets to the calculation of *σ*(^31^P)/*δ*(^31^P) can be found in several literature sources; however, such examples are only few and can be found in the material published no earlier than the last decade. One such interesting article was presented by Kupka et al. [156], who, in particular, performed the investigation of the convergence behavior of the *σ*(^31^P) of the PN molecule calculated at the GIAO-DFT(B3LYP) level with the aug-pc-*n*, aug-pcSseg-*n*, and aug-pcJ-*n* (*n* = 0–4) basis sets in order to find out whether there would be an analogously irregular convergence, as has been observed in the case of Dunning’s aug-cc-pVXZ, X = D, T, Q, 5, 6 series (see in Figure 6). The convergences of all three series of basis sets are shown in Figure 8.

As can be seen from Figure 8, the use of nonspecialized energy-optimized aug-pc-*n* basis sets and aug-pcJ-*n* basis sets, which are specialized for another NMR property (namely, spin–spin coupling constants), resulted in smooth but slow convergence, while a specialized series, aug-pcSseg-*n*, provided very fast convergence. The same can be said about the convergence of the *σ*(^31^P) of PH_3_ that is shown in Figure 6, for which case the aug-pcSseg-2 basis set already gives the converged value.

It is important to take into account that polarization-consistent *σ*-oriented (aug-)pcS-*n* and (aug-)pcSseg-*n* basis sets were optimized within the DFT method, and their performance can be different when wave function-based methods such as CC are applied. In this respect, Kupka et al. [156] obtained interesting theoretical data for the CBS limits of the *σ*(^31^P) of PH_3_ and PN molecules calculated with the aug-pcSseg-*n* basis set series (alongside with CBS limits estimated for the aug-cc-pVXZ and aug-cc-pCVXZ series) within the DFT(B3LYP) and CCSD(T) methods. For all basis set series, including aug-pcSseg-*n*, the CBS limits calculated within these two conceptually different methods turned out to be essentially different. For the precise data, see Table 1.

**Figure 9:**
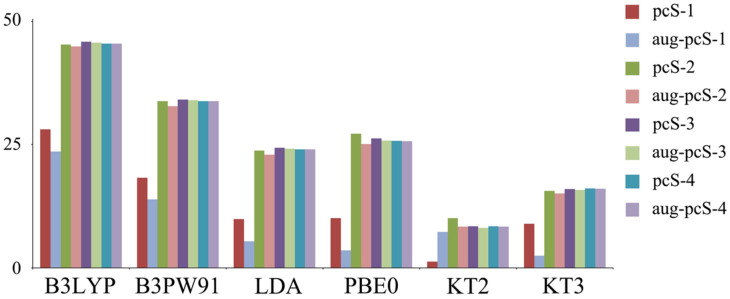
Absolute errors of the phosphorous absolute shielding constant in phosphine calculated at the GIAO-DFT level using different functionals in combination with Jensen (aug-)pcS-*n* (*n* = 1–4) basis sets, compared with the CCSD(T) result. Reproduced from Fedorov et al. [132] with the permission of John Wiley & Sons, Inc.

Fedorov et al. [132] also compared the performance of the Jensen *σ*-oriented basis sets within the DFT and CCSD(T) approaches. The *σ*(^31^P) of the PH_3_ molecule was calculated within the GIAO-DFT method with six XC functionals using the (aug-)pcS-*n* (*n* = 1–4) basis set series. The obtained DFT values were compared to the most accurate value for that time of 606.11 ppm, which was calculated by Lantto et al. [133] at the GIAO-CCSD(T)/cc-pwCV5Z level without relativistic, rovibrational, or temperature corrections. Figure 9 shows the absolute errors of *σ*(^31^P) in phosphine calculated by Fedorov et al. within the DFT method with the (aug-)pcS-*n* basis sets against the “ideal” CCSD(T) value.

**Figure 10:**
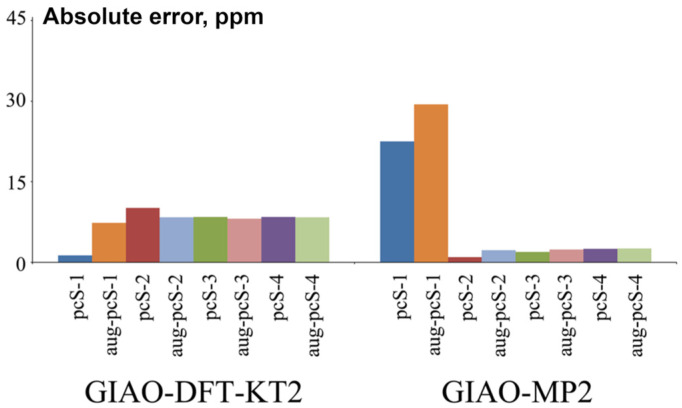
Absolute errors of the phosphorous absolute shielding constant in phosphine calculated at the GIAO-DFT-KT2 and GIAO-MP2 levels using (aug-)pcS-*n* (*n* = 1–4) basis sets compared with the CCSD(T) result. Reproduced from Fedorov et al. [132] with the permission of John Wiley & Sons, Inc.

It can be seen from Figure 9 that, in general, the absolute deviations for the majority of XC functionals are rather high when considering the (aug-)pcS-*n* basis sets with the number (*n*) more than one. In the case of *n* = 1, there must be a serendipitous cancelation of errors that would hardly emerge again when dealing with the other phosphorus compounds. Based on the more realistic results obtained with the (aug-)pcS-*n* with *n* = 2–4, it can be said that the GGA functionals of Keal and Tozer, KT2 and KT3, show essentially better results as compared with the more common functionals PBE0, LDA, B3LYP, and B3PW91. The least absolute deviation of the KT2 values obtained with (aug-)pcS-*n* (*n* = 3–4) can be estimated as approx. 7–8 ppm with respect to the ideal CCSD(T) value. At the same time, in going from the DFT to MP2 method, the difference with the CCSD(T) method dramatically decreases (and corrects the situation with the (aug-)pcS-1 basis sets). The performance of the (aug-)pcS-*n* (*n* = 1–4) basis set series for the *σ*(^31^P) of PH_3_ within the MP2 method estimated in terms of the absolute deviation against the “ideal” CCSD(T) value is shown in Figure 10.

From all these data, it can be concluded that, in general, the performance of the (aug-)pcS-*n* and (aug-)pcSseg-*n* (*n* = 0–4) series of basis sets is essentially different in the calculations of *σ*(^31^P) within the methods of the DFT and the wave function-based correlated theories, such as CC or MP.

Rusakov et al. [163] has also proposed the basis sets optimized for phosphorus NMR chemical shift calculations, called pecS-*n* (*n* = 1, 2). The compositions of the uncontracted pecS-*n* (*n* = 1, 2) basis sets were formed on the basis of cc-pVDZ(uc) and cc-pVTZ(uc) by expanding the *p*-shell with one *p*-function and *d*-shell with two *d*-functions. The exponents and contraction coefficients for the pecS-*n* basis sets were generated with the property-energy consistent (PEC) method that was introduced in Rusakov’s earlier paper [172] and has proven useful in the creation of efficient NMR property-oriented basis sets [173,174,175,176,177]. New basis sets were optimized using the GIAO-DFT method with the B97-2 functional.

The PEC method of optimization of property-oriented basis sets is a recent finding that was first published in 2021. This method implies the optimization of basis sets in relation to a certain molecular property, provided that the least possible total molecular energy is achieved [172]. Exponents are randomly generated around the starting basis set via Monte Carlo simulations. In this respect, Monte Carlo simulation is not bound to find only one extremum in the close vicinity to the starting point (like in the case of the Newton–Raphson algorithm [178]), but represents a neural-type random search of the isoline of some ‘‘ideal’’ property value that is formed by the intersection of the ‘‘ideal’’ property plane with the multi-argument property surface defined in the space of varying exponents. Therefore, PEC is most suitable for highly nonlinear optimization problems like finding optimized exponents for a given property.

**Figure 11:**
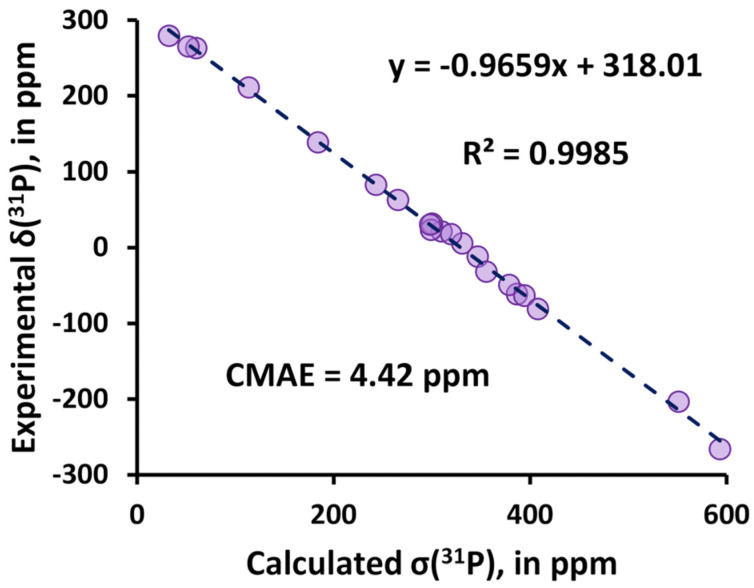
Correlation plot of *σ*(^31^P) calculated at the GIAO-DFT(B97-2)/pecS-2 level in twenty testing compounds against the corresponding experimental *δ*(^31^P). Reproduced from Rusakov et al. [163] with the permission of the Royal Society of Chemistry (London, UK).

Extensive benchmark calculations that were carried out within the GIAO-DFT method, taking into account the solvent and relativistic effects, showed that the pecS-1 and pecS-2 basis sets for phosphorus atom provide very good accuracy of calculated *δ*(^31^P) values against the experiment, characterized by MAEs of about 7.03 and 4.42 ppm, respectively. In particular, the correlation plot between the calculated *σ*(^31^P) and experimental *δ*(^31^P) of twenty versatile phosphorus molecules considered in Rusakov’s work [163] is shown in Figure 11.

**Figure 12:**
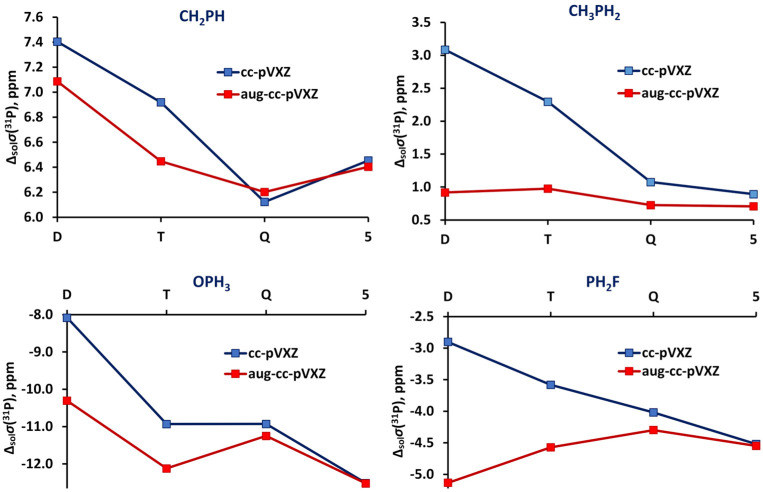
The dependence of solvent corrections to *σ*(^31^P) in CH_2_PH, CH_3_PH_2_, OPH_3_, and PH_2_F molecules on the valence splitting of Dunning’s basis sets, cc-pVXZ and aug-cc-pVXZ (X = D, T, Q, and 5). Solvent corrections were calculated for water as the solvent. Reproduced from Rusakov et al. [177] with the permission of the Royal Society of Chemistry.

The augmented aug-pecS-*n* (*n* = 1 and 2) basis sets for *σ*(^31^P)/*δ*(^31^P) calculations were presented recently [177]. In that paper, it was shown that the diffuse functions exert highly positive effects not only on the *σ*(^31^P) per se but also on the solvent corrections to *σ*(^31^P). This is demonstrated in Figure 12, where one can see striking differences in the convergence behavior of the solvent corrections to *σ*(^31^P) of four molecules, CH_2_PH, CH_3_PH_2_, OPH_3_, and PH_2_F, calculated at the GIAO-DFT(B97-2) level of theory with two Dunning’s basis set series, the cc-pVXZ and aug-cc-pVXZ (X = D, T, Q, and 5).

It is worth mentioning that in the cc-pVXZ and aug-cc-pVXZ basis set series, parameter X represents the cardinal number which quantifies the number of functions representing valence atomic orbitals, with higher numbers generally meaning more functions, better accuracy, and higher computational cost. For example, the double- (X = D), triple- (X = T), quadruple- (X = Q), and pentuple (X = 5)-zeta level implies two, three, four, and five basis functions for each atomic orbital (AO) in the valence shell, respectively. Accordingly, for example, the total number of basis set functions in spherical coordinates for the cc-pVXZ/aug-cc-pVXZ basis sets for phosphorus atom with X varying from D to five is as follows: 18/27, 34/50, 59/84, and 95/131.

Thus, Figure 12 shows that the aug-cc-pVXZ series provides significantly better convergence than the cc-pVXZ series with cardinal number X. This picture was found to be the same for the other solvents of varying polarity. Considering this fact as a useful pointer, the authors of paper [177] have proposed the augmentation of the phosphorus pecS-*n* (*n* = 1 and 2) basis sets on the basis of the isotropic dipole polarizability used as the main target property in the PEC optimization of the added diffuse exponents.

**Figure 13:**
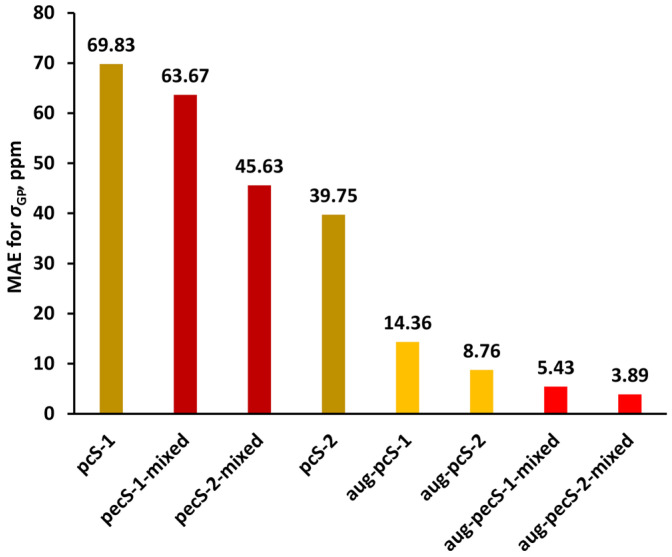
MAEs for *σ*(^31^P) calculated in the gas phase (σ_GP_), using the GIAO-DFT(B97-2) level of theory with different basis sets in five phosphorus-containing anions, evaluated in relation to the reference data obtained at the GIAO-DFT(B97-2)/aug-pcS-4 level. Different colors of the bars correspond to different families of basis sets (red—the (aug-)pecS-*n* family, yellow—the (aug-)pcS-*n* family); lighter shades of these colors designate the augmented versions of the basis sets. Reproduced from Rusakov et al. [177] with the permission of the Royal Society of Chemistry.

Apart from being useful for improving the accuracy of the calculation of the solvent corrections to *σ*(^31^P) of neutral molecules, the aug-pecS-*n* basis sets have been proven to significantly increase the precision of the calculation of the *σ*(^31^P) of anions, as compared with the nonaugmented analogies. The latter can be seen in Figure 13, which shows the MAEs for *σ*(^31^P) calculated in the gas phase at the GIAO-DFT(B97-2) level of theory with different basis sets (including pecS-*n* and aug-pecS-*n*) in five phosphorus-containing anions (cyaphide anion [P≡C]^−^, hydrogen phosphite anion [HPO_3_]_2_^−^, orthophosphate anion [PO_4_]_3_^−^, methylenephosphine anion [CH_2_P]^−^, and phosphine anion [PH_2_]^−^), evaluated in relation to the reference data obtained at the GIAO-DFT(B97-2)/aug-pcS-4 level.

Another interesting concept of generating basis sets for magnetic properties, including shielding constants, was proposed by Manninen and Vaara, who used the completeness-optimized basis functions to cover the important exponential range for the given molecular property [179]. In contrast to the conventional method of basis set generation, i.e., optimizing a fixed number of exponents in order to produce the minimal possible atomic (or molecular) energy, in the completeness-optimization scheme, the exponents for each angular momentum shell were obtained by minimizing the deviation from the unity of the completeness profile [180]. In a certain exponent interval [*α*_min_, *α*_max_], this quantity represents a measure of a given basis set’s ability to describe all details of the wave function in the corresponding distance range from the atomic nuclei.

Manninen and Vaara developed an optimization procedure for basis sets, in which the deviation from unit completeness is associated with the corresponding error in a given molecular property. The ultimate goal of this approach was to produce a basis set that would provide minimal deviation from the unit completeness with the least possible number of functions. It is interesting to note that the completeness-optimized exponent sets are element-independent, in the sense that information on the atomic structure is not used in selecting the exponents. Thus, equivalent completeness-optimized basis sets can, in principle, be used for any element, including phosphorus. This concept was applied in the calculation of several magnetic molecular properties by Lehtola and co-authors in a limited series of works [181,182,183]. In particular, Lantto et al. [133] reported on the application of completeness-type optimization of basis set exponents to generating the *σ*-oriented phosphorus basis set and called the new basis set as co-b. This basis set appeared to be very large in size, being of the following structure: (26s22p18d8f). In general, the results presented in Ref. [133] suggest that the co-b basis set provides the nonrelativistic value of *σ*(^31^P) in PH_3_ that is equivalent to that obtained with the cc-pwCV5Z basis set, yet the relativistic values obtained with these two basis sets differ from each other by about 1.5 ppm. It should be kept in mind that the co-b basis set, as well as its reduced version, the co-r, are huge basis sets comparable in size with the uncontracted aug-cc-pwCV5Z and cc-pwCV5Z, respectively; thus, they can be considered as the basis sets in close proximity to the CBS limit, which can be useful in exclusively precise calculations of *σ*(^31^P)/*δ*(^31^P) or when such basis sets are used only on phosphorus atoms under interest with all other atoms of the molecule described with much smaller basis sets. In any case, the full potential of the completeness-based basis sets in the *σ*(^31^P)/*δ*(^31^P) calculations is yet to be discovered.

Franzke et al. [159] proposed the *σ*-oriented segmented contracted relativistic basis sets x2c-SVPall-s and x2c-TZVPall-s suitable for the calculations of the NMR shielding constants of almost all nuclei of the Periodic Table (H-Rn and La-Lu), including phosphorus. These basis sets were developed on the ground of relativistic Karlsruhe basis set x2c-XVPall (X = S, TZ) [184]. In general, the authors adopted the usual way of generating the optimized exponents, though, not without considering special means stipulated by the peculiarities of the two-component level of relativistic theory. To be more precise, the authors started by determining the errors of *σ* calculated using the existing all-electron relativistic Karlsruhe basis sets with respect to the reference even-tempered basis set for around 250 molecules. The latter was generated in an even-tempered manner with the factor of $\sqrt[{4}]{10}$ between its exponents up to very tight functions to obtain very large basis set that would represent the reference set close to the CBS limit. The relativistic x2c-SVPall-2c and x2c-TZVPall-2c basis sets, which take into account spatial splitting of atomic subshells induced by spin–orbit coupling, were selected as the best candidates to be modified to obtain the *σ*-tailored basis sets. Just like in accordance with the well-established fact that tight *p*-functions play an important role in paramagnetic contribution (vide supra), the authors added to both levels of their basis sets per one tight *p*-function for all nuclei. The exponents were optimized in several cycles based on the variational principle via the Newton–Raphson algorithm, so as to reduce the mean absolute error of *σ* in test sets of molecules against the values obtained with the reference basis set. The segmented contraction coefficients were optimized at the X2C level of theory. The x2c-SVPall-s and x2c-TZVPall-s basis sets were further compared to Jensen’s segmented contracted basis set, pcSseg-*n* (*n* = 0–4), based on the percent-wise error measured against the large reference even-tempered basis set. Final calculations of shielding constants of the 3p-elements, including phosphorus, revealed that the x2c-SVPall-s basis set provides theoretical accuracy which is comparable with that of the pcSseg-0 basis set, while the x2c-TZVPall-s basis set gives somewhat better results, providing an accuracy slightly worse than that reached with the pcSseg-1 basis set.

It is with a rare exception that one can find the calculations of *σ*(^31^P) carried out using the x2c-XVPall-s family of basis sets. One such example has been reported by Kupka et al. [156], who has applied the x2c-SVPall-s, x2c-TZVPPall-s, and x2c-QZVPPall-s basis sets to the calculations of *σ*(^31^P) of PH_3_ carried out within the HF-SCF, DFT(B3LYP), and CCSD(T) approaches. The demonstration of their performance for the PH_3_ molecule at the CCSD(T) level of theory is shown in Figure 6 (vide supra), from which it can be seen that, although the x2c-SVPall-s basis set for phosphorus is slightly larger than the aug-pcSseg-0, its performance is considerably better than that of aug-pcSseg-0. At the same time, the other two basis sets of this type, the x2c-TZVPPall-s and x2c-QZVPPall-s, which represent the basis sets of the triple- and quadruple-zeta quality containing additional polarization functions, showed a comparable accuracy with that provided by much larger basis sets of quadruple-zeta quality, such as aug-pcSseg-3 and aug-pCVQZ. So, it is not that simple to make an unbiased conclusion about the efficiency of the x2c-XVPall basis set series for the *σ*(^31^P) calculations. Based on the first calculations of Franzke et al. [159], one could have concluded that the x2c-XVPall-s (X = S, TZ) basis sets provide inferior accuracy to that of the pcSseg-*n* basis sets with comparable sizes, yet Kupka et al. [156] demonstrated otherwise. Thus, what is really absent in this account is a solid statistical estimation of the performance of the x2c-XVPall-s basis set family based on large benchmark data obtained for the *σ*(^31^P)/*δ*(^31^P) of diverse representative systems calculated with different methods.

Accurate ab initio correlated wave function-based methods such as the CCSD or CCSD(T) models of CC theory become prohibitive for the calculations with more than 500–600 basis set functions, while the DFT calculations are much less demanding, allowing the handling of several thousand basis set functions on modern computers. However, even this quantity cannot be considered enough, if we are speaking of phosphorus NMR shielding constant calculations of large biologically active phosphorus-containing compounds.

In this regard, one of the alternatives to reduce the size of the basis set space is to resort to the so-called locally dense basis set (LDBS) approximation, which consists in applying a large, high-quality basis set on particular atoms of interest and on much smaller basis sets elsewhere in the molecule [185,186,187,188,189].

As strange as it may seem, the LDBS approach was probed in the *σ*(^31^P)/*δ*(^31^P) calculations much later than the first DFT calculations had been reported in the mid-1990s, provided that the idea of using the LDBS in the shielding constant calculations had been put forward in 1989 [185]. Indeed, the LDBS approximation is utterly justified for the NMR shielding constants. Notwithstanding the fact that these are dependent on all electrons in the molecule, the PSO operator entering the paramagnetic term is local in nature. This makes NMR shielding constants a local property (like an electric field gradient EFG, for example), which strongly depends on the quality of the basis set used on the NMR atom under interest and its close surrounding area, bearing, at the same time, only a loose or even vanishingly weak dependence on the basis sets used on the atoms located farther beyond the vicinity boundary. The interest in the application of the LDBS schemes to phosphorus shielding constant calculations was inflamed by Fedorov et al. [132] in 2014. Naturally, the authors chose phosphorus to represent the “atom of interest,” while the rest of atoms in molecules were considered to be the atoms of less importance.

**Figure 14:**
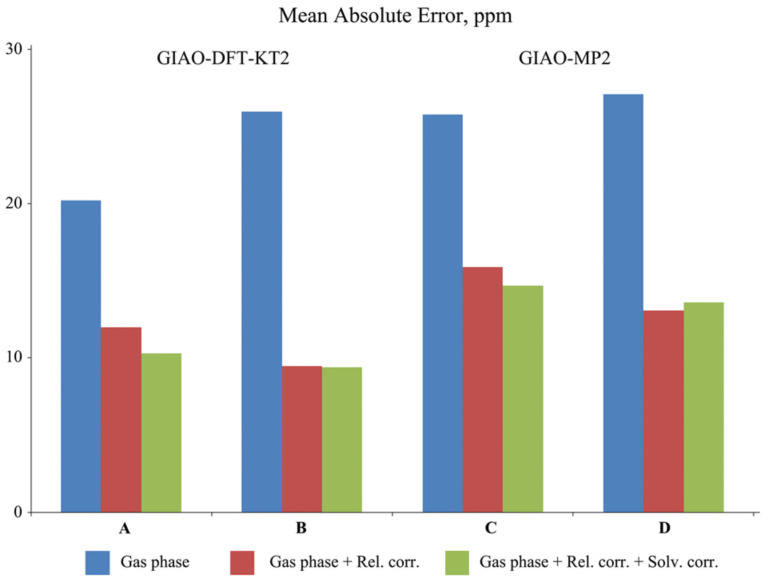
The MAEs of *σ*(^31^P) of 53 phosphorus compounds calculated within different LDBS computational schemes: (A) GIAO-DFT(KT2)/6-311++G(3d,2p)//6-311++G(d,p), (B) GIAO-DFT(KT2)/pcS-3//pcS-2, (C) GIAO-MP2/6-311++G(3d,2p)//6-311++G(d,p), and (D) GIAOMP2/pcS-3//pcS-2. Reproduced from Fedorov et al. [132] with the permission of John Wiley & Sons, Inc.

The *σ*(^31^P) values were calculated in the representative benchmark set of 13 diverse phosphorous-containing compounds, encompassing trimethylphosphine, trimethylphosphine oxide, trimethylphosphine sulfide and their chlorine analogs; sterically strained phosphirane and phosphetane; and unsaturated and aromatic phosphorous-containing heterocycles such as phosphole, isophosphole, oxazaphosphole, and triazatriphosphinine. In that way, a variety of typical bonding environments were well reproduced to test the proposed LDBS schemes on the calculations of *σ*(^31^P) that were carried out at the GIAO-DFT(KT2) and GIAO-MP2 levels. Two LDBS schemes were considered, namely, the 6-311G++(3d,2p)/6-311++G(d,p) and pcS-3/pcS-2, where the basis set before and after slash was used on phosphorus and on the rest of atoms, respectively. These LDBS schemes provided results varying in accuracy, which were also found to depend on the method used for the *σ*(^31^P) calculation and the relativistic and solvent corrections either taken into account or not. The MAEs against the experiment calculated for *σ*(^31^P) in 53 phosphorus compounds with the two mentioned LDBS schemes are shown in Figure 14.

As one can see from Figure 14, the LDBS scheme pcS-3//pcS-2 demonstrates the best accuracy, no matter which of the two methods of electronic structure calculation is used. The lowest MAE provided by the pcS-3//pcS-2 scheme is about 10 and 12 ppm for the DFT(KT2) and DFT(MP2) methods, given that solvent corrections are taken into account. The 6-311++G(3d,2p)//6-311++G(d,p) scheme was shown to be only slightly inferior in accuracy. This is an interesting result, considering the fact that the pcS-3//pcS-2 scheme contains a considerably larger number of basis set functions than the 6-311++G(3d,2p)//6-311++G(d,p) scheme does. The accuracy achieved by Fedorov et al. when applying the proposed LDBS schemes within the DFT and MP2 methods is comparable to that reached by others in earlier calculations performed with the same methods and full basis sets [118,119,120]. So, the findings of Fedorov et al. indicate that employing the LDBS approach does not take the results out of the ordinary accuracy boundaries, thus proving the expediency of the LDBS approach.

**Figure 15:**
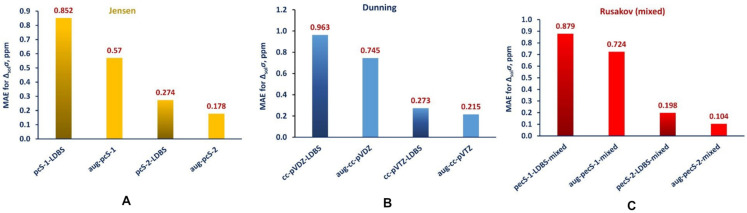
MAEs evaluated for the solvent corrections to the ^31^P NMR shielding constants of 12 molecules obtained within the LDBS schemes based on Jensen’s (**A**), Dunning’s (**B**), and Rusakov’s’ (**C**) basis sets, including their full augmented analogs, against the reference data. Reproduced from Rusakov et al. [177] with the permission of the Royal Society of Chemistry.

In the paper of Rusakov et al. [177], the LDBS issue was also briefly considered in the light of the acceleration of *σ*(^31^P)/*δ*(^31^P) calculations. The paper was intended to demonstrate the dependence of the solvent corrections to *σ*(^31^P) on the basis set used on the example of various phosphorus-containing molecules. In course of that work, a new augmented *σ*-oriented basis set for the phosphorus atom, the aug-pecS-*n* (*n* = 1 and 2), was proposed. Various LDBS schemes, including those based on Dunning’s cc-pVXZ (X = D, T), Jensen’s pcS-*n* (*n* = 1, 2), and Rusakov’s pecS-*n* (*n* = 1, 2) basis sets, were considered. In these, the phosphorus atom was described with the augmented versions of basis sets, while the rest of the atoms were described with their nonaugmented versions. The results revealed that the LDBS approach fully justifies itself in the phosphorus shielding constant calculations and the solvent corrections all the same, especially when one uses the basis sets of triple-zeta quality. This observation is demonstrated below, in Figure 15, which shows the MAEs for the theoretical values of solvent corrections to *σ*(^31^P) of 12 phosphorus compounds calculated within the GIAO-DFT(B97-2) method using the basis sets of three different types, as well as the corresponding LDBS schemes built of them. The MAEs were evaluated against the reference data obtained with the aug-pcS-4 basis set that is close to the CBS limit.

Overall, the LDBS approach represents a viable tool for efficient calculations of *σ*(^31^P)/*δ*(^31^P), reserving great potential for the future calculations of large-sized phosphorus compounds of industrial interest.

Concluding this section, we would like to underscore that using specialized basis sets that contain the least number of needed basis set functions with optimal exponents for a given property, for example, NMR shielding, will prove useful in the calculations within the DFT method and, more importantly, within the hierarchy of the CC models. Severe computational limitations imposed by the latter method can surely be alleviated to some extent by using compact *σ*-oriented basis sets, which provide an accuracy of results comparable to that of rather larger, nonspecialized energy-optimized basis sets.

## 5. Geometry Factor Effect on ^31^P NMR Shielding Constants/Chemical Shifts

Modern high-quality quantum chemical calculations of phosphorus NMR chemical shifts are capable of providing theoretical values deviating from the experimental data by no more than 3–5 ppm [163], which is supposed to represent an excellent accuracy, given that typical width of the ^31^P NMR chemical shift scale is about 650–700 ppm. At the same time, it is well-known that by varying the methodology (method/basis set) applied in the geometry optimization stage, one can obtain changes in the resultant theoretical estimations of ^31^P NMR chemical shifts of up to 10 ppm [136,141,190,191]. Such a profound change is comparable in magnitude to the average errors of modern quantum chemical calculations of *δ*(^31^P). Therefore, an improvement of the quality of equilibrium geometry on which the phosphorus chemical shifts are calculated is capable of considerably changing the theoretical results.

Specifically, the geometry factor of accuracy is of utmost importance when one deals with difficult cases, wherein even half of a ppm plays a decisive role in resolving a structure by the ^31^P NMR spectra analysis. In light of this matter, it is so very strange to witness how practically no attention is paid to the geometric factor of accuracy in modern calculations of *δ*(^31^P), though, the problem per se was recognized long time ago. 

The first attempts to make a link between the geometrical parameters and *δ*(^31^P) in phosphorus compounds were made in the 1970–1980s [165,192,193,194,195,196]. These pioneering works indicated a strong correlation between NMR shielding constants and geometrical parameters, such as bond lengths, bond angles, and dihedral angles. Later, Chesnut and Quin [197] corroborated a strong influence of the geometry factor effect on the calculated phosphorus chemical shifts on the example of PCl_3_ and PCl_5_ molecules, for which it was found that the level of electronic structure theory used at the geometry optimization stage is utterly important. In this respect, Chesnut and Foley [165] argued that any discussion of computational techniques for *δ*(^31^P) must be carried out in regard to the geometry of the molecule being studied.

Evidently, the accuracy of the equilibrium geometry is determined by two general computational factors, the method of electronic structure theory and the basis set used. These two should be considered together, on an equal footing, as the deficiency of the former cannot be fully alleviated by the advantages of the latter and vice versa, though, sometimes we witness a serendipitous cancelation of method/basis set errors. In particular, Helgaker et al. [198] has found that, in general, bond lengths are contracted by the improvements of the basis sets used in the geometry optimization procedure and stretched by the improvements of the correlation treatment within the geometry optimization method. The same observation about the part concerning the quality of the basis sets used at the geometry optimization stage, has been reported by Rusakov et al. [199] in regard to the optimization of phosphorus-containing compounds.

Given that the majority of contemporary researchers are deeply involved in studying biologically active large-sized phosphorus compounds, both the geometry optimization and the NMR calculations are commonly carried out within a rather accurate and operationally cost-effective DFT method. Therefore, it is the combinations of various DFT exchange–correlation functionals with different basis sets applied at the geometry optimization stage which represent the greatest interest nowadays. Although such works are very scarce in number for now, it is very encouraging that they are starting to emerge in the literature at all.

In particular, Fukal et al. [139] carried out the interpretation the ^31^P NMR spectra of compounds **11**–**14** (see Figure 2) with the aid of NMR quantum chemical calculations. The *δ*(^31^P) were calculated at the GIAO-DFT(B3LYP)/pcS-4 level of theory at different equilibrium geometries, obtained within the DFT(B3LYP) method with the 6-31+G(d), 311++G(3df,3pd), and pcS-4 basis sets. From the presented data, one can conclude that the difference between *δ*(^31^P) obtained with the DFT(B3LYP)/6-31+G(d) and DFT(B3LYP)/6-311++G(3df,3pd) [or DFT(B3LYP)/pcS-4] geometries can reach the magnitude of as much as 3 ppm.

Latypov et al. [136] presented a theoretical study of the *δ*(^31^P) of 34 molecules using different equilibrium geometries. The equilibrium geometries were calculated at the HF, DFT, and MP2 levels of theory using a wide series of Pople’s basis sets: starting from the 6-31G to 6-311++G(3df,3pd). From the presented data, one can conclude that the tendencies of the geometry factor of accuracy toward the basis sets used in the geometry optimization stage are cardinally different for various methods of optimization. However, in general, it was shown that the most reliable geometries can be obtained within the PBE0 and MP2 methods with the 6-311++G(3df,3pd) basis set. Moreover, it can be assumed that the addition of several sets of polarization functions to both hydrogen and non-hydrogen basis sets is of great importance.

In a continuation of their work [136], Latypov and his colleagues recently reported on the geometry factor studies within the DFT-based computational protocols in the application to *δ*(^31^P) calculations of nickel and palladium complexes with small- and medium-sized organophosphorus ligands [190,191]. In Ref. [190], it was mentioned that the correlation plots of the theoretical vs. experimental chemical shifts in phosphorous nuclei do not change when going from the 6-31+G(d)(SDD for transition metals) to def2-TZVP(SDD) basis set applied at the geometry optimization stage of phosphorus compounds. As a main result of the mentioned study, the authors proposed the combination of PBE0/6-311G(2d,2p)(SDD)//PBE0/6-31+G(d)(SDD) as a reliable scheme of the computation of *δ*(^31^P) that is capable of providing an RMSD of ca. 7 ppm. However, the results of more recent work of Latypov et al. [191] revealed insufficient accuracy of the earlier proposed computational scheme applied to the calculations of *δ*(^31^P) in palladium complexes with ligands containing P=O moiety. To resolve the problem, the authors considered the most influential factors of accuracy, including the geometry factor effect. In this respect, it was found that using the triple-zeta quality basis sets and the addition of at least one polarization function at the geometry optimization stage considerably improves the accuracy, reducing the systematic underestimation of the phosphorus chemical shifts by a half.

Gao et al. [142] carried out a systematic benchmarking of *δ*(^31^P) predictions for 35 phosphorus-containing molecules using different DFT methods and applying the linear regression model. It follows from the reported data that there is indeed a difference between the accuracies of theoretical phosphorus chemical shifts obtained at the equilibrium geometries of different qualities. In particular, using the 6-311+G(2d,p) basis set instead of the 6-31+G(d,p) basis set in the geometry optimization stage results in a noticeable positive effect on the final accuracy of theoretical predictions of ^31^P NMR chemical shifts.

Prochnow and Auer [150] investigated the dependence of *σ*(^31^P) on the choice of equilibrium geometries. They considered five small molecules containing phosphorus atoms in typical bonding environments, including single, double, and triple bonds, as well as different heteroatoms such as oxygen and fluorine. Three computational schemes were applied to the calculation of their equilibrium geometries, namely, MP2/cc-pVTZ, CCSD(T)/cc-pVTZ, and CCSD(T)/cc-pVQZ, while all *σ*(^31^P) values were calculated at the GIAO-CCSD(T)/qz3d1f level. The CCSD(T) level of theory has been shown to yield very accurate geometries as compared to the gas-phase electron diffraction (GED) experimental data. The quality of the MP2 structures was found to be substantially lower. At that, the changes in *σ*(^31^P) due to switching between the MP2 or CCSD(T) method used in the geometry optimization stage appeared to be noticeable, of approx. 1–7 ppm for all molecules, except for PN. In the latter case, altering the method of geometry optimization from MP2 to CCSD(T) resulted in increasing its *σ*(^31^P) value by one and a half. The influence of the quality of the basis set used at the geometry optimization stage on the calculated *σ*(^31^P) was also found to be significant. For example, in going from the CCSD(T)/cc-pVTZ to CCSD(T)/cc-pVQZ geometries, the *σ*(^31^P) changed by 1–7 ppm, the upper limit for the PN molecule.

Hersh et al. [141] also raised the issue of the quality of equilibrium geometry in ^31^P NMR chemical shift calculations. The authors considered a training set of twenty-one tri- and tetra-coordinate phosphorus compounds, in particular, cationic compounds, with counterions included. They used the DFT method for both equilibrium geometry and NMR calculations, applying different functionals and basis sets. Phosphorus NMR chemical shifts were calculated by means of the linear regression model. Looking at their results for the *δ*(^31^P) obtained at the same method/basis set but on different equilibrium geometries, it can be concluded that the geometry factor effect coming from using different DFT functionals at the geometry optimization stage does not cause any substantial changes, unless, of course, one half of a ppm is a decisive matter in a given spectroscopical problem. For example, for the *δ*(^31^P) calculated at the B3LYP/6-311+G(2d,p) level, switching from the B3LYP to M06-2X functional during geometry optimization (performed with the 6-31+G(d,p) basis set) lowers the MAE/RMSD errors from 7.0/8.4 to 6.5/8.0 ppm. Apparently, the geometry factor effect is less pronounced in the *δ*(^31^P)-scale than in the *σ*(^31^P)-scale, as there is a partial cancelation of geometry factor errors coming from the reference compound and the sample, or due to the alleviation of systematical errors in the linear scaling models if one uses them to transform shielding constants to chemical shifts.

**Figure 16:**
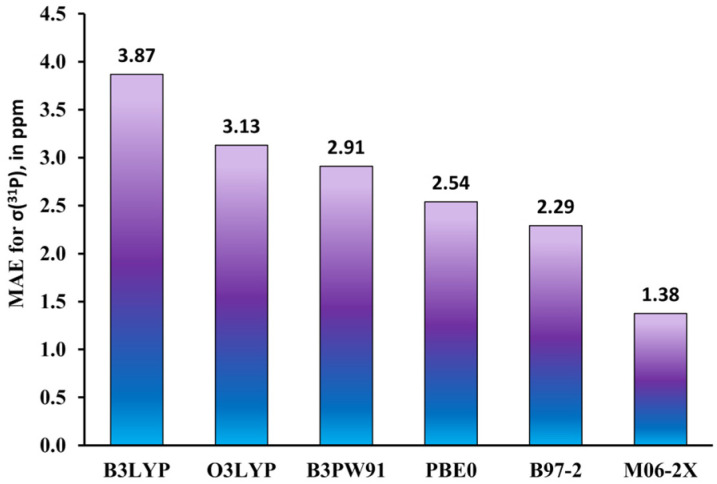
The MAEs (in ppm) of theoretical values of *σ*(^31^P) of various phosphorus-containing molecules calculated at the GIAO-CCSD(T)/pecS-2 level of theory at different equilibrium geometries [obtained with different DFT functionals (listed along the X axis) and the cc-pV5Z basis set] against the values of *σ*(^31^P) calculated at the same level of theory but using the CCSD(T)/cc-pV5Z equilibrium geometries. Reproduced from Rusakov et al. [199] with the permission of AIP Publishing (Melville, NY, USA).

The influence of the quality of equilibrium geometry was recently studied systematically by Rusakov et al. [199]. A statistical analysis of the accuracy of *σ*(^31^P) values calculated on different equilibrium geometries against theoretical reference data (obtained on the geometry of the best achievable level) was performed for a representative series of small phosphorus compounds, including CH_2_PH, CH_3_PH_2_, PH_3_, PH_2_F, and SPH_3_, etc. All *σ*(^31^P) values were calculated at the GIAO-CCSD(T)/pecS-2 level of theory, while the geometries for the NMR calculations were optimized with the GIAO-DFT method using different exchange–correlation functionals with the cc-pV5Z basis set. The best theoretical reference equilibrium geometries were obtained at the CCSD(T)/cc-pV5Z level. Thus, keeping the same theoretical level for the *σ*(^31^P) calculations and varying the DFT models at the geometry optimization stage allowed the authors to select the functionals that provide equilibrium geometries which result in the *σ*(^31^P) value being the closest to the ones obtained on the geometries of the CCSD(T) level. The MAEs evaluated for the values of *σ*(^31^P), calculated upon different DFT geometries against theoretical reference data, are shown in Figure 16.

As can be seen from Figure 16, the most suitable functionals for the geometry optimization of phosphorus compounds are PBE0, B97-2, and M06-2X. These three have the lowest geometry factor effect on the calculated *σ*(^31^P).

**Figure 17:**
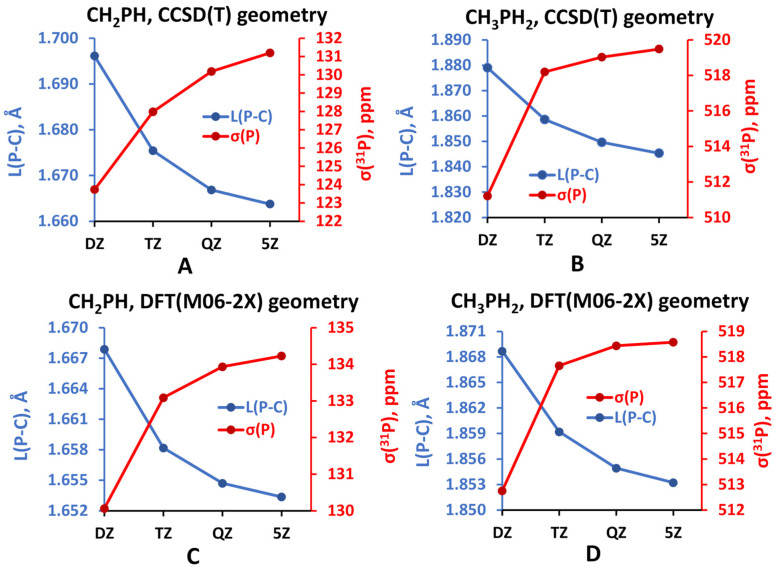
Changes in the phosphorus–carbon bond lengths, L(P–C), and *σ*(^31^P) in CH_2_PH and CH_3_PH_2_ molecules upon varying the quality of Dunning’s basis set cc-pVXZ (X = D, T, Q, 5) used in the geometry optimization procedure carried out within either the CCSD(T) [graphs (**A**,**B**)] or DFT(M06-2X) [graphs (**C**,**D**)] method. The *σ*(^31^P) values were calculated at the GIAO-CCSD(T)/pecS-2 level. Reproduced from Rusakov et al. [199] with the permission of AIP Publishing.

In paper [199], the authors also analyzed how the level of valence splitting of the basis set used at the geometry optimization stage can affect phosphorus–carbon bond lengths and how this influences the *σ*(^31^P) calculated upon these geometries. Figure 17 shows the behaviors of phosphorus–carbon bond lengths L(P–C) (left axis) and *σ*(^31^P) (right axis) in molecules CH_2_PH and CH_3_PH_2_ upon varying the cardinal number of the Dunning’s basis set cc-pVXZ (X = D, T, Q, 5) used at the geometry optimization stage, performed at the CCSD(T) and DFT(M06-2X) levels of theory (see plots A, B and C, D, respectively).

It can be seen in Figure 17 that the P–C bonds are essentially contracted by the improvements of the basis sets used in the geometry optimization procedure for both methods of optimization. This corroborates the finding of Helgaker et al. [198] who has also noticed that, in general, bond lengths are contracted by the improvements of the basis sets used in the geometry optimization procedure. The contraction of the P–C bond lengths in hundredths of Å in moving from the double- to pentuple-zeta level of the basis set quality resulted in changes in σ(^31^P) of 6–7 ppm. This speaks in favor of the fact that the geometry factor effect can be comparable in magnitude with the solvent and vibrational corrections, and that it can be effectively reduced by means of the proper choice of basis sets used at the geometry optimization stage.

In light of the importance of the basis set issue in reducing the geometry factor effect, new, efficient pecG-*n* (*n* = 1, 2) basis sets for the geometry optimization of molecules containing hydrogen and p-elements of the second to fourth periods were presented [199,200,201]. In these works, the optimization of basis set functions was carried out by means of the PEC method [172], with the target function representing a molecular energy gradient relative to the bond lengths of the selected bonds that involve particular atoms. This can be rationalized as a random but constrained search of the set of exponents that results in bond lengths as close to the ideal equilibrium values as possible, provided that the optimized exponents give the lowest possible molecular energy.

**Figure 18:**
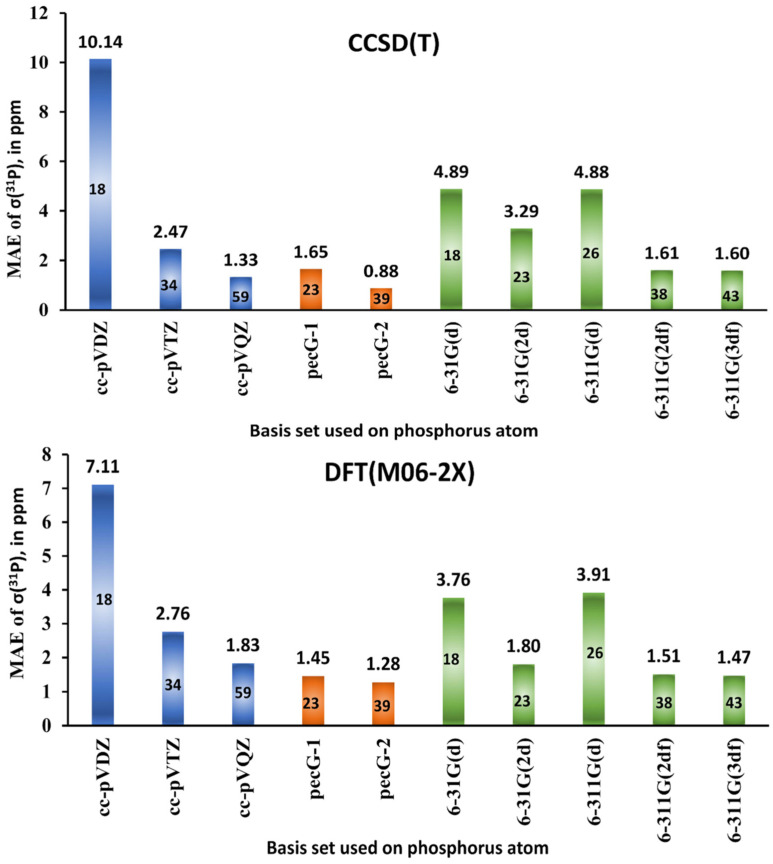
The MAEs (in ppm) of the theoretical values of σ(^31^P) of various phosphorus-containing molecules calculated at the GIAO-CCSD(T)/pecS-2 level of theory at the equilibrium geometries obtained within the CCSD(T) (**top diagram**) and DFT(M06-2X) (**bottom diagram**) methods using different basis sets against the values of σ(^31^P) calculated at the reference CCSD(T)/cc-pV5Z equilibrium geometries. The numbers within the bars represent the sizes of the basis sets. Reproduced from Rusakov et al. [199] with the permission of AIP Publishing.

The effect of using various basis sets, including the pecG-*n* (*n* = 1, 2), in the geometry optimization of phosphorus compounds on the calculated *σ*(^31^P) is shown in Figure 18. This figure reflects the MAEs of the *σ*(^31^P) calculated at the GIAO-CCSD(T)/pecS-2 level of theory on the CCSD(T) and DFT(M06-2X) equilibrium geometries obtained with different basis sets, against theoretical reference values of *σ*(^31^P) calculated at the same levels of theory but on the CCSD(T)/cc-pV5Z geometry.

In Figure 18, one can see that the pecG-*n* (*n* = 1, 2) basis sets perform very well in reducing the geometry factor effect, as the MAEs are considerably smaller than those provided by Pople and Dunning’s commensurate basis sets. However, caution should be practiced when these calculations are aimed at the direct comparison of the calculated geometrical parameters with the electron diffraction experiment. In fact, the bond lengths and bond angles calculated with the pecG-*n* (*n* = 1, 2) basis sets can be misleading in this situation, since they represent equilibrium parameters, while the experimental values inevitably include vibrational averaging.

It is also worth paying attention to a specific question concerning the addition of the diffuse functions to the basis sets used in geometry optimization of phosphorus compounds. The diffuse functions are the extended basis functions with very small exponents that provide the flexibility of description of the portions of the molecular orbitals located far away from the nucleus (the so-called “orbital tails”). The need for such functions arises in the case of the presence of weakly bound electrons or when the property of interest depends on the wave function tails. For instance, in negatively charged systems, excess negative charge spreads out from nucleus to distant regions, and diffuse functions are required to properly describe the electron density in those regions. In addition, diffuse functions are often crucial in the description of noncovalent interactions, such as hydrogen bonding, which is responsible for the supramolecular ordering in biological systems. In this respect, one might wonder whether they can also be important for the accurate quantum chemical calculations of the systems containing atoms with lone electron pairs, such as phosphorus. The answer to this question can be found in ref. [199], where it was clearly shown that the inclusion of the additional diffuse functions into the basis sets used at the geometry optimization stage either noticeably deteriorates the accuracy of the calculated *σ*(^31^P) or does not alter it at all. This statement pertains to ordinary phosphorus compounds, i.e., those which do not represent specific situations, like anions or noncovalent interactions. Given that additional diffuse functions considerably enlarge computational costs, the geometry optimization of ordinary phosphorus compounds can be recommended to perform without them.

Concluding this section, it is worth saying that ^31^P NMR shielding constants are indeed sensitive to the quality of the equilibrium geometry on which they are calculated. The geometry factor effect on phosphorus shielding constants can reach substantial magnitudes, up to 10 ppm, depending on the method/basis set used in the geometry optimization of phosphorus compounds, while in some extraordinary cases, such as phosphorus mononitride, this influence can be even larger, up to 20 ppm. At the same time, the geometry factor effect on phosphorus chemical shifts is less pronounced due to the partial cancelation of the geometry factor errors of standard and sample compounds.

## 6. Solvent Effects on ^31^P NMR Shielding Constants/Chemical Shifts

^31^P NMR chemical shifts are known to be quite sensitive to effects of media or solvent effects [202,203,204,205]. The molecules of solvents can considerably modify the electron density of phosphorus-containing molecules; therefore, taking into account solvent effects via different computational models is crucial for accurate predictions of ^31^P NMR chemical shifts, especially when specific solute–solvent noncovalent interactions are involved [206].

In general, the effects of a solvent on nuclear shielding constants can also be divided into four different types [207]: (1) the change in the local magnetic field experienced by the nucleus due to the isotropic magnetizability of the solvent molecules, which is proportional to the magnetizability of the solvent; (2) a change in the local magnetic field due to the magnetizability anisotropy of solvent molecules in the close vicinity; (3) the change in the electronic structure of the solute due to van der Waals interactions with solvent molecules; and (4) the contribution from the electrostatic polarization of the solute’s charge distribution.

The influence of the solvent’s effects on phosphorus NMR chemical shifts can be implemented within the framework of two conceptually different classes of models in accordance with the microscopic description of the solvent. The first class comprises the continuum (or implicit) models, in which the solvent is considered to be a structureless continuum characterized by its bulk properties. The second class comprises the discrete (or explicit) models that explicitly treat the degrees of freedom of solvent, fully or partially.

The most popular continuum model is the polarizable continuum model (PCM) [208,209,210] and its advanced version, the integral equation formalism, the IEF-PCM [211,212,213,214,215]. In the polarizable continuum model, the solvent is represented by a homogeneous continuum medium, which is polarized by the solute placed in the cavity built in the dielectric medium. From basic electrostatics, it is known that the response of a homogeneous dielectric continuum to any charge distribution of the solute produces the charge distribution on the cavity surface, arising from the polarization of the dielectric medium. For arbitrarily shaped surfaces, induced charge distribution cannot be determined by analytical means, and different numerical approaches are needed. For spherical and ellipsoidal cavities, the screening charge density can be found analytically, in particular, within the Onsager model [216]. Computational modeling of the solvent effect on NMR molecular parameters by the polarizable continuum model was thoroughly reviewed by Cammi, Mennucci, and Tomasi [217]. In general, the IEF-PCM is the most popular model used in the calculations of ^31^P chemical shifts today [132,136,141,156,218,219], as it is readily available in many quantum–chemical packages and does not require considerable computational resources, no matter what stage it is applied at, let it be the geometry optimization stage or the NMR calculations.

Moreover, the formulation of the four-component relativistic Dirac–Hartree–Fock and Dirac–Kohn–Sham theories for a molecular solute described within the framework of the polarizable continuum model was recently presented by Di Remigio et al. [220]. The linear response function for the four-component PCM-SCF state was derived, enabling the four-component calculations of the NMR parameters, by taking into account the solvent effects within the PCM. The algorithm was implemented into the DIRAC program package [221].

Another popular model of accounting for the solvent effects, being a representative of continuum-type models, is the conductor-like screening model, the COSMO [222,223,224,225]. This is an approximate, but very accurate, non-iterative approach for the solution of equation on the screening charge density for arbitrarily shaped cavities. Like the IEF-PCM, the COSMO is also based on the surface segmentation of a molecule surface. Within this model, the dielectric screening charges and energies are calculated on van der Waals-like molecular surfaces in the approximation of a solvent represented as an ideal conductor (the dielectric permittivity set to infinity). The COSMO model has received some attention in the calculations of ^31^P chemical shifts lately [142,226,227]; however, the greatest interest is aroused today by the other models, namely, by those which take into account specific solute–solvent interactions explicitly. Indeed, the reaction field or continuum models provide an effective description of long-range electrostatic interactions, though, when it comes to specific short-range interactions they can fail, and discrete models can be quite effective in such kind of problems, of course, on the condition that they are applied with due knowledge, including proper adjustment of the solvent molecules around the solute [228].

One of the most direct discrete models is the supermolecule solvation model (SSM). It treats the solute molecules in the surroundings of a number of explicitly treated solvent molecules. The SSM is very demanding with respect to computational resources, although it may be of particular interest for heteroaromatic phosphorus-containing compounds for which spectral data have been received in aromatic or in polar solvents. In the calculations of *σ*(^31^P)/*δ*(^31^P), the SSM is frequently combined with the PCM [229,230,231].

An example of using the SSM in combination with PCM in the calculations *δ*(^31^P) was presented by Rusakov et al. [231]. In that paper, a number of computational schemes for the calculation of *δ*(^31^P) were examined on a series of azoles, phospholes, and phosphazoles. It was found that solvent, vibrational, and relativistic corrections are basically of the same order of magnitude and alternate in sign, being, on average, of about 2–3 ppm in absolute value. However, introducing the solvent molecules into the computational space brought about much larger solvent corrections, up to 14 ppm, depending on the solute molecule under consideration.

**Figure 19:**
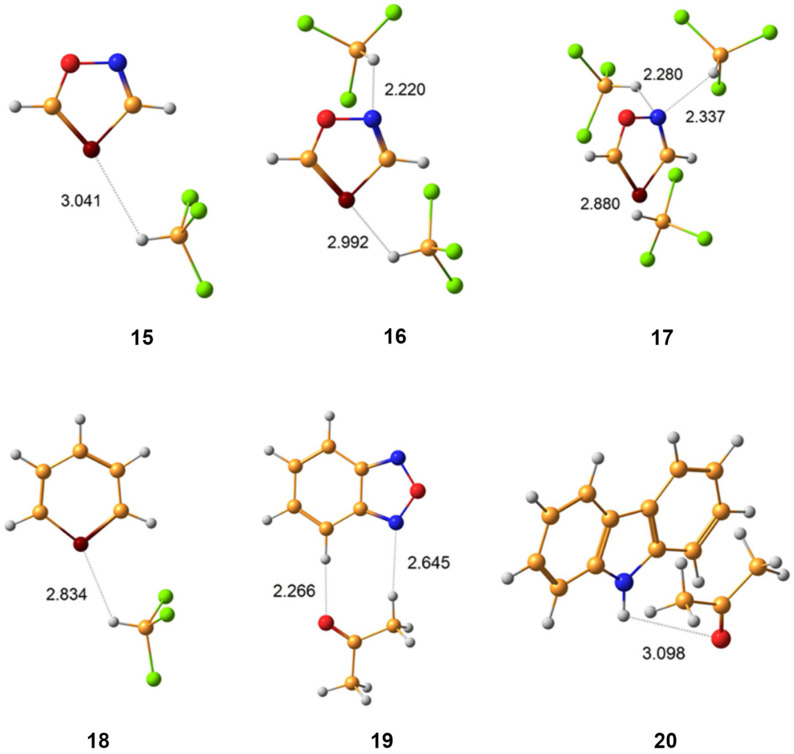
Supermolecular solvation complexes of 1,2,4-oxazaphosphole with one, two, and three molecules of chloroform (respectively, **15**, **16**, and **17**), phosphinine with one molecule of chloroform (**18**), benzo[c][1,2,5]oxadiazole with one molecule of acetone (**19**), and 9H-carbazole with one molecule of acetone (**20**). Element colors: carbon—yellow, hydrogen—gray, nitrogen—blue, oxygen—red, phosphorus—brown, and chlorine—green. All interatomic distances are given in Å. Reproduced from Rusakov et al. [231] with permission from the American Chemical Society (Washington, DC, USA).

Thus, in the SSM calculations performed by Rusakov et al., one to three molecules of the solvent (chloroform and acetone) were added to the computational space of the solute molecule, implying the description of bulk solvent effects within the IEF-PCM formalism. The implementation of the explicit solvent molecules was made at both stages of calculation, the geometry optimization stage and the NMR calculation stage. The considered supermolecular solute–solvent complexes are illustrated in Figure 19.

From the results obtained by Rusakov et al. it follows that, in general, the SSM approach noticeably improves the calculated values of chemical shifts compared to the experiment. As an example, adding one, two, and three molecules of chloroform into the computational space of 1,2,4-oxazaphosphole (complexes **15**, **16**, and **17**) increased the IEF-PCM value of 71.7 ppm to 76.9, 82.0, and 85.7 ppm, respectively, as compared to the experimental value of 84.0 ppm.

Another effective approach consists in the exploitation of explicit and continuum solvation models at different stages of calculation. Such a dual model was considered by Maryasin and Zipse [123], who applied this in the calculations of *δ*(^31^P) of phosphanes and related compounds in a solution. They selected two ways to calculate the phosphorus chemical shifts: (a) the use of the PCM in both geometry optimization and *δ*(^31^P) calculations (solution model 1); and (b) the inclusion of one to three explicit solvent molecules in the geometry optimization performed within the PCM and subsequent *δ*(^31^P) calculations on this solvent/solute complex using the PCM continuum solvation model at the stage of NMR shift calculations (solution model 2). It was found that *δ*(^31^P) values in solutions are predicted more accurately when using solution model 2. They also showed that the addition of only one solvent molecule at specific user-selected positions in combination with the PCM leads to a good reproduction of the *δ*(^31^P) of triphenylphosphine oxide in chloroform with triphenylphosphine being the reference.

Another development of solvation models consists in combining quantum mechanics (QM) and molecular mechanics (MM) methodologies (QM/MM) [232,233,234,235]. This approach considers the chemically important part of the system within a quantum mechanical method, while the rest is treated with standard molecular mechanics, using a molecular force field (FF). Most QM/MM methods describe interactions between the QM molecular system and the environment using either the simple mechanical embedding scheme or the more accurate electrostatic embedding [236]. In this respect, the ONIOM approach (our own N-layered integrated molecular orbital and molecular mechanics) [237,238,239,240,241] has become a powerful computational method that divides large molecules into multiple layers, treating each layer with a different level of theory (e.g., high-level QM for the active site and lower-level QM or classical MM for the rest) to accurately and efficiently model complex systems, using a subtractive energy formula for reliable results. In general, the QM/MM methods are now the most studied tools for the prediction of phosphorus chemical shifts made by accounting for media effects, as they allow us to considerably improve the precision of ^31^P NMR spectra simulations [242,243].

Within the SSM or QM/MM approaches, it is possible to perform thermodynamic averaging by a molecular dynamical (MD) or random Monte Carlo (MC) sampling of the relevant states. In particular, Přecechtělová et al. [244,245] and Fukal at al. [138,139,246] highlighted the importance of MD sampling in the conformational averaging of phosphorus-containing compounds when explicit solvation is considered, employing classical molecular dynamics MD/DFT protocols. However, such calculations are very time-consuming, even on the most powerful computers, but they may benefit considerably from parallel computing [247] in the near future.

**Figure 20:**
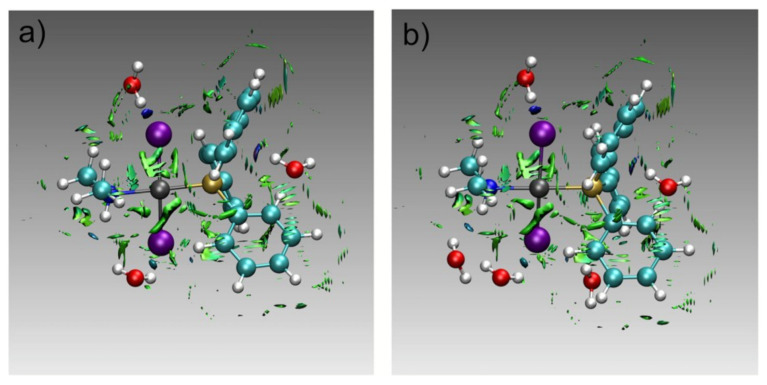
The snapshots of the [PtCl_2_(dma)(PR_3_)] complex with (**a**) 3 and (**b**) 5 explicit water molecules, identified based on the noncovalent interaction regions (highlighted in blue/green). Reproduced from Castro et al. [206] with the permission of the Royal Society of Chemistry.

For example, Castro et al. [206] reported on the MD simulations of *δ*(^31^P) calculations of trans-platinum(II) complexes. The authors performed the calculations within the two- and four-component GIAO-DFT method, namely, ZORA-DFT and mDKS, respectively. They explicitly considered the solvent molecules, varying their number in the first coordination sphere from three to five. Figure 20 demonstrates the [PtCl_2_(dma)(PR_3_)] complex with three (Figure 20a) and five (Figure 20b) explicit water molecules, with noncovalent interaction regions shown in blue/green.

Having decided on the starting positions of water molecules that need to be taken into account in the first coordination sphere for the case of the [PtCl_2_(dma)(PR_3_)] complex, Castro et al. studied the importance of molecular dynamics for the description of solvent effects on *δ*(^31^P) with the aid of ab initio molecular dynamics (AIMD) simulations, where phosphine (the reference molecule) and the [PtCl_2_(dma)(PR_3_)] complex were surrounded by solvent water molecules, whereupon their evolution over time was monitored. From the obtained trajectory, thirty snapshots were taken in total at regular intervals to estimate the dynamically averaged *δ*(^31^P). The findings of Castro et al. suggested that the inclusion of dynamic effects is of crucial importance for accurate description of *δ*(^31^P) in trans-platinum(II) complexes. Indeed, based on their results obtained with static and dynamic calculations, it can be concluded that the effect of dynamical averaging reaches up to 5 ppm as compared to the static results for the ZORA-DFT method, and up to 9.5 ppm for the mDKS method, depending on the DFT functional used.

**Figure 21:**
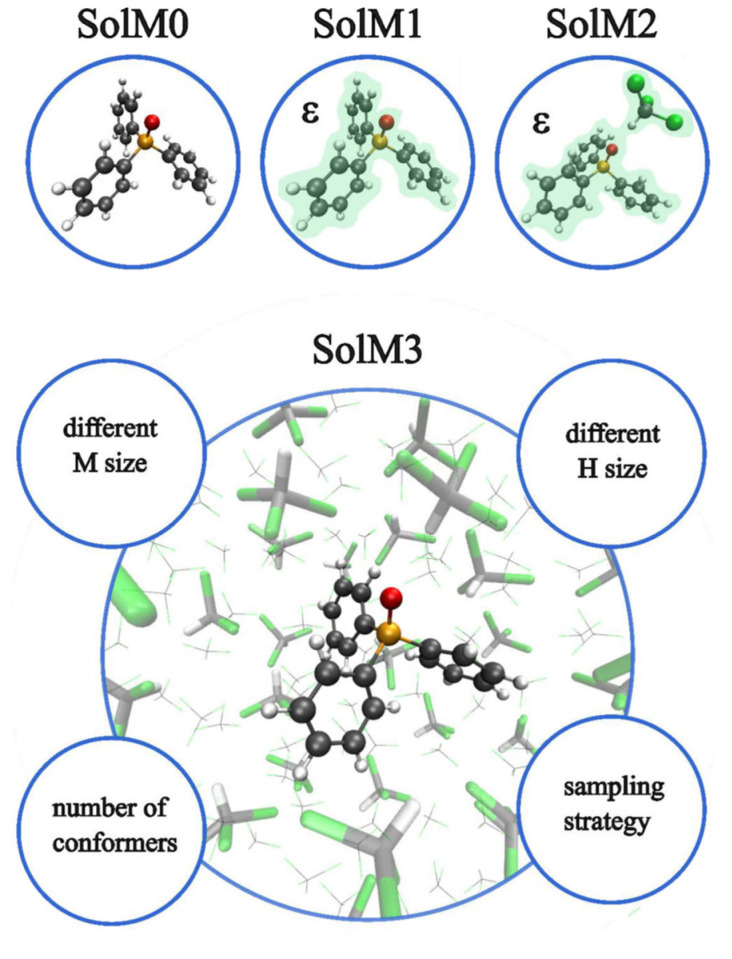
Various solvation models that were applied by Calgano et al. in the calculations of ^31^P NMR chemical shifts in triphenylphosphine oxide and triphenylphosphine placed in chloroform. Reproduced from Calgano et al. [243] with the permission of John Wiley & Sons, Inc.

Calgano et al. [243] also studied the performance of various solvation models in the calculations of *δ*(^31^P) of triphenylphosphine oxide and triphenylphosphine placed in chloroform. The authors used MD simulations and hybrid QM/MM calculations to investigate the effects of solute/solvent interactions and, more generally, to estimate the effect of the embedding in the NMR simulations within the COBRAMM algorithm that was developed by Garavelli’s group [248,249]. The studied solvation models included solution model 0 (SolM0), which does not include any solvation, solution model 1 (SolM1), which involves implicit solvation via PCM, solution model 2 (SolM2), which assumes micro-solvation when one molecule of solvent is added to implicit PCM solvation, and solution model 3 (SolM3), which embodies the explicit solvation that treats high (H), medium (M), and low (L) layers differently in the QM/MM scheme in the COBRAMM code. In SolM3, the M and the L layers were treated at the MM level, while the H-layer was treated at the QM level. These solvation models are depicted in Figure 21. The structural components of the H, M, and L-layers are shown with balls and sticks, thick sticks, and thin sticks, accordingly, as they are considered in the QM/MM scheme.

Different solvation models were studied along with the MD and QM/MM computational protocols. Classical MD simulations are crucial for capturing sufficiently big ensembles of solute/solvent conformations, mimicking the variety of possible electrostatic and van der Waals interactions in solute/solvent clusters, but classical MD alone does not lead to reliable geometries for the calculation of *σ*(^31^P) values. Calgano et al. has found that the latter can be substantially improved by means of QM/MM optimizations (with electrostatic embedding) carried out on top of the geometries extracted from the MD trajectories under the condition that a sufficiently large solvent shell is treated at the QM level, while the rest of the solvent is treated with the implicit PCM. In general, they found that the QM/MM geometry optimizations improve the convergence of the phosphorus shielding values, reducing the number of geometries extracted from MD trajectories to be considered in the NMR computations.

**Figure 22:**
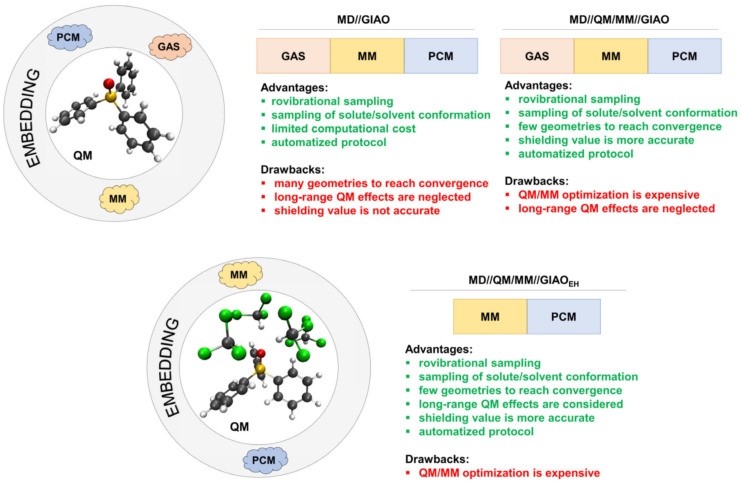
Advantages and drawbacks of different solvent description levels in the ^31^P NMR chemical shift calculations considered by Calgano et al., including the MD//GIAO, MD//QM/MM//GIAO, and MD//QM/MM//GIAO_EH_ protocols. Reproduced from Calgano et al. [243] with the permission of John Wiley & Sons, Inc.

The authors established the following facts: (a) the direct MD//GIAO protocol (NMR calculation is performed directly on top of the MD geometries) is not very computationally expensive but requires the sampling of many geometries to achieve converged values of shielding constants; (b) the MD//QM/MM//GIAO protocol (the NMR calculation is performed after the QM/MM geometry selection of MD snapshots) brings both the reduction in the number of sampled geometries needed for NMR calculation and a significant improvement of the convergence of the NMR chemical shift value in relation to the number of sequential snapshots; and (c) the MD//QM/MM//GIAO_EH_ protocol provides further improvement. The latter protocol assumes four ingredients: (1) the averaging of the shielding value over the sampling of multiple solute/solvent clusters through MD simulations; (2) refining the quality of the geometry of the MD samples using the QM/MM optimizations; (3) including an increasing number of solvent molecules until reaching convergence to take into account the long-range QM effects from the environment on the shielding value; and (4) approximating the bulk effect of the solvent on the solute/solvent clusters via the implicit PCM. Figure 22 shows the advantages and drawbacks of the different computational protocols proposed by Calgano et al. for the *σ*(^31^P) calculations.

In conclusion of this section, it should be noted that solvent corrections to ^31^P NMR chemical shifts can be either very small or very large in magnitude, varying in a wide range from ca. −20 to +20 ppm. They typically provide the correction to the main value of 3–5% of the total value. In this respect, a great variety of different computational models for treating the solvent effects are available now, and they should be considered very carefully, taking into account the specifics of the system in question.

## 7. Relativistic Effects on ^31^P NMR Shielding Constants/Chemical Shifts

Relativistic effects are crucial in the calculations of phosphorus chemical shifts for molecules containing heavy elements starting from 3rd period and below in the periodic table. A great effort was invested in understanding the peculiarities of relativistic effects on phosphorus chemical shifts, especially in relation to the ^31^P NMR spectra simulation of heavy transition metal complexes with phosphorus ligands [190,191,206] or phosphines and phosphine chalcogenides with heavy substituents [126,250,251].

In general, phosphorus per se can be thought of as a moderately heavy nucleus of the 3rd period, manifesting relativistic effects on its own NMR shielding. This is called the heavy atom (HA) effect on the nuclear shielding of a heavy atom (HA) itself (HAHA). The HAHA effect on *σ*(^31^P) is known to provide a constant contribution of about 15–20 ppm [133,156,163,252], depending on the quantum chemical method and the basis set used for its calculation.

At the same time, when other heavy atoms of the 3rd period and beyond are present in phosphorus compounds, their relativistic influence on *σ*(^31^P) is called the heavy atom (HA) effect on the nuclear shielding of a light atom (LA) (HALA). The HALA effect was coined more than 50 years ago by Nomura, Takeuchi, and Nakagawa [253] when studying anomalous magnitudes of the chemical shifts in the ortho- and meta-protons of monosubstituted halobenzenes via the so-called “spin polarization shift.” Until now, various mechanisms of the relativistic HALA effect on chemical shifts in various nuclei have been extensively investigated [254,255,256,257,258]. By analogy to the other nuclei, the most pronounced HALA effect experienced by phosphorus nuclei should be due to the relativistic effects coming from neighbor heavy atoms separated from phosphorus by one bond (α-HALA effects), while more distant heavy atoms, in general, impose less pronounced influence (the so-called β-, γ-, etc. HALA effects), though, it should be noted that for the other NMR-active nuclei, there are cases when β- and γ-HALA effects are significant and comparable to the α-HALA in magnitude [259,260]. In the case of *σ*(^31^P), all studies so far have only concerned the α-HALA effect, while the peculiarities of the distant HALA effect are yet to be investigated.

The HALA effect is extremely sensitive to the electronic structure of both HAs and LAs and to the character of interactions between them [254,255]. Therefore, in different phosphorus compounds containing heavy atoms, the relativistic HALA effect on phosphorus shielding constants may vary in a very wide range, causing the total relativistic effect (HAHA + HALA) to be also substantially different. For example, in phosphine oxides, sulfides, selenides, and tellurides with light alkyl substituents, the total contribution of relativistic effects amounts to 3–5, 5–7, 12–17, and 14–30% of the total value of *σ*(^31^P), respectively [250,251]. In the *δ*(^31^P) scale, relativistic effects can also have substantial contributions, sometimes reaching tens [230,261,262] or even hundreds of ppm, such as in the case of N-vinylimidazole with phosphorous pentachloride complexes [263]. Given such a wide range of relativistic corrections to *δ*(^31^P) in various phosphorus compounds, as well as their extraordinary high magnitudes in some cases, it can be said that relativistic effects on *σ*(^31^P)/*δ*(^31^P) are a matter of no less importance than solvent or vibrational corrections, and they deserve special consideration.

According to Fukui et al. [264], the relativistic effects of different types on NMR shielding tensors can be derived in a convenient mathematical form using the two-component positive energy Hamiltonian ${\hat{H}}_{+}$ for a molecule being placed in an external magnetic flux density **B**. For the sake of brevity, omitting the two-electron part, the one-electron positive Hamiltonian ${\hat{H}}_{+}$ (${\hat{H}}_{1e}$) is expressed as follows:

(12) $${\hat{H}}_{1e}=\sum_{i}[-\sum_{L}\frac{Z_{L}}{r_{iL}}+\frac{1}{2}\left({\hat{\pi }}_{i}^{2}+i{\hat{\sigma }}_{i}\cdot \left[{\hat{\pi }}_{i}\times {\hat{\pi }}_{i}\right]\right)-\frac{1}{8}\alpha^{2}{\left({\hat{\pi }}_{i}^{2}+i\sigma_{i}\cdot \left[{\hat{\pi }}_{i}\times {\hat{\pi }}_{i}\right]\right)}^{2}+\;\frac{1}{4}\alpha^{2}\sum_{L}Z_{L}\frac{{\hat{\sigma }}_{i}\cdot \left[r_{iL}\times {\hat{\pi }}_{i}\right]}{r_{iL}^{3}}+\frac{1}{2}\pi \alpha^{2}\sum_{L}Z_{L}\delta (r_{iL})],$$

In Equation (12), $r_{iL}=r_{i}-R_{L}$ is the radius-vector of *i*-th electron ($r_{i}$) in relation to the position of nucleus *L* ($R_{L}$), *Z_L_* is the charge of nucleus *L*, ${\hat{\sigma }}_{i}=2{\hat{s}}_{i}$ embodies the electron spin operator, and ${\hat{\pi }}_{i}={\hat{p}}_{i}+A_{0}+\sum_{N}A_{N}$ is the field-dependent momentum operator in minimal substitution notation, with $A_{0}=\frac{1}{2}\left[B_{0}\times r_{iO}\right]$ and $A_{N}={\;\alpha }^{2}\gamma_{L}\frac{\left[I_{N}\times r_{iN}\right]}{r_{iN}^{3}}$ representing the vector-potential of the external magnetic field $B_{0}$ and that of the point-like magnetic nuclear dipoles with nuclear magnetic moments $\mu_{N}=\gamma_{N}I_{N}$, respectively. The parameter *α* represents the fine structure constant, which is reciprocal to the speed of light and determines the smallness of various contributions, and $\gamma_{L}$ is the gyromagnetic ratio for nucleus *L*. The SI system of atomic units (*e* = *m* = ℏ = 1 and *c* = *α*^−1^ = 137.0359998) is used.

The first two terms in Equation (12) correspond to the nonrelativistic part ${\hat{H}}_{1e}^{nrel}$, which gives rise to magnetically unperturbed operators of one-electron kinetic and potential energy and all standard hyperfine magnetic operators that bear the dependence on either the external or nuclear magnetic field. The magnetically perturbed nonrelativistic terms include (electron) orbital-Zeeman ${\hat{H}}_{B_{0}}^{OZ}$; (electron) spin-Zeeman ${\hat{H}}_{B_{0}}^{SZ}$; paramagnetic (nuclear) spin-(electron) orbit ${\hat{H}}_{N}^{PSO}$; diamagnetic (nuclear) spin-(electron) orbit ${\hat{H}}_{N;B_{0}}^{DSO}$; Fermi-contact ${\hat{H}}_{N}^{FC}$; and spin–dipole ${\hat{H}}_{N}^{SD}$ operators. The relativistic terms appear from the third, fourth, and fifth terms of Equation (12). They are many, though, generally, they are divided into active (with field-dependence) and passive (without field-dependence) operators [265,266]. Among the passive terms, the most important are the two scalar operators, the mass-velocity ${\hat{H}}^{mv}$ and Darwin ${\hat{H}}^{Dar}$ [267], and one (non-scalar) spin–orbit operator ${\hat{H}}^{SO}$ [268,269].

The mass-velocity effect embodies the increase in electron mass when it moves with significant velocity, close to the speed of light. The Darwin interaction is also a very interesting phenomenon that consists of the so-called *zitterbewegung* motion, when the electron coordinates start to fluctuate over the distance $\delta r≅1/m_{e}$ as a result of smearing of the Coulomb potential [270]. The one-electron passive spin–orbit interaction, ${\hat{H}}^{SO}$, follows from the fourth term of Equation (12) and arises due to the interaction of the electron spin with its own orbital angular momentum. The active terms include, in particular, the kinetic energy corrections to the orbital- and spin-Zeeman terms, the ${\hat{H}}_{B_{0}}^{OZ-KE}$ and ${\hat{H}}_{B_{0}}^{SZ-KE}$, and many others [266]. There are indeed a great many types of relativistic effects, and we can recommend many excellent reviews on this topic [256,271,272,273,274,275,276].

The nuclear shielding tensor, $\sigma_{N;\alpha \beta }$ (*α*, *β* = *x*, *y*, *z*), represents the energy derivative with respect to the external magnetic flux density component $B_{\alpha }$ and nuclear magnetic moment component $\mu_{N;\beta }$ (see Equation (3)). Therefore, representing the energy and wave function of a system as power series in terms of multiple perturbation theory, with various hyperfine magnetic and relativistic operators being the perturbations, one can derive relativistic corrections to the shielding tensor directly. If there are no relativistic terms included in Hamiltonian, the second-order perturbation theory will result in standard nonrelativistic expression for the shielding tensor which is the sum of diamagnetic and paramagnetic contributions. If relativistic terms are included in Hamiltonian and taken into account on the same footing as the usual magnetic hyperfine perturbation operators, one arrives at a variety of relativistic corrections to the NMR shielding tensor [266,267,277]. This framework is called as the direct perturbation theory (DPT) [278] or Breit–Pauli perturbation theory (BPPT approach) [133,267,279]. The relativistic corrections to the shielding tensor can also be divided into active and passive types, like the terms in Hamiltonian in Equation (12); however, they more often than not are separated by their dependence on the electron spin operator. Thus, the scalar relativistic corrections imply the fact that they do not include the operators that depend on the electron spin. These contain, in particular, the mass-velocity or Darwin operator. Meanwhile, the spin–orbit corrections are those that contain the spin–orbit operator (which is dependent on the electron spin). At the same time, it should be emphasized that there is also the other type of terms, which cannot be classified as being of the scalar or SO type; such terms may be spin-dependent, but do not necessarily require SO operators in their derivation. Thus, the categorization based on the absence or presence of the operators containing electron spin seems more astute, and all relativistic corrections to the NMR shielding tensor can clearly be separated into spin-free or spin-dependent types.

The most pronounced relativistic effects of the HAHA type come from the cross terms between the relativistic kinetic energy (KE) correction to differenthyperfine operators and the usual magnetic operators entering the nonrelativistic diamagnetic and paramagnetic contributions. The largest individual contribution to the HAHA effect is the second-order active spin-dependent cross term between KE correction to the spin-Zeeman operator (SZ-KE) and the Fermi-contact (FC) operator, the so-called FC/SZ-KE [266,280]. The other active spin-dependent contributions, such as the first-order KE correction to diamagnetic term (d-KE) and the cross-term between the external field and the contact filed (con), are also important but to a lesser extent than the FC/SZ-KE [266]. Passive relativistic corrections forming the HAHA effect are, for the most part, scalar terms that arise from mass-velocity and Darwin corrections to dia- and paramagnetic terms of the shielding tensor, the so-called d/mv, d/Dar, p/mv, and p/Dar. Apparently, in most cases, Darwin corrections are inferior in magnitude to mass-velocity terms [266].

For the most part of nuclei, the most pronounced part of the relativistic effect that comes from neighbor heavy atoms (HALA effect) is due to the SO-HALA mechanism. The SO-HALA effect has been shown to originate from the passive third-order correction that involves the SO, FC, and orbital-Zeeman (OZ) operators. The SO-HALA effect arises due to the spin–orbit coupling interactions on the HA, resulting in the triplet character admixture to the closed-shell singlet ground-state wave function. In its turn, this induces the electron spin density polarization that spreads out in the whole system, leading to non-negligible spin density near the LA under consideration. This interacts with the magnetic dipole of the LA via the Fermi-contact mechanism changing the LA NMR chemical shift [254,255,256]. However, pertaining the *σ*(^31^P), the scalar relativistic effects originating on the heavy atom can sometimes provide nonevanescent contributions to the total HALA effect. For example, Engesser et al. [252] has calculated the dependence of *σ*(^31^P) in molecules PX_3_ with X = F, Cl, Br, and I, at the two-component level of relativistic theory, and established that scalar-HALA relativistic effects on *σ*(^31^P) are progressively de-shielding along the PX_3_ series, and, if taken into account together with the spin–orbit effects, they facilitate the restoration of a proper experimental trend for phosphorus chemical shifts in this series [281,282].

For now, there are two levels of relativistic theory that can be applied to the calculation of the NMR chemical shifts. The most computationally demanding approaches are based on the four-component level of relativistic theory; they constitute a relativistic “golden standard” that provides highly accurate results, provided that a suitable approximation for the electronic wave function is employed [283]. However, in the four-component formalism, the physically tractable relativistic effects of different types cannot be distinguished in an explicit way.

The pioneering works of Pyper [284,285,286,287], Pyykkö [288,289], Zhang and Webb [290], and Quiney et al. [291,292,293] laid the foundation of modern relativistic four-component random phase approximation (RPA), the so-called 4RPA, or a coupled perturbated Dirac–Hartree–Fock (CP-DHF) method for shielding constants. In this respect, Aucar et al. proposed an accomplished full four-component polarization propagator theory (4c-PPT) for nuclear shielding constants [294,295,296,297,298,299,300,301]. The calculations of nuclear shielding constants within the 4RPA formalism have been presented in a number of papers [297,301,302]; however, this method has not received very much popularity yet.

The four-component relativistic theory combined with the DFT method has received greater attention in the calculations of the NMR shielding constants as compared to the 4RPA method, as the DFT method takes into account the electron correlation effects at moderate computational cost, albeit in a nonsystematic way. Accounting for the correlation effects is crucial for the accurate calculation of NMR shielding constants; however, using the four-component level of theory for their calculation within the correlated approaches represents a significant challenge because of the need of resorting to the four-spinor functional spaces, making all four-component methods many times more computationally demanding as compared to their nonrelativistic analogies. Therefore, the only reasonable alternative to extend the computational methodology to the four-component level and to ensure that this model would be applicable in practice was to adopt the DFT formalism to the four-component level.

The most general relativistic Dirac–Kohn–Sham (DKH) or four-component density functional theory (4DFT) was proposed for the first time by Komorovsky et al. [303,304]. Their theory is based on the matrix formulation of the Dirac–Kohn–Sham (mDKS) method and on the use of the restricted magnetic balance (RMB) condition for the small molecular spinor components (the mDKS-RMB methodology). At the present moment, their methodology can be regarded as one of the most efficient approaches to the calculation of the NMR parameters within the four-component DFT method. Apart from the mDKS-RMB methodology, Xiao et al. [305,306] proposed another approach, which is called the orbital decomposition approach (ODA). The ODA also goes beyond the kinetic balance and treats the magnetic part of the response of the small components. However, the ODA deals with more complicated equations than in the DKH method, introducing the basis set only at the stage when the response equations are written via the basis for the large component only. It can be considered as advantageous when one deals with the large component basis set only, but the cost of this advantage is that the equations within the ODA method are complicated, increasing the time of calculations significantly in practice.

In order to lower the computation costs of the four-component methods, the Dirac–Fock operator was decoupled using different strategies to provide the relativistic two-component theories. At the two-component level, different types of relativistic corrections to the shielding tensor (including those mentioned above) are deduced in an explicit form. Generally, a transition to the two-component theories can be performed by transforming the many-body four-component relativistic Hamiltonian to the block-diagonal form, resulting in the elimination of the small spinor components. The other way that also provides the expression for different types of relativistic corrections lies in a straightforward treatment of the relativistic terms as the perturbations operating on a nonrelativistic reference wave function on the same footing as the magnetic perturbation operators (in the way that was shown in the beginning of this section).

Sun et al. [307] has published a thorough review devoted to the two-component schemes for calculating the NMR parameters, surveying in detail various types of two-component Hamiltonians, both the exact two-component (X2C) and the approximate two-component (A2C). It is generally accepted that X2C schemes imply that the eigenvalues of a given two-component Hamiltonian exactly reproduce the solutions of the four-component analog [276]. Among the most well-known X2Cs are the following schemes: normalized elimination of the small component (NESC) [308,309,310,311,312,313,314,315], the infinite-order Douglas–Kroll–Hess (DKH) [316], and the Barysz–Sadlej–Snijders (BSS), and the infinite-order two-component (IOTC) approach [317,318,319,320]. All other two-component schemes that are theoretically unequal to the original four-component problem are called quasi-relativistic or approximate two-component schemes, A2C. These include the following most well-known approaches: zero-order regular approximation (ZORA) [321,322,323], second-order regular approximation to normalized elimination of the small component (SORA-NESC) [324], and finite-order Douglas–Kroll–Hess (DKH) approximations [325,326,327,328,329,330,331,332]. The two-component relativistic ZORA scheme attained the most popularity in the calculations of NMR properties, especially in the calculations of *σ*(^31^P)/*δ*(^31^P).

The pioneering calculations of relativistic corrections to *σ*(^31^P)/*δ*(^31^P) were mostly carried out at the two-component level of relativistic theory. One of such works was presented by Lantto [133], who considered all possible relativistic contributions to the *σ*(^31^P) of the PH_3_ molecule within the BPPT applied in combination with the DFT(KT2) method and compared the results with those obtained at the four-component DFT level. It was found that the results provided by the two-component BPPT agree well with those calculated at the four-component level. Namely, the total relativistic correction to *σ*(^31^P) in the PH_3_ molecule was estimated as 17.7–18.8 and 16.8–18.5 ppm at the two- and four-component levels, depending on whether the uncontradicted cc-pwCV5Z or specific co-b basis set was used. The authors also analyzed what types of the BPPT relativistic contributions play the most important role in this case. They mentioned that the contributions can be divided into “core” and “shift” types. The former are practically isotropic and insensitive to electron correlation and ligand effects (these can be attributed to HAHA) [333], while the latter type is due to the nearby heavy atoms that cause additional relativistic effects on shielding constants of the nucleus under consideration (these can be attributed to HALA). As expected, it is the “core” corrections that were shown to almost entirely determine the magnitude of the relativistic effect on the *σ*(^31^P) in PH_3_, while the effects of the “shift” type are considerably smaller than those of the “core” type. It was found that the leading “core” contributions are due contact term, the KE, mass-velocity, and Darwin corrections to diamagnetic contribution (con, d/KE, d/mv, and d/Dar, respectively), and, most importantly the FC/SZ-KE term, as the largest among all others. The “shift” contributions were found to be small, but not vanishingly small, namely, the largest among them, the p-KE/OZ and p/mv, were found to be approx. 14 and 9 times smaller as compared to the largest “core” FC/SZ-KE contribution, respectively.

Among the first systematic works presenting the relativistic calculations of ^31^P chemical shifts were the works of Chernyshev et al. [229,230,261,262,263,334], who in the majority of their works used the relativistic two-component ZORA method in combination with the DFT approach, briefly called as the ZORA-DFT.

In the beginning of their continuous study of relativistic effects on *δ*(^31^P), Chernyshev et al. extensively used phosphines and phosphine chalcogenides as model compounds. Interest in the chemistry of phosphines and phosphine chalcogenides has increased considerably since the late 1980s, when the direct reaction of red phosphorus with electrophiles in the presence of superbasic catalysts (Trofimov–Gusarova reaction) was discovered [335,336]. This reaction allowed us to synthesize previously unknown or difficult to access phosphines and phosphine oxides [337] which could be converted into phosphine sulfides and phosphine selenides [338]. Given the fact that phosphines and phosphine chalcogenides find the use in many fields, including catalysts [339,340,341,342], the synthesis of semiconducting nanomaterials [343,344], or even in extraction techniques of noble, rare-earth, and transuranium elements [345,346], studying their physical chemical properties is of utmost importance, including their ^31^P NMR spectra parameters. At the same time, the manifestation of relativistic effects can be very weak as well as very strong in phosphines and phosphine chalcogenides; thus, their study represents a separate branch of investigation.

In this respect, Chernyshev et al. [261] studied relativistic effects in ^31^P NMR chemical shifts in the simplest phosphines, phosphine oxides, phosphine sulfides, and phosphine selenides Me_3_P and Me_3_PX (X = O, S, Se) using the two-component relativistic ZORA-DFT formalism in combination with the relativistically adopted Dunning basis sets that were optimized within the relativistic two-component DKH scheme, cc-pVXZ-DK (X = D, T, Q) [116,347,348,349]. The authors separated the SO-HALA corrections to *δ*(^31^P) in this series of compounds and found that the SO-HALA is small in Me_3_P and Me_3_PO, being less 1 ppm in magnitude, while in Me_3_PS and Me_3_PSe it amounts to 6–9 ppm and 40–50 ppm, respectively, depending on the quality of basis set used. In the latter cases, these figures correspond to about 15–20 and 80–90% of their nonrelativistic values.

Another interesting study by Chernyshev et al. [229] concerned the theoretical and experimental study of trichloro-[2-(1*H*-pyrazol-1-yl)ethenyl]phosphonium hexachlorophosphate(V) and 1,1,1,1-tetrachloro-1*H*-1λ^6^-pyrazolo-[1,2-*a*][1,2,3]diazaphosphol-8-ium-1-ide. In that work, an exceptional role of relativistic effects in the calculation of *δ*(^31^P) was emphasized. As follows from the data presented in that paper, neglect of relativistic effects, primarily of spin–orbital interactions, leads to an appreciable overestimation of the shielding constants, resulting in erroneous interpretation of signals in the ^31^P NMR spectra. Thus, the error coming from neglecting the relativistic effects can reach up to 100 ppm for some isomers of the compounds mentioned above. It was also noted that the magnitude of spin–orbital contribution to phosphorus *σ*(^31^P)/*δ*(^31^P) sharply increases with the total number of chlorine atoms connected to phosphorus.

In the next papers, Chernyshev et al. [230,263] reported on the intermolecular coordination effects on the ^31^P NMR spectra of molecular complexes of tetracoordinated, pentacoordinated, and hexacoordinated N-vinylpyrazoles and intermolecular complexes of N-vinylimidazole and 1-allyl-3,5-dimethylpyrazole with phosphorus pentachloride studied by theoretical and experimental methods. The formation of an intermolecular dative N → P bond was shown to be accompanied by an upfield shift in the phosphorus resonance signal by about 150–200 ppm. At the same time, the contribution of relativistic effects to *δ*(^31^P) was also found to be very substantial, even comparable in magnitude to that of the solvent effects in 1-allyl-3,5-dimethylpyrazole with a phosphorus pentachloride complex, reducing the phosphorus chemical shift by about 210 ppm. Thus, the consideration of both the solvent and relativistic effects was found to be equally important for studying the steric structure and *δ*(^31^P) of such complexes.

The most recent work of Chernyshev et al. [262] was devoted to studying the conformational and relativistic effects on the ^31^P and ^77^Se chemical shifts in selected diphenyl- and bis-(2-phenylethyl)phosphine selenides with different substituents on the phosphorus atom that were analyzed in terms of the ZORA–GIAO–DFT(B1PW91)/TZP approach. The effect of the conformation of phosphine selenides related to internal rotation about the single P–C bond was found to be insignificant, while the SO-HALA effect on the *δ*(^31^P) in the P=Se fragment initiated by a selenium atom in the vicinity was found to be fairly strong (about 25–30 ppm), corroborating the earlier finding [261] about the necessity of including the relativistic spin–orbit effects into account when considering phosphine selenides.

Around the same time when Chernyshev et al. were conducting their research on the relativistic effects on *δ*(^31^P), Antušek et al. [148] estimated their contribution to the *σ*(^31^P) of the AsP molecule. That was one of the first works where the four-component level of relativistic theory was applied. The authors utilized the relativistic four-component GIAO-4RPA and GIAO-DFT(KT2) approaches. Relativistic corrections to *σ*(^31^P) were obtained with the fully uncontracted cc-pVQZ (cc-pVQZunc) basis set. The relativistic corrections to *σ*(^31^P) obtained within the 4RPA and 4DFT(KT2) approaches were found to be considerably different. The former and latter method resulted in the relativistic corrections of +18.44 and −16 ppm, respectively. The basic magnitudes of the *σ*(^31^P) of AsP were also very different, varying from −559.49 to −541.05 for the HF and from −353.66 to −369.66 ppm for DFT method in going from the nonrelativistic to four-component relativistic level of theory. Starting from the work of Antušek et al. [148], who successfully applied the four-component level of relativistic theory, many researchers have also started to use the latter, which was, in particular, facilitated by the development of computer technology.

**Figure 23:**
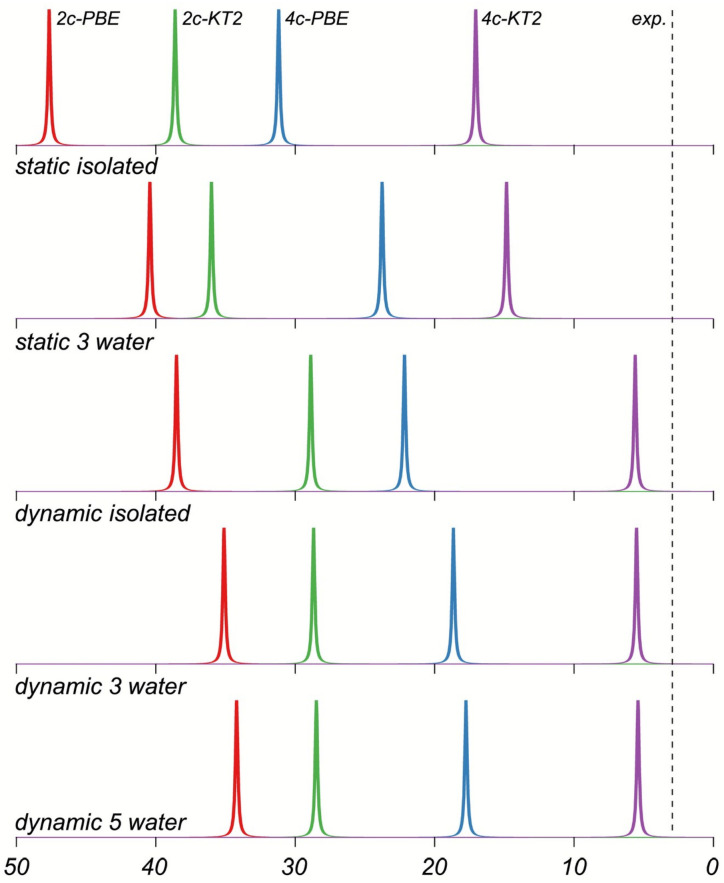
Computed ^31^P NMR chemical shifts of [PtCl_2_(dma)(PR_3_)] calculated within the two-component ZORA-DFT (2c) and four-component mDKS or 4DFT (4c) methods applied with the PBE and KT2 functionals. The two top graphs show the results of static calculations without (*static isolated*) and with 3 water molecules used as solvent (*static 3 water*), while the three bottom graphs correspond to the MD-averaged results obtained without explicit water molecules (*dynamic isolated*), with 3 (*dynamic 3 water*) and 5 explicit water molecules (*dynamic 5 water*), respectively. The experimental value is indicated by a dashed line. Reproduced from Castro et al. [206] with permission from the Royal Society of Chemistry.

In particular, Castro et al. [206] has presented an interesting example of the application of both two-component ZORA-DFT and four-component mDKS approaches, used in combination with KT2 and PBE functionals and dyall.cvtz basis set, to the calculations of *δ*(^31^P) in trans-platinum(II) complexes. The MD-averaged values of *δ*(^31^P) obtained at both levels of relativistic theory were compared with each other and with experiment. It was found that the best match compared to the experiment (with the deviation of only 1 ppm) was provided by the four-component mDKS method applied with the KT2 functional. The ZORA-DFT method was found to have inferior accuracy to the mDKS method. The values of *δ*(^31^P) of [PtCl_2_(dma)(PR_3_)] calculated by Castro et al. [206] with various methods of treating the relativistic and solvent effects are shown in Figure 23.

Fedorov et al. [132] presented the calculations of relativistic corrections to the *δ*(^31^P) of 53 phosphorus-containing compounds, which were performed at the four-component DFT method using the KT2 functional and two types of LDBS schemes with the basis sets applied in the uncontracted form, namely, the 6-311++G(3d,2p)/6-311++G(d,p) and the pcS-3/pcS-2. The heaviest atoms in the considered systems were sulfur and phosphorus per se, so Fedorov et al. mostly dealt with the HAHA effect on *δ*(^31^P), which can be found to vary in the range from −40 to −15 ppm when omitting sulfur-containing compounds. As was shown by Chernyshev et al. [261], sulfur, if located in the vicinity of the phosphorus atom, is capable of providing an additional contribution of about 6–9 ppm in magnitude due to the HALA effect. The results of Fedorov et al. corroborate the fact that the presence of sulfur atoms in close vicinity to phosphorus makes the relativistic effect on *δ*(^31^P) more pronounced, though, the relativistic HALA correction appears to be considerably larger than 6–9 ppm, as the total relativistic corrections in the majority of the compounds containing sulfur–phosphorus bonds were calculated as −85 to −50 ppm.

It is also very interesting to note that the largest relativistic correction was found in molecule Cl_3_PS, provided that there were molecules among the considered ones which contain phosphorus atom surrounded by as many as three sulfur atoms. This finding by Fedorov et al. [132] corroborates the results of Chernyshev et al. [230,263], who also established extremely large relativistic effects on *δ*(^31^P) in intermolecular complexes containing phosphorus tri- or penta-chloride. Evidently, this may be connected with the fact that the SO-HALA effect (which is 90% is responsible for the total HALA effect in the most cases) is strongly determined by the presence of π-type lone electron pairs (LEPs) on heavy atoms, which are the main source of the spin–orbit relativistic effects [350,351]. In contrast to two π-type LEPs on a halogen atom (like chlorine), chalcogen substituents (like sulfur) contain only one π-type LEP; hence, chalcogens must represent substantially weaker generators of the SO-HALA effect than halogens.

Field-Theodore et al. [154] and Kupka et al. [156] calculated the relativistic effects on the *σ*(^31^P) of PF_3_ and PN molecules, respectively, at the four-component DFT level of theory, using the KT2 functional. In both cases, the relativistic corrections to *σ*(^31^P) were estimated as approx. 16.84 ppm for PF_3_ and 11.5 ppm for PN. These are comparable with the typical HAHA effect of the phosphorus atom on its own having a shielding constant of *ca.* 15–20 ppm [133,156,163,252]; thus, it can be concluded that the HALA effect on *σ*(^31^P) in PF_3_ and PN molecules must be small. However, Kupka et al. have mentioned that in relation to the final theoretical value of *σ*(^31^P) of PN, the relativistic correction is abnormally high, reaching as much as ~25% of the total nonrelativistic value [156], while this correction for the other tested molecules was less than 7%. This can be easily explained, because the basic nonrelativistic value of *σ*(^31^P) in PN molecules is about 39–40 ppm [156], which is considerably smaller than that for the other molecules.

**Figure 24:**
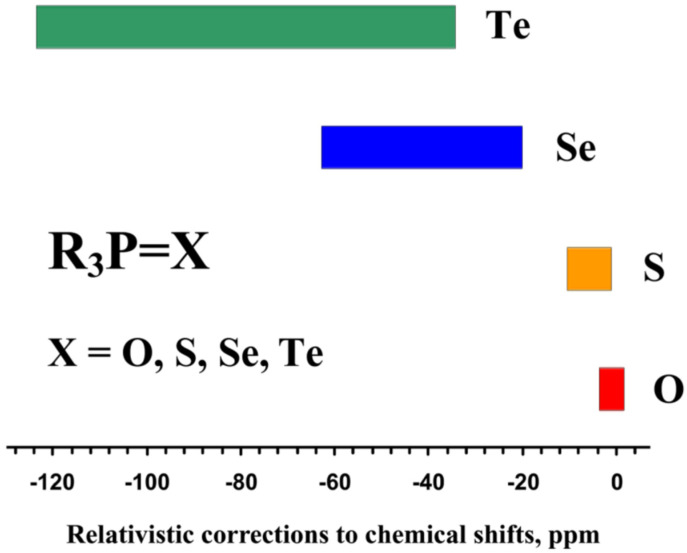
The ranges of relativistic corrections to ^31^P chemical shifts in phosphine oxides, sulfides, selenides, and tellurides. Reproduced from Rusakov et al. [250] with the permission of John Wiley & Sons, Inc.

Rusakov et al. [250] calculated the relativistic corrections to *σ*(^31^P)/*δ*(^31^P) of 56 phosphine chalcogenides of the R_3_PX type (with X = O, S, Se, Te, and R standing for light alkyl substituents) at the four-component relativistic DFT(KT2)/dyall.av3z level. Relativistic corrections to *σ*(^31^P) were found to fall into the range of 3–5% for phosphine oxides, 5–7% for phosphine sulfides, 12–17% for phosphine selenides, and as much as 14–30% for phosphine tellurides in relation to their relativistic values. In contrast to the relativistic corrections to *σ*(^31^P), the relativistic corrections to *δ*(^31^P) were found to be mostly negative in sign and fall in the ranges shown in Figure 24.

Generally, it follows that the nonrelativistic calculations of ^31^P NMR chemical shifts are out of practical use for phosphine selenides and especially for phosphine tellurides, resulting in an enormous overestimation of their values reaching several dozens of parts per million. As an example, the nonrelativistic calculation of ^31^P NMR chemical shift in trimethylphosphine telluride results in the value of about 14.6 ppm, while the relativistic correction is as much as −99.8 ppm!

Rusakov et al. noticed an interesting substitutional trend, which consists of the increasing the de-shielding effect on *δ*(^31^P) upon going to more branched alkyl substituents in all four series, starting from methyl to tertiary butyl groups at phosphorous. At the same time, an increasing inductive +I effect was found to result in the shielding effect when going from almost neutral CH_3_ groups to a powerful σ-donor SiH_3_ group.

The decrease in the relativistic corrections to *σ*(^31^P) (de-shielding effect) in phosphine chalcogenides with branching of alkyl substituents at phosphorus has been studied in more detail and explained in subsequent work by the same authors [251]. Thus, the relativistic HALA effect has been shown to depend on the spatial deformation of the lone electron pairs on heavy atom. This was demonstrated on the example of alkyl and alkene phosphine tellurides. It has been proven that the HALA effect on *σ*(^31^P) is strongly dependent on the spatial arrangements of light substituents on phosphorus because of the deformation of the π-type lone electron pair of tellurium, π-LP(Te).

**Figure 25:**
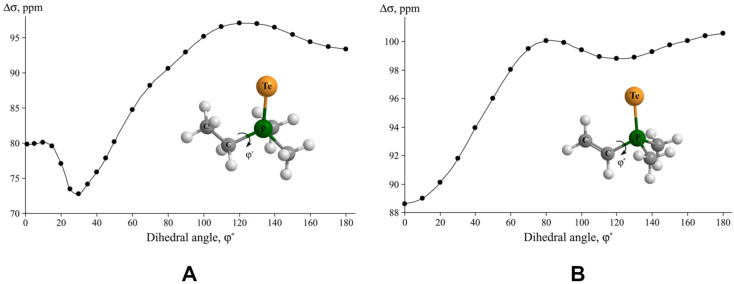
The dependence of the relativistic HALA effect on *σ*(^31^P) in Et(Me)_2_PTe plot (**A**) and Vin(Me)_2_PTe plot (**B**) on the dihedral angle *φ* = Te–P–C–C, obtained at the four-component density functional theory (KT1)/dyall.av3z level. Reproduced from Rusakova et al. [251] with the permission of John Wiley & Sons, Inc.

To demonstrate that, several cases modeling the deformation of π-LP(Te) were taken into consideration and canonical molecular orbital (CMO) analysis was been performed. In particular, the dependences of the relativistic HALA effect on *σ*(^31^P) in Et(Me)_2_PTe and Vin(Me)_2_PTe on the dihedral angle *φ* = Te–P–C–C, were calculated at the four-component DFT(KT1)/dyall.av3z level of theory. The corresponding graphs are presented in Figure 25 (see plots A and B, respectively). In these cases, the largest relativistic HALA effect is reached when bulky alkyl or alkene substituents are turned away from tellurium, and the smallest HALA effect is observed when they are oriented towards tellurium.

**Figure 26:**
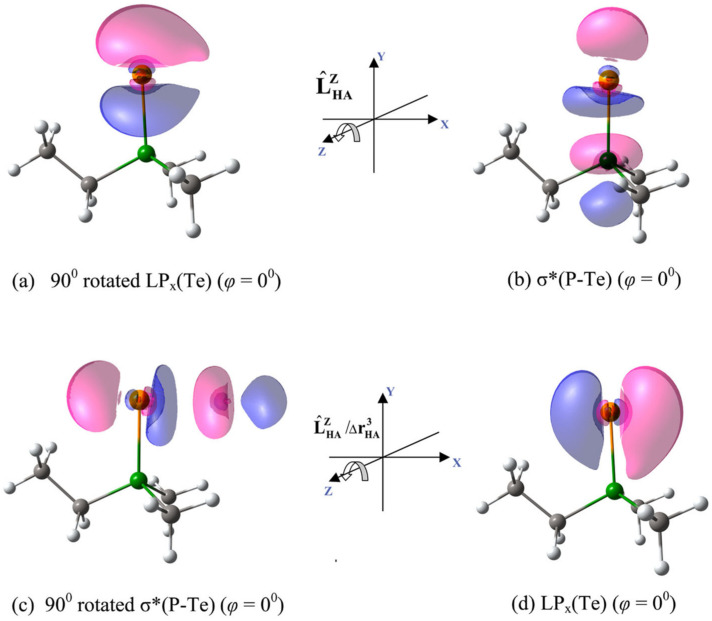
The isosurfaces of the near frontier occupied and unoccupied natural bond MOs responsible for the main part of the HALA effect in Et(Me)_2_PTe, with the π-LP_x_(Te) plot (**d**) and σ*(P–Te) plot (**b**) representing HOMO−1 and LUMO+1, respectively. Plots (**a**,**c**) show their 90-rotated isosurfaces around the *Z*-axis, as in accordance with the action rules of angular momentum operator. Reproduced from Rusakova et al. [251] with the permission of John Wiley & Sons, Inc.

The CMO analysis of the contributions to the HALA effect on *σ*(^31^P) in Et(Me)_2_PTe provided by various pairs of MOs has been carried out for several Te–P–C–C angles. This analysis revealed that the leading contribution to the HALA effect (which is 99% due to the SO-HALA term) is governed by the coupling of degenerate HOMO/HOMO-1 and LUMO/LUMO+1 orbitals that correspond to π-LP(Te) and σ*(P–Te). In accordance with its quantum chemical expression, its magnitude is determined by the MO integral between the 90-rotated HOMO/HOMO-1 and LUMO/LUMO+1 orbitals and vice versa. Therefore, if π-LP(Te) appears to be squashed for some reason, the overlap integral decreases and this diminishes the whole SO-HALA effect. This is demonstrated in Figure 26.

Thus, when alkyl or alkene substituents on phosphorus in Et(Me)_2_PTe or Vin(Me)_2_PTe, respectively, are oriented towards the tellurium atom, the squashing of π-LP(Te) is at its maximum, and the HALA effect appears to be suppressed the most, as is demonstrated in Figure 25. The same phenomenon is observed when going to more branched alkyl substituents in the R_3_PX series (X = O, S, Se, Te), which results in a substantially suppressed HALA effect [250].

**Figure 27:**
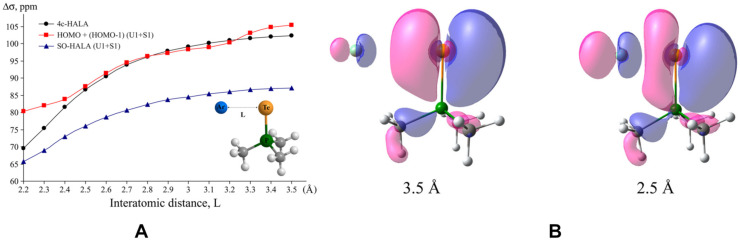
The dependences of the relativistic HALA and SO-HALA effects on the ^31^P NMR shielding constant in (Me)_3_PTe on the Te–Ar distance evaluated at the four-component GIAO-DFT(KT1)/dyall.av3z and the two-component ZORA-DFT (KT1)/TZP levels together with the total contribution of HOMO and HOMO−1 to the SO-HALA effect plot (**A**). The squashing of π-LP(Te) upon approaching argon atom by 1 Å plot (**B**). Reproduced from Rusakova et al. [251] with the permission of Wiley (Hoboken, NJ, USA).

Another considered example of deforming π-LP(Te) consisted of an argon atom approaching (Me)_3_PTe along the axis, which is perpendicular to the P–Te bond in (Me)_3_PTe and traverses the position of tellurium atom [251]. In this case, the HOMO is composed from π-LP(Te) with the fractions of the *π*-LP(Ar) and σ*(P–C) antibonding orbitals. The more closely argon approaches to tellurium atom, the more squashed π-LP(Te) turns out to be, resulting in diminishing the HALA effect on *σ*(^31^P). This is demonstrated in Figure 27, which shows the increase in relativistic correction with L(Te–Ar) (plot A) and the squashing of the *π*-LP(Te) upon changing the L(Te–Ar) from 3.5 Å to 2.5 Å (plot B).

Concluding this section, it should be mentioned that the relativistic effects on *σ*(^31^P)/*δ*(^31^P) can be very significant and their physical mechanisms are complicated and governed by many factors. The HAHA effect on *σ*(^31^P) is practically independent from the chemical environment and equals approx. 15–20 ppm. At the same time, the HALA effect on *σ*(^31^P) is strongly dependent on the type and heaviness of the atoms in the close vicinity to phosphorus. The magnitude of the HALA effect can reach hundreds of ppm and can be comparable with the nonrelativistic values of *σ*(^31^P) per se. They obviously should not be avoided in the calculations of systems containing atoms of the 3rd period and below in the periodic table, especially, when there are atoms with *π*-type lone electron pairs in the vicinity to phosphorus. The more *π*-type LPs on the heavy atoms located in the vicinity to phosphorus, the larger is the HALA effect. If, for some reason, the heavy atom *π*-type LPs turn out to be squashed, the HALA effect will be suppressed.

Generalizing the computational protocols applied nowadays for routine calculations of relativistic values of *σ*(^31^P)/*δ*(^31^P) or corrections to them, it can be said that the most popular approach is the four-component GIAO-DFT method applied in combination with the nonrelativistic Keal–Tozer KT1 and KT2 functionals. For the cases when the four-component methodology is too computationally expensive, the two-component ZORA calculations represent the most widespread alternative.

The most popular basis sets in the relativistic calculations of *σ*(^31^P)/*δ*(^31^P) are the dyall.(ac)vXz basis sets (X = 2, 3). However, it has been shown recently that the nonrelativistic *J*-oriented and *σ*-oriented basis sets, artificially saturated in the tight *s*-region, provide much better accuracy than the commensurate standard energy optimized or *σ*-optimized basis sets when calculating the relativistic SO-HALA corrections to the NMR shielding constants [352].

The most significant problem for now is the absence of readily available relativistic ab initio quantum chemistry methods with systematic accounting for the electron correlation effects devised for the NMR chemical shift calculations. Indeed, all relativistic calculations accounting for correlation effects are performed within the DFT method, therefore, one stumbles upon the same problems as those that persist at the nonrelativistic level. The most pronounced problem among all others is that the accuracy of the results substantially depends on the XC functional.

Potentially, the relativistic second-order polarization propagator approach (SOPPA) [353] can be very promising, as, to our knowledge, the nonrelativistic calculations within the SOPPA method within the CTOCD formalism can be carried out for NMR chemical shifts today. This opens an avenue for future development of accurate relativistic correlated ab initio methodology for the calculations of ^31^P NMR chemical shifts.

## 8. Vibrational Effects on ^31^P NMR Shielding Constants/Chemical Shifts

Molecular vibrations and rotations are the key movements that determine molecular infrared/Raman (rovibrational) spectra and are always the integrative part of experimentally observed molecular properties. Even at the temperature of absolute zero, the molecules have the so-called zero-point vibrations (ZPVs), which affect the NMR properties. Many approaches have been developed to take into account the rovibrational effects on molecular properties. Among the most important methodologies are those which use perturbation expansions to obtain vibrational frequencies and vibrationally averaged molecular properties [354,355,356,357] and those which resort to a conceptually different variational approach [358,359,360]. The most effective approach for the calculation of the vibrational wave function and zero-point vibrational corrections to molecular properties of polyatomic molecules was presented by Ruud, Åstrand, and Taylor [361,362]. They introduced an efficient automated procedure to calculate the rovibrationally averaged molecular geometries and a large number of second-order molecular properties, including the NMR shielding constants, using the SCF and multi-configurational self-consistent field (MCSCF) [363] wave functions.

According to Ruud, Åstrand, and Taylor, in order to take into account the effect of vibrations on a specific property, it is necessary to find an average of the property over the vibrational wave function. The property is expanded by a Taylor series around an arbitrary expansion point and represents a power series of mass-weighted displacements of the nuclei from the expansion geometry along the normal coordinates. The coefficients in the expansion are derivatives of various orders of the property at the expansion point with respect to normal coordinates. At the same time, vibrational wave function is determined from standard Rayleigh–Schrödinger vibrational perturbation theory. The vibrational perturbation theory uses the harmonic oscillator Hamiltonian as the zeroth-order Hamiltonian. The rest of Hamiltonian represents the perturbation. All expansions are applied to vibrational property averaging.

At that, the most effective approach is to use the effective geometry as the expansion point. This geometry corresponds to the ZPV-averaged geometry. In this case, the major contribution to the ZPV-averaged shielding constants that come from the anharmonicity of potential vanishes from direct consideration as it implicitly enters the property computed at a fixed effective geometry in the leading orders, and the ZPV-averaged shielding constant (${\langle \sigma \rangle }_{vib}$) represents the sum of the shielding constants calculated at the effective geometry (*σ*_eff_) and the term containing the second derivatives of *σ*_eff_ in relation to normal coordinates and reciprocal harmonic vibrational frequencies *ω*_K_ [364]:

(13) $${\langle \sigma \rangle }_{vib}=\sigma_{eff}+\frac{1}{4}\sum_{K}\frac{1}{\omega_{K}}\frac{d^{2}\sigma_{eff}}{dQ_{K}^{2}}$$

The rotational motions and finite nonzero temperature effects also provide contributions to the NMR shielding tensor. These contributions are much smaller in magnitude compared to the ZPV correction [154] and are rarely taken into consideration. The theory of these effects is much more complicated; for example, the vibrational averaging at a nonzero temperature involves the excited states of the vibrational wave function, assuming the averaging over the vibrational states of the molecule with the Boltzmann distribution. For detailed consideration of these effects, we can recommend a salient book chapter by Faber, Kaminsky, and Sauer [365].

The calculation of vibrational corrections to NMR chemical shifts is, for good reason, considered to be far more challenging than the calculation of these properties per se. Indeed, the calculations of vibrational corrections, even within the simplest approximation of the second-order vibrational perturbation theory (VPT2) [365], require the calculation of parameters defined as the third (cubic force constants) and fourth (shielding Hessians) derivatives of the electronic energy, and the number of such parameters, which must be calculated, increases rapidly with the number of atoms in the molecule. For now, the calculation of vibrational corrections to NMR chemical shifts remains the most challenging part that in the majority cases is just avoided due to unfeasibility of using the vibrational perturbation theory to perform vibrational averaging for systems consisting of more than a few atoms.

In this respect, molecular dynamics (MD) provide an alternative for accurate calculations of vibrational corrections for NMR chemical shifts by simulating atomic motions. In particular, this is essential for systems with large-amplitude motions like internal rotations. Such methods involve the averaging of shielding values from MD snapshots or use ab initio molecular dynamics (AIMD) to generate configurations, revealing substantial thermal effects and zero-point energy contributions. For now, except for only a few papers, one can hardly find the application of the MD method to the calculation of vibrationally averaged ^31^P shielding constants.

In particular, Fukal et al. [139] estimated the effects of molecular dynamics on ^31^P NMR chemical shifts in molecules **11**–**14** (see Figure 2) using molecular dynamics simulations with the GAFF (Generalized Amber Force Field) [366] and the TIP3P (transferable intermolecular potential with three points) water models [367], with statistical averaging of NMR chemical shift over MD snapshots, and, alternatively, by means of zero-point rovibrational averaging within the VPT2 theory carried out at the DFT(B3LYP)/IGLO-III level, taking into account only the harmonic contribution to the rovibrationally averaged value of σ(^31^P) at 300 K.

MD averaging has captured large-amplitude motions within phosphodiester linkage, while the usual rovibrational averaging that was carried out without considering solvent effects captured rather complex local dynamical motions of atoms near the energy minima. In both cases, the dynamical effects resulted in a decrease in the *σ*(^31^P) of all the considered molecules, though, the MD-averaging effect was less pronounced due to the simultaneous consideration of both solvent and rovibrational effects, which, if taken into account separately, typically impose the corrections of opposite signs. The calculated VPT2 rovibrational corrections to *σ*(^31^P) in the considered thiophosphates and phosphates (**11**–**14**) and reference compounds H_3_PO_4_ and PH_3_ were found to vary in the range from −16.53 to −1.26 ppm, depending on the molecule.

Field-Theodore et al. [154] explored the method and basis set effects on both the cubic force field calculation and the calculation of ^31^P NMR shielding derivatives in a rovibrational averaging problem for the PF_3_ molecule. The calculation of rovibrational effects on the *σ*(^31^P) of PF_3_ was carried out by means of applying the VPT2 theory at the MP2 and CCSD(T) levels and altering the basis set quality (triple- or quadruple-zeta) at different stages of the calculation, namely, in the cubic force field and the NMR shielding derivative calculation. Generally, it was found that there is only a little difference between the MP2- and CCSD(T)-calculated rovibrational and temperature effects. Moreover, the usage of the basis sets of triple- or quadruple-zeta quality resulted in almost similar rovibrational corrections, which prompted the authors to suggest that the MP2/triple-zeta basis set is sufficient for most purposes. In general, for all computational schemes, the vibrational and temperature effects on the *σ*(^31^P) of PF_3_ were found to be negative in sign, with ZPV correction being from −2.5 to −2.6 ppm and the temperature effect being approx. −0.5 ppm. It can be noted that the zero-point vibrational averaging effect is significantly larger in magnitude than the temperature effect. This finding is consistent with the results of the other studies, indicating the fact that the temperature effects are typically one order of magnitude smaller than the corresponding ZPV effects [368]. For example, Kupka et al. [156] calculated the ZPV and temperature effects on the *σ*(^31^P) of PH_3_, H_3_PO, and PN molecules and found that the temperature effects are very small in magnitude and have contributions to *σ*(^31^P) of ca. 4, 3, and 1% of the ZPV correction, respectively.

Rusakov et al. [250] calculated ZPV corrections to ^31^P NMR chemical shifts (relative to 85% H_3_PO_4_ scale with trimethyl phosphine used as an external standard) within the VPT2 at the MP2/ADZP level of theory to five tertiary phosphine chalcogenides, Me_3_P=X (X = O, S, Se) and t-Bu_3_P=X (X = Se, Te). The ZPV corrections in this series of molecules were found to vary from −11.7 ppm for Me_3_PO to −7.7 ppm for t-Bu_3_P=Te.

It is interesting to note that only in the case of phosphine chalcogenides with relatively light chalcogens, namely, Me_3_P=O and Me_3_P=S, the ZPV corrections significantly improved the agreement of calculated values with the experiment. In the rest of the molecules, which contained Se or Te, the ZPV corrections resulted in considerable underestimation (by ca. 10 ppm) of the *δ*(^31^P) values as compared to experimental values. Given rather high level of the overall computational scheme (CCSD/ATZP level for the basic values, MP2/ATZP level with the IEF-PCM for the solvent corrections, and the 4DFT-KT2/dyall.av3z level for the relativistic correctios), the problem, apparently, stems from considerable overestimation of the magnitude of ZPV corrections to *δ*(^31^P) for compounds containing heavy chalcogens. This may be indeed so, because a considerable influence of the relativistic effects initiated by heavy p-elements on the vibrational contributions to the shielding constants of neighbor nuclei was recently established in a few studies. In particular, it was found that in the presence of heavy atoms exhibiting strong spin–orbit (SO) interactions, significant changes in rovibrational motions of molecules take place. This produces large changes in the ZPV corrections to NMR shielding constants and chemical shifts, as was shown, for example, for ^1^H and ^13^C NMR chemical shifts of hydrogen halides [369,370] and dichalcogenides CX_2_ (X = O, S, Se, Te) [371,372], respectively. In particular, for the CTe_2_ molecule, the magnitude of the SO contribution to the ZPV correction to ^13^C NMR SC was found to be as much as 65% of the total nonrelativistic value of the ZPV correction. Sometimes, the SO effects can even change the sign of ZPV corrections to NMR SCs. Thus, for a proper evaluation of the ZPV corrections to the NMR SCs/CSs of molecules containing heavy elements, in general, and of phosphorus compounds with heavy elements in particular (especially in the case of important heavy transition metal complexes with phosphorus ligands), the ZPV corrections should be calculated at the relativistic level of theory.

Typical ZPV corrections to *σ*(^31^P) in different small phosphorus-containing compounds are gathered in Table 2. This table shows how different levels of theory used in the force field and shielding constant derivatives calculations can influence the final values of ZPV corrections to *σ*(^31^P) in representative molecules with substantially different electronic structures.

In general, the ZPV corrections to *σ*(^31^P) of different phosphorus-containing compounds vary in the range from −20 to −2 ppm, depending on molecule and the methodology used in the ZPV calculations. It is also evident that the theoretical values of ZPV corrections to *σ*(^31^P) in molecules which exhibit very strong electron correlation effects are affected to large extent by the method and basis set used at both stages of the ZPV calculation. This, in particular, can be seen from the results for molecules PN and HCP presented in Table 2. In the case of the PN molecule, the cardinal change in the ZPV correction emerges when switching between the HF, DFT, MP2, and CCSD(T) levels of theory. This brings about the changes in magnitude of the ZPV correction from −22.2 to −4.4 ppm. At the same time, in the case of HCP, the change in the DFT functional and the type and quality of the basis set used in the ZPV calculations practically results in a two-fold difference in the ZPV values.

It is worth noting that it is hard to predict what magnitude of the ZPV correction to *δ*(^31^P) may be expected for a given molecule, but one assumption can be proposed: the closer is the electronic structure of a particular fragment around the ^31^P nucleus under interest in a given compound to that of a standard, the lower will be the magnitude of the ZPV correction to the *δ*(^31^P) in this compound, because, in this case, the ZPV corrections of a standard and the compound will be effectively canceled out in accordance with IUPAC Formulas (9) and (10).

## 9. Popular Program Packages and Methods Allowing the NMR Shielding Constant Calculations

We suppose that having brief summary of the technical information about the program packages and methods that can be used for the ^31^P NMR chemical shift calculations might be helpful for studies modeling ^31^P NMR spectra when conducting structural studies of phosphorus compounds via the NMR method. Therefore, in this section, we have gathered information on the widely exploited program packages and quantum chemistry (QC) methods that can be used to calculate NMR shielding constants at nonrelativistic, quasirelativistic (two-component), and fully relativistic (four-component) levels, which is presented in Table 3. Please note that only widely used program packages are mentioned, while local or scarcely known codes are omitted.

## Figures and Tables

**Table 1:** Calculated CBS of *σ*(^31^P) values (in ppm) for molecules PH_3_ and PN ^1^.

Basis Set ^2^	DFT (B3LYP)	CCSD (T)
PH_3_		
aug-cc-pCVXZ (Q–5)	553.876	596.957
aug-cc-pCVXZ (T–5)	557.847	603.326
aug-pcSseg-*n* (2–4)	557.661	588.578
PN		
aug-cc-pCVXZ (Q–5)	−58.882	58.080
aug-cc-pCVXZ (T–5)	−60.030	59.090
aug-pcSseg-*n* (2–4)	−58.833	58.780

^1^ All data were taken from Kupka et al. [156] with the permission of MDPI. ^2^ Shown in parentheses are the points used in the basis set extrapolation to the CBS limit.

**Table 2:** ZPV corrections to *σ*(^31^P), in ppm, taken from different literature sources.

Mol.	Level of Theory Used in the Force Field Calculations	Level of Theory Used in Shielding Derivative Calculations	ZPV Correction to σ(^31^P)	Ref.
PH_3_	SVWN5/6-31+G(d)	SVWN5/6-31+G(d)	−9.2	Dransfeld [373]
	HF/cc-pVTZ	HF/qz3d1f	−9.5	Prochnow et al. [150]
	MP2/cc-pVTZ	MP2/qz3d1f	−8.2	
	CCSD(T)/cc-pVTZ	CCSD(T)/qz3d1f	−9.5	
	CCSD(T)/u-wCV5Z ^a^	CCSD(T)/u-wCV5Z ^a^	−9.2	Lantto [133]
	DFT(B3LYP)/aug-cc-pVQZ	DFT(B3LYP)/aug-cc-pVQZ	−10.8	Kupka et al. [156]
	DFT(B3LYP)/cc-pVDZ	DFT(B3LYP)/cc-pVDZ	−3.2	Chernyshev et al. [261]
	DFT(B3LYP)/IGLO-II	DFT(B3LYP)/IGLO-II	−3.9	
	DFT(B3LYP)/IGLO-III	DFT(B3LYP)/IGLO-III	−4.3	
PN	DFT(B3LYP)/aug-cc-pCVTZ	B3LYP/aug-cc-pCVTZ	−6.9	Teale et al. [152]
	HF/cc-pVTZ	HF/qz3d1f	−9.6	Prochnow et al. [150]
	MP2/cc-pVTZ	MP2/qz3d1f	(−)12.5 ^b^	
	CCSD(T)/cc-pVTZ	CCSD(T)/qz3d1f	−4.4	
	DFT(B3LYP)/aug-cc-pVTZ	DFT(B3LYP)/aug-cc-pVTZ	−7.6	Kupka et al. [155]
	DFT(B3LYP)/aug-cc-pVQZ	DFT(B3LYP)/aug-cc-pVQZ	−7.0	
	DFT(B3LYP)/aug-cc-pV5Z	DFT(B3LYP)/aug-cc-pV5Z	−7.5	
	CCSD(T)/aug-cc-pVTZ	MP2/aug-cc-pVTZ	−16.5	
	CCSD(T)/aug-cc-pVQZ	MP2/aug-cc-pVQZ	−14.2	
	CCSD(T)/aug-cc-pV5Z	MP2/aug-cc-pV5Z	−14.7	
	CCSD(T)/aug-cc-pVTZ	DFT(B3LYP)/aug-cc-pVTZ	−22.2	
	CCSD(T)/aug-cc-pVQZ	DFT(B3LYP)/aug-cc-pVQZ	−20.5	
	CCSD(T)/aug-cc-pV5Z	DFT(B3LYP)/aug-cc-pV5Z	−22.2	
	DFT(B3LYP)/aug-cc-pVQZ	DFT(B3LYP)/aug-cc-pVQZ	−6.2	Kupka et al. [156]
PF_3_	MP2/tz2p	MP2/tz2p	−2.6	Field-Theodore et al. [154]
	MP2/tz2p	MP2/qz2p	−2.6	
	MP2/qz2p	MP2/qz2p	−2.5	
	CCSD(T)/tz2p	CCSD(T)/tz2p	−2.6	
	CCSD(T)/tz2p	CCSD(T)/qz2p	−2.50	
	HF/cc-pVTZ	HF/qz3d1f	−1.4	Prochnow et al. [150]
	MP2/cc-pVTZ	MP2/qz3d1f	−2.3	
	CCSD(T)/cc-pVTZ	CCSD(T)/qz3d1f	−2.3	
HCP	SVWN5/6-31+G(d)	SVWN5/6-31+G(d)	−50.7	Dransfeld [373]
	B3LYP/aug-cc-pCVTZ	B3LYP/aug-cc-pCVTZ	−22.9	Teale et al. [152]
CH_3_PH_2_	SVWN5/6-31+G(d)	SVWN5/6-31+G(d)	−44.7	Dransfeld [373]
CH_3_CP	SVWN5/6-31+G(d)	SVWN5/6-31+G(d)	−24.0	
P_4_	HF/cc-pVTZ	HF/qz3d1f	−5.3	Prochnow et al. [150]
	MP2/cc-pVTZ	MP2/qz3d1f	−6.9	
	CCSD(T)/cc-pVTZ	CCSD(T)/qz3d1f	−7.2	
Me_3_P	HF/cc-pVTZ	HF/qz3d1f	−13.5	Prochnow et al. [150]
	MP2/cc-pVTZ	MP2/qz3d1f	−15.1	
	MP2/ADZP	MP2/ADZP	−16.3	Rusakov et al. [250]
	DFT(B3LYP)/cc-pVDZ	DFT(B3LYP)/cc-pVDZ	−11.7	Chernyshev et al. [261]
	DFT(B3LYP)/IGLO-II	DFT(B3LYP)/IGLO-II	(−)14.1 ^b^	
	DFT(B3LYP)/IGLO-III	DFT(B3LYP)/IGLO-III	−14.9	
H_3_PO	DFT(B3LYP)/aug-cc-pVQZ	DFT(B3LYP)/aug-cc-pVQZ	−4.7	Kupka et al. [156]
Me_3_PO	MP2/ADZP	MP2/ADZP	−4.6	Rusakov et al. [250]
Me_3_PS	MP2/ADZP	MP2/ADZP	−6.8	
Me_3_PSe	MP2/ADZP	MP2/ADZP	−7.5 ^c^	
*t*-Bu_3_PSe	MP2/ADZP	MP2/ADZP	−7.5 ^c^	
*t*-Bu_3_PTe	MP2/ADZP	MP2/ADZP	−8.6 ^c^	

^a^ The u-wCV5Z basis set was supposedly used in the ZPV correction calculation by Lantto, but this information was not directly mentioned in the article. ^b^ The negative sign has been added to this ZPV correction, assuming that the misprint took place in the original literature source. ^c^ These values were obtained at the nonrelativistic level of theory, while including the spin–orbit relativistic effects might substantially change these values.

**Table 3:** Popular program packages and methods allowing for NMR shielding constant calculations.

Program Package	Available QC Methods	Available Basis Sets	Notes
CFOUR (Coupled-Cluster techniques for Computational Chemistry) [374]	Nonrelativistic HF-SCF; MP*n* (*n* = 2, 3, 4); CCD [375], CC2, CC3, CCSD, CCSD(T), CCSDT-*n* (*n* = 1–4) [376], CCSDT; QCISD, QCISD(T) [377].	Apart from built-in standard GTO (Gaussian-Type Orbitals) energy-optimized basis sets, one can use specialized σ-oriented basis sets by preparing an external basis set file. The specialized basis sets can be taken from the literature sources or from the external library [378,379,380].	CFOUR allows only gas-phase calculations that can be performed within the GIAO formalism.
ADF (Amsterdam Density Functional) [381,382]	The DFT with standard LDA and GGA potentials, implemented at the relativistic scalar-ZORA and SO-ZORA (or briefly ZORA) levels.	ADF commonly uses the basis sets of STOs (Slater-Type Orbitals). For shieldings, only standard relativistic and nonrelativistic energy-optimized basis sets of various levels and types can be applied.	The GIAO formalism is available. Solvent calculations can be performed with the IEF-PCM or COSMO model. The NBO (Natural Bond Orbital) [383,384] analysis of the NMR shielding tensor is available.
DIRAC (Program for Atomic and Molecular Direct Iterative Relativistic All-electron Calculations) [221]	HF-SCF and DFT with LDA and various GGA and hybrid XC functionals, implemented at the two-component (DKH2, BSS, and X2C) and four-component relativistic levels.	DIRAC includes a great many relativistic and nonrelativistic energy-optimized GTO-type basis sets. The specialized σ-oriented basis sets can be included through the external file.	The GIAO formalism is available. Relativistic IEF-PCM for taking into account the solvent corrections can be applied. Nonrelativistic regimes are simulated either within the Lévy-Leblond scheme [385] or by increasing the speed of light multiple times. The scalar calculations can be performed with the spin-free Hamiltonians.
ReSpect (Relativistic Spectroscopy DFT program package) [386]	Four-component Hartree–Fock (DHF) and four-component DFT (DKS) with LDA, GGA, hybrid, and range-separated XC functionals.	ReSpect provides a variety of built-in all-electron basis sets of the GTO-type suitable for relativistic calculations of elements across the periodic table (Z = 1–118).	Either common gauge origin (CGO) or GIAO formalism can be applied in combination with the restricted kinetic magnetic balance condition (RMB).
ORCA [387]	Nonrelativistic HF-SCF, RI-MP2, and DFT with LDA and various GGA, hybrid, meta-GGA, and double-hybrid XC functionals.	A restricted number of GTO basis set families are built in the ORCA program, which includes Ahlrichs, Dunning, and Pople’s basis sets of different levels.	The GIAO method is available. The SMD (Solvation Model based on Density) [388] or C-PCM (Conductor-like Continuum Polarization Model, factually, COSMO) models can be applied to model solvent.
TURBOMOLE [151]	Nonrelativistic HF-SCF and DFT with LDA, GGA, meta-GGA, and range-separated hybrid XC functionals.	TURBOMOLE has its own built-in basis set library. Available basis sets are of Ahlrichs GTO-type, given in segmented contracted form.	The GIAO formalism is implemented. The COSMO calculations can be performed to account for the solvent effects. Nuclear-independent chemical shifts (NICSs) [389] analysis is available.
Gaussian [390]	Nonrelativistic HF-SCF, MP2, and DFT with LDA, GGA, hybrid, and double-hybrid XC functionals.	Gaussian provides a great number of nonrelativistic energy-optimized GTO-type basis sets. The specialized σ-oriented basis sets can be implemented through the external file or via direct input.	The CSGT or GIAO formalisms are available. Standard solvation models such as IEF-PCM, C-PCM, COSMO, and SMD can be applied to account for solvent effects. The ONIOM (QM/MM method) of up to three layers is also available.
QChem [391]	Nonrelativistic HF-SCF and DFT with LDA, GGA, and hybrid XC functionals.	QChem supports the user-defined basis sets.	The GIAO formalism is implemented.
Dalton [392]	Nonrelativistic HF-SCF, DFT, MCSCF, and various CC levels of theory (through the response functions). The SOPPA [393], SOPPA(CC2) [394], and SOPPA(CCSD) [393,394] methods are applicable for shielding constants with the CTOCD formalism. Relativistic corrections can be calculated by means of the linear response with elimination of the small component method (LRESC) [395,396,397,398].	Available basis sets are the same as in DIRAC program. The inclusion of the external basis sets is also feasible.	The GIAO and CTOCD formalisms are available. Solvent effects on NMR parameters can be approximated in the HF-SCF and DFT levels with the IEF-PCM. In addition, the QM/MM-type embedding models are applicable to the calculation of GIAO NMR shielding constant calculations.

## Data Availability

No new data were created or analyzed in this study. Data sharing is not applicable to this article.
